# LAPF enhances lysosomal acidification to promote TLR9 and cGAS-STING-mediated antiviral immunity and attenuate HSV-1-induced neuroinflammatory pain

**DOI:** 10.1186/s12974-026-03856-6

**Published:** 2026-05-08

**Authors:** Erliang Kong, Mengqiu Deng, Ruifeng Ding, Mei Yang, Yongchang Li, Xudong Feng, Honghao Song, Huawei Wei, Xin Jiang, Hongbin Yuan, Chaofeng Han

**Affiliations:** 1 Department of Anesthesiology, Shanghai Changzheng Hospital, Second Affiliated Hospital of Naval Medical University, 415 Fengyang Road, Shanghai, 200003 P.R. China; 2 Department of Anesthesiology, the 988th Hospital of Joint Logistic Support Force of Chinese People’s Liberation Army, Zhengzhou, Henan 450042 China; 3 Faculty of Medical Imaging, Naval Medical University, Shanghai, 200433 China; 4 Department of Histology and Embryology, National Key Laboratory of Immunity and Inflammation, Naval Medical University, 800 Xiangyin Road, Shanghai, 200433 P.R. China

**Keywords:** Postherpetic neuralgia, Neuroinflammation, Antiviral innate immunity, Lysosomal acidification, Lysosomal membrane stability, LAPF

## Abstract

Postherpetic neuralgia (PHN) is characterized by neural injury and neuroinflammation resulting from viral infection and reactivation. Herpes simplex virus type 1 (HSV-1) is capable of inducing virus-associated PHN-like neuropathic pain and has been widely used as a model for studying virus-induced neuroinflammatory pain. However, the immune mechanisms underlying virus-induced neuroinflammation and pain remain incompletely understood. In this study, we used an HSV-1-induced neuroinflammatory pain model and observed reduced *Lapf* expression following HSV-1 infection through transcriptome sequencing, which was further confirmed to be localized in microglia of the spinal dorsal horn by immunofluorescence staining. *Lapf* microglia-specific deficiency aggravated neuroinflammation and promoted mechanical allodynia by impairing antiviral innate immunity both in vivo and in vitro. Overexpression of *Lapf* in microglia strengthened antiviral innate immunity and suppressed HSV-1 replication. Mechanistically, transcriptome sequencing of *Lapf* microglia-specific deficient mice identified lysosomal endocytosis as a critical pathway in LAPF-mediated antiviral innate immunity. *Lapf* deficiency decreased lysosomal acidity, resulting in reduced TLR9 activation, thereby impairing viral DNA sensing and IFN-I production. *Lapf* deficiency also reduced lysosomal membrane stability, facilitating the escape of HSV-1 DNA into the cytoplasm, where it could amplify and reactivate. Conversely, *Lapf* overexpression enhanced lysosomal acidity and membrane stability, promoting TLR9 activation and antiviral innate immunity. Furthermore, *Lapf* deficiency markedly reduced the phosphorylation of STING, TBK1, and IRF3, whereas *Lapf* overexpression restored cGAS-STING signaling. This effect was abolished by lysosomal acidification inhibitor chloroquine (CQ), supporting that LAPF promotes lysosomal acidification-dependent antiviral immunity via TLR9 and cGAS-STING pathways. Pharmacological enhancement of LAPF activity using the dephosphorylation inhibitor SHP099 alleviated neuroinflammation and mechanical allodynia in HSV-1-induced neuroinflammatory pain model mice, suggesting potential therapeutic implications. In conclusion, our findings demonstrate that LAPF enhances lysosomal acidification to promote dual antiviral innate immune responses via TLR9 and cGAS-STING pathways in HSV-1 infection, thereby attenuating HSV-1-induced neuroinflammatory pain. These results provide mechanistic insights and potential therapeutic targets for virus-associated neuroinflammatory pain.

**Supplementary Information:**

The online version contains supplementary material available at 10.1186/s12974-026-03856-6.

## Introduction

Neuropathic pain associated with viral infection is often characterized by persistent tactile allodynia, neuroinflammation, and neuronal injury [1, 2]. Among neurotropic viruses, herpes simplex virus type 1 (HSV-1) has been shown to induce long-lasting neuroinflammatory pain and is widely used as an experimental model to investigate virus-associated neuropathic pain. Despite its clinical and experimental relevance, the molecular mechanisms that regulate antiviral immunity and neuroinflammation in the central nervous system (CNS) during HSV-1 infection remain incompletely understood [3, 4]. In particular, dysregulated host antiviral responses in aged or immunocompromised conditions can facilitate viral persistence and exacerbate neuroinflammatory damage, ultimately contributing to chronic pain [3–6]. Current therapeutic strategies provide limited relief, highlighting the need to identify novel molecular regulators that coordinate antiviral defense and neuroinflammatory responses.

HSV-1 is a neurotropic α-herpesvirus that establishes latency in sensory ganglia and can reactivate under conditions of impaired immunity [7, 8]. Following infection, host innate immune responses, particularly type I interferon (IFN-I) signaling, play a critical role in restricting viral replication and spread. However, HSV-1 has evolved multiple immune evasion strategies to subvert these antiviral defenses [9, 10]. Our previous study demonstrated that HSV-1 induces PRMT6 expression in spinal microglia, leading to methylation-dependent inhibition of the cytosolic DNA sensor STING and suppression of TBK1-IRF3-IFN-I signaling, thereby promoting viral persistence and neuroinflammation [11]. These findings highlight the importance of microglial dysfunction in antiviral immune failure, yet the upstream regulatory mechanisms governing viral sensing and immune activation in microglia remain to be fully elucidated.

Lysosomes serve as critical hubs for antiviral innate immunity, particularly in microglia, the resident immune cells of the CNS [12]. Through endocytosis and phagocytosis, microglia sequester viral particles into lysosomes, where an acidic environment is essential for the activation of pattern-recognition receptors such as Toll-like receptor 9 (TLR9). TLR9 resides within lysosomes and requires an acidic pH to undergo proteolytic maturation and subsequently sense unmethylated CpG DNA from engulfed viruses, triggering IFN‑I production that restricts viral replication [13, 14]. In parallel, lysosomal membrane integrity is essential for preventing viral DNA escape into the cytoplasm. Disruption of lysosomal stability may allow viral genomes to access cytosolic DNA sensors such as cGAS-STING, while also promoting viral replication and excessive inflammatory responses [15]. Emerging evidence suggests that lysosomal homeostasis regulates both TLR9-dependent and cGAS-STING-dependent pathways, indicating that lysosomal dysfunction could simultaneously impair multiple antiviral immune axes [16, 17].

LAPF (also known as PLEKHF1) is a lysosome-associated adaptor protein containing FYVE and PH domains that regulates endosomal trafficking, phagosome maturation, and lysosomal acidification [18, 19]. Previous studies have shown that LAPF supports bacterial clearance in macrophages by maintaining lysosomal function [20, 21]; however, its role in antiviral immunity, particularly in microglia during neurotropic viral infection, remains largely unknown. Given the central role of lysosomes in antiviral immune sensing, we hypothesized that LAPF may act as a key regulator of lysosome-dependent antiviral responses in microglia during HSV-1 infection.

In this study, we employed an HSV-1-induced neuroinflammatory pain model and found that HSV-1 infection markedly downregulates LAPF expression in spinal microglia, leading to impaired lysosomal acidification and reduced membrane stability. This dysfunction attenuates TLR9 activation and IFN-I production, while facilitating viral DNA leakage into the cytoplasm. Notably, LAPF deficiency also compromises activation of the cGAS-STING pathway, indicating that both lysosomal and cytosolic antiviral responses depend on intact LAPF-mediated lysosomal function. These defects collectively promote viral replication, neuroinflammation, and neuronal injury, ultimately contributing to HSV-1-induced neuroinflammatory pain. Importantly, pharmacological enhancement of LAPF activity using the dephosphorylation inhibitor SHP099 alleviated neuroinflammation and mechanical allodynia, suggesting potential therapeutic relevance. Together, our findings identify LAPF as a critical regulator of microglial antiviral immunity and provide mechanistic insight into virus-induced neuroinflammatory pain.

## Materials and methods

### Mice

We procured male C57BL/6J mice (20–25 g) from the Experimental Animal Center at Naval Medical University (Shanghai, China). We utilized two distinct mouse strains for the creation of conditional knockout mice targeting *Lapf* specifically in microglia: *Tmem119*^*Cre*^ and *Lapf* ^*flox/flox*^. *Tmem119*^*Cre*^ mice expressed *Cre* recombinase under the *Tmem119* promoter, allowing for specific targeting of microglial cells. *Lapf* ^*flox/flox*^ mice were designed with two *loxP* sites flanking critical exons of the *Lapf* gene, enabling conditional gene knockout. To generate microglial conditional knockout mice *Tmem119*^*Cre+*^*Lapf* ^*flox/flox*^ (*Lapf*-CKO) and littermate control mice *Tmem119*^*Cre−*^*Lapf* ^*flox/flox*^ (*Lapf*-WT), we implemented a breeding strategy involving male *Tmem119*^*Cre*^ mice crossed with female *Lapf* ^*flox/flox*^ mice. Offspring resulting from this crossbreeding were screened for the presence of both the *Tmem119*^*Cre*^ and *Lapf* ^*flox*^ alleles, confirming their conditional knockout genotype. Genotyping was carried out through polymerase chain reaction using genomic DNA isolated from tail biopsies of the mice. *Lapf*-WT were employed as controls. Specific primer sets were devised to detect the presence of the *Tmem119*^*Cre*^ and *Lapf* ^*flox*^ alleles in the mice. The *Cre* recombinase primer sequences were: forward, 5’-CCCAGAAATGCCAGATTACG-3’, reverse, 5’-CTTGGGCTGCCAGAATTTCTC-3’; the *flox* primer sequences were: forward, 5’-TCTTCCGATTATAGACCGTCCCA-3’, reverse, 5’-TTCAGCCAGCCCACTTAGCC-3’. All mice were housed within a sterile facility, maintained under controlled environmental conditions with a room temperature of 24 °C, a humidity level of 50%, and a 12/12-hour light/dark cycle. The mice were provided with unrestricted access to both food and water. Throughout the study, we made rigorous efforts to minimize the use of animals and to ensure their comfort and welfare. The study protocol obtained ethical approval from the Animal Ethics Committee at Naval Medical University.

### Reagents

This study utilized the following reagents at the specified concentrations: Chloroquine (CQ, 10 µM, Sigma, C6628) was used to inhibit acidification of the lysosomal compartment and prevents cargo degradation in lysosomes. A synthetic TLR9 ligand, C-type oligonucleotide containing unmethylated CpG sequences (CpG-C ODN, MedChemExpress, HY-150743) was used as TLR9 agonist. Moreover, INH-1 ODN (MedChemExpress, HY-153841), a TLR9 synthetic inhibitory oligonucleotide, was employed to inhibit TLR9 pathway. The inhibitor of the tyrosine phosphatase SHP2, SHP099, which can increase LAPF phosphorylation, was administered intrathecally (0.1 µg/5 µL, MedChemExpress, HY-100388) for three consecutive days on Day 14 after HSV-1 infection. The following antibodies were used were: anti- LAPF antibody (Abmart, RG503775), anti-IBA-1 antibody (abcam, ab178846), anti-GFAP antibody (abcam, ab7260), anti-NeuN antibody (abcam, ab177487), anti-LAMP1 antibody (marker of lysosome, Cell Signaling Technology, 99437), anti-CTSD antibody (the maturated form of lysosomal hydrolase cathepsin D, Cell Signaling Technology, 88239), anti-STING antibody (Cell Signaling Technology, 13647), anti-*p*-STING antibody (Cell Signaling Technology, 72971), anti-TBK1 antibody (Cell Signaling Technology, 38066), anti-*p*-TBK1 antibody (Cell Signaling Technology, 5483), anti-IRF3 antibody (Cell Signaling Technology, 4302), anti-*p*-IRF3 antibody (Cell Signaling Technology, 29047). LysoTracker Red (100 nM, Invitrogen, L12492) was used as the lysosomal acidification indicator. Acridine orange (AO, 5 µM, Sigma, A9231) and CTSD level were used to evaluate lysosomal membrane stability.

### Establishment of HSV-1-induced neuroinflammatory pain model

The HSV-1-induced neuroinflammatory pain model was induced in mice through infection with HSV-1 (strain 7401 H), following established protocols as previously reported [22]. Initially, the hair on the caudal back, flank, and hind limbs of the mice was trimmed and removed using a chemical depilatory agent. After a three-day interval, a 27-gauge needle was employed to create a superficial epidermal injury on the right shin, followed by the administration of HSV-1 (1 × 10^6^ PFU in 10 µL). For the mock infection control, HSV-1 was first inactivated by incubation at 60 °C for 1 h. The assessment of skin lesions was conducted using the following scoring system: 0 for the absence of lesions, 2 for the presence of one or two vesicles on the back, 4 for the presence of numerous vesicles surrounding the infected area, 6 for mild herpes zoster-like lesions, 8 for evident zoster-like lesions or paw inflammation, and 10 for severe zoster-like lesions. To evaluate the viral load, the thymidine kinase (TK) gene of HSV-1 in the spinal dorsal horn was analyzed using quantitative polymerase chain reaction (*Q*-PCR).

### RNA sequencing and data analysis

RNA sequencing and subsequent data analysis were performed using spinal dorsal horn samples collected from mice. Immediately after sample collection, the specimens were flash-frozen in liquid nitrogen to facilitate mRNA microarray analysis. Subsequently, mRNA was reverse-transcribed to generate complementary DNA (cDNA), followed by the construction of an amplified cDNA library. High-throughput sequencing was conducted on the Illumina NovaSeq6000 platform, utilizing paired-end sequencing to generate transcriptome expression data. Differentially expressed genes (DEGs) were identified between experimental groups using the DESeq2 package in R. DEGs were defined as genes with |log_2_(fold change)| > 1 and adjusted *P*‑value < 0.05. Hierarchical cluster analysis (HCA) was performed using the pheatmap package in R; Euclidean distance was used to measure gene expression similarity, and complete linkage method was applied for sample clustering. Principal component analysis (PCA) was conducted using the stats package in R to evaluate sample repeatability and group separation. PCA was performed using normalized transcript counts (FPKM) of the whole transcriptome (all detected genes), without pre-selection of highly variable transcripts. For functional annotation and pathway enrichment analysis, the R package clusterProfiler was employed for Gene Ontology (GO) annotation analysis and Kyoto Encyclopedia of Genes and Genomes (KEGG). Immune-related DEGs were selected from total DEGs based on functional annotation associated with antiviral immunity, type I interferon signaling, and inflammatory responses. The RNA sequencing data generated in this study have been deposited in the Sequence Read Archive (SRA) database under the accession numbers PRJNA1121985 and PRJNA1121986.

### Western blotting analysis

For the assessment of protein expression, we obtained samples from both the spinal dorsal horn and cultured microglia. To achieve comprehensive protein extraction, the specimens were subjected to sonication in ice-cold RIPA lysis buffer, and protein concentrations were quantified using a BCA kit. Subsequently, the proteins were denatured by incubation at 99 °C for 10 min, followed by separation on 10% SDS-PAGE gels and transfer onto PVDF membranes. These PVDF membranes were then subjected to a 2-hour blocking step with 5% bovine serum albumin. Following blocking, the membranes underwent an overnight incubation at 4 °C with primary antibodies. Subsequently, a 2-hour incubation with secondary antibodies was carried out, enabling the visualization of protein bands through enhanced chemiluminescence.

### *Q*-PCR

For RNA extraction, we collected samples from both the spinal dorsal horn and cultured microglia. Total RNA was isolated using the TRIzol method, followed by reverse transcription to generate complementary DNA (cDNA) using a reverse transcription kit. Subsequently, Q-PCR was performed with the SYBR Green kit, utilizing the QuantStudio 5 instrument (Applied Biosystems). The cycle threshold (CT) values of the target genes were determined and then normalized to the expression of β-actin. These normalized values were employed to calculate gene expression fold changes using 2^−ΔΔCT^ method. Detailed primer sequences used in these experiments are listed in supplementary Table 1.

### Immunofluorescence microscopy

For immunofluorescence analysis, mice were euthanized in accordance with humane protocols and then subjected to perfusion with sterile saline, followed by 4% paraformaldehyde. The lumbar segments L4-5 of the spinal cord were promptly harvested, postfixed in 4% paraformaldehyde for 24 h, and subsequently immersed in a 30% sucrose solution for dehydration. Transverse frozen tissue sections, each with a thickness of 20 μm, were prepared using a freezing microtome (Leica, Germany). To block non-specific binding sites, these sections were then incubated with 5% goat serum for 2 h. Subsequently, the spinal cord sections were incubated overnight at 4 °C with primary antibodies, followed by a 2-hour incubation with secondary antibodies. Immunofluorescence images were acquired using a Zeiss LSM710 confocal microscope. Subsequently, these images were merged using Adobe Photoshop software for further analysis. For lysosomal fluorescence imaging (LysoTracker Red and Acridine Orange), three independent biological replicates were performed for each group. For each sample, three random visual fields were captured under the same imaging parameters, and the most representative images were displayed. Fluorescence intensity was quantitatively analyzed using ImageJ software.

### Cultivation of primary microglia and BV-2 microglial cells

To establish sterile conditions, neonatal mice were humanely euthanized under anesthesia before isolating the spinal dorsal horn from the lumbar segments. The dissected spinal dorsal horn was gently rinsed with cold PBS, fragmented, and enzymatically digested using 0.25% trypsin at 37 °C for 10 min. To terminate the digestion process, 10% FBS/DMEM was added. Single-cell suspensions were generated from the samples through gentle pipetting, followed by centrifugation to collect cellular pellets. These cellular pellets were then suspended in 10% FBS/DMEM and seeded into T-25 flasks coated with poly L-ornithine for cultivation in a 37 °C incubator with 5% CO_2_. Daily medium changes were carried out over an 8-day period. Subsequently, the T-25 flasks were agitated at 200 rpm for 5 min to separate microglia from astrocytes and neurons. The medium was transferred to a fresh tube for centrifugation, and the resulting cellular pellets were gently collected and resuspended in fresh medium for subsequent functional assays. The BV-2 microglial cell line, generously provided by Naval Medical University (Shanghai, China), was cultured under standard conditions (37 °C and 5% CO_2_) in 10% FBS/DMEM. Daily medium replacements were performed until cellular confluence reached 80%.

### Enzyme-linked immunosorbent assay (ELISA)

To assess the levels of target protein, reagents provided by the ELISA Kit were prepared following the manufacturer’s instructions. Microplate wells were coated with 100 µL of the provided capture antibody specific to the target proteins and incubated overnight at 4 °C. Afterward, the capture antibody solution was aspirated, and the wells were washed three times with the supplied wash buffer. To block non-specific binding, 300 µL of assay diluent solution was added to each well and incubated at room temperature for 1 h. Samples and a series of standard concentrations were prepared. For each well, 100 µL of appropriately diluted standards or samples was added. The plates were sealed and incubated at room temperature for 2 h. After incubation, the plates were washed, and 100 µL of the detection antibody solution provided in the kit was added to each well. The plates were sealed and incubated for 1 h at room temperature. Following another round of washing, 100 µL of the streptavidin-horseradish peroxidase (HRP) conjugate solution from the kit was added to each well. The plates were sealed and incubated for 30 min at room temperature. The colorimetric reaction was initiated by adding 100 µL of substrate solution to each well. Plates were incubated in the dark until a blue reaction product became visible, and the reaction was stopped with 100 µL of stop solution. Absorbance at the target wavelength was measured for each well using a microplate reader. Experimental data were analyzed by comparing absorbance values with a standard curve generated from known concentrations of standards included in the kit. The ELISA kits used were as follows: IFN-α Mouse ELISA Kit (Invitrogen, BMS6027), IFN-β Mouse ELISA Kit (Invitrogen, 424001), TNF-α Mouse ELISA Kit (Invitrogen, 88-7324-88), and IL-1β Mouse ELISA Kit (Invitrogen, 900-M47).

### Measurements of mechanical pain thresholds

The assessment of mechanical pain thresholds took place in a tranquil environment. Mice were provided a 2-hour period to acclimatize to their surroundings within Plexiglas™ cages with wire mesh floors. Von Frey filaments (0.6 g and 2 g, North Coast Medical, Gilroy, CA, USA) were gently applied to the plantar skin, skin lesions, and abdominal skin. The 0.6 g and 2 g filament were employed for low-force and high-force stimulation to evaluate pain allodynia. This testing procedure was repeated six times with a 60-second interval. Scoring was conducted according to the following criteria: 0 for no response, 1 for lifting of the stimulated area, and 2 for flinching or licking of the stimulated area. The pain-related score (%) was calculated using the formula: Pain-related score (% of max score) = [(sum of score)/12]×100 [23, 24]. HSV-1 induced zoster-like skin lesions within 7 to 10 days post-infection. By Day 14, the skin lesions had completely healed, leaving residual scars. Therefore, mice displaying mechanical allodynia after 14 days were considered to have developed PHN-like neuropathic pain.

### Transmission electron microscopy (TEM)

For in-depth ultrastructural examination, mice were humanely euthanized and underwent perfusion with a PBS solution followed by 0.25% glutaraldehyde. Lumbar spinal cord segments (L4-5) were rapidly excised and immersed in a 2.5% glutaraldehyde solution. These tissue specimens were subsequently sectioned into 1 mm^3^ blocks using a vibratome. Post-fixation was carried out in a 1% osmium tetroxide solution for 1 h. Dehydration was achieved through sequential immersion in ethanol solutions with increasing concentrations. The dehydrated tissue blocks were then embedded in epoxy resin and polymerized at 80 °C for 24 h. Ultrathin sections, approximately 60 nm thick, were obtained from the resin-embedded blocks using an ultramicrotome. These sections underwent additional enhancement through post-staining with uranyl acetate and lead citrate to improve contrast. Finally, the prepared samples were examined using a transmission electron microscope (Thermo Scientific Talos F200S). This methodology fully adheres to TEM standards, allowing for precise ultrastructural analysis of spinal cord tissues.

### siRNA construction and transfection

Custom-designed siRNAs targeting mouse *Lapf* (si-*Lapf*) and negative control siRNA (Mock) were synthesized by OBIO Biotechnology (Shanghai). BV-2 cells were cultured in 24-well plates until they reached a density of 2 × 10^4^ cells/cm^2^, achieving 30–50% confluence. To facilitate transfection, siRNA molecules were diluted in Opti-MEM, while Lipofectamine™ 2000 transfection reagent (Invitrogen, 11668019) underwent dilution in Opti-MEM as well. Afterward, these solutions were combined and introduced into the 24-well plate. BV-2 cells were then subjected to incubation for 6 h in a culture medium that comprised 10% FBS/DMEM. Post-incubation, the medium was replaced, allowing BV-2 cells to continue their cultivation for an additional 48 h. Evaluation of knockdown efficiency was executed through Western blotting and *Q*-PCR analysis.

### Plasmid construction and cell transfection

DNA fragments encoding mouse *Lapf* were meticulously designed and synthesized by OBIO Biotechnology (Shanghai), followed by comprehensive sequence validation. BV-2 cells were cultured in 24-well plates until they reached a robust confluence of 80–90%. The plasmid constructs were precisely diluted in Opti-MEM, and Lipofectamine™ 2000 transfection reagent was similarly prepared in Opti-MEM. These carefully prepared solutions were combined and introduced into the 24-well plate to facilitate transfection. Subsequently, BV-2 cells were incubated in a culture medium composed of 10% FBS/DMEM for 48 h. The efficacy of overexpression was meticulously assessed through Western blotting and *Q*-PCR analyses.

### Statistical analysis

For quantitative *Q*-PCR analysis, mRNA expression levels of target genes were first normalized to the housekeeping gene GAPDH. For Western blotting analysis, protein expression levels were first normalized to the housekeeping protein GAPDH. For statistical analysis and graph presentation, one representative sample in the control group was defined as 1.0, and all other samples were calculated as fold changes relative to this control sample. The first bar in each bar graph represents the control group. We performed statistical analyses using IBM SPSS Statistics 23.0, and data are expressed as mean ± standard deviation (SD). Each experiment was performed in triplicate technical replicates, and the average value of technical replicates was used as one valid sample data point. Each group have more than 3 biological replicates, and each data point in the figures represents one independent biological sample. To compare mechanical pain thresholds among different groups at various time points, we applied a two-way repeated-measure ANOVA. Differences between two groups were evaluated using an unpaired t-test, while distinctions among multiple groups were evaluated via one-way ANOVA, followed by Dunnett’s *t*-test for post hoc comparisons. A significance level of *P* < 0.05 was considered statistically significant.

## Results

### Microglial LAPF participates in the progression of HSV-1-induced neuroinflammatory pain

**Fig. 1:**
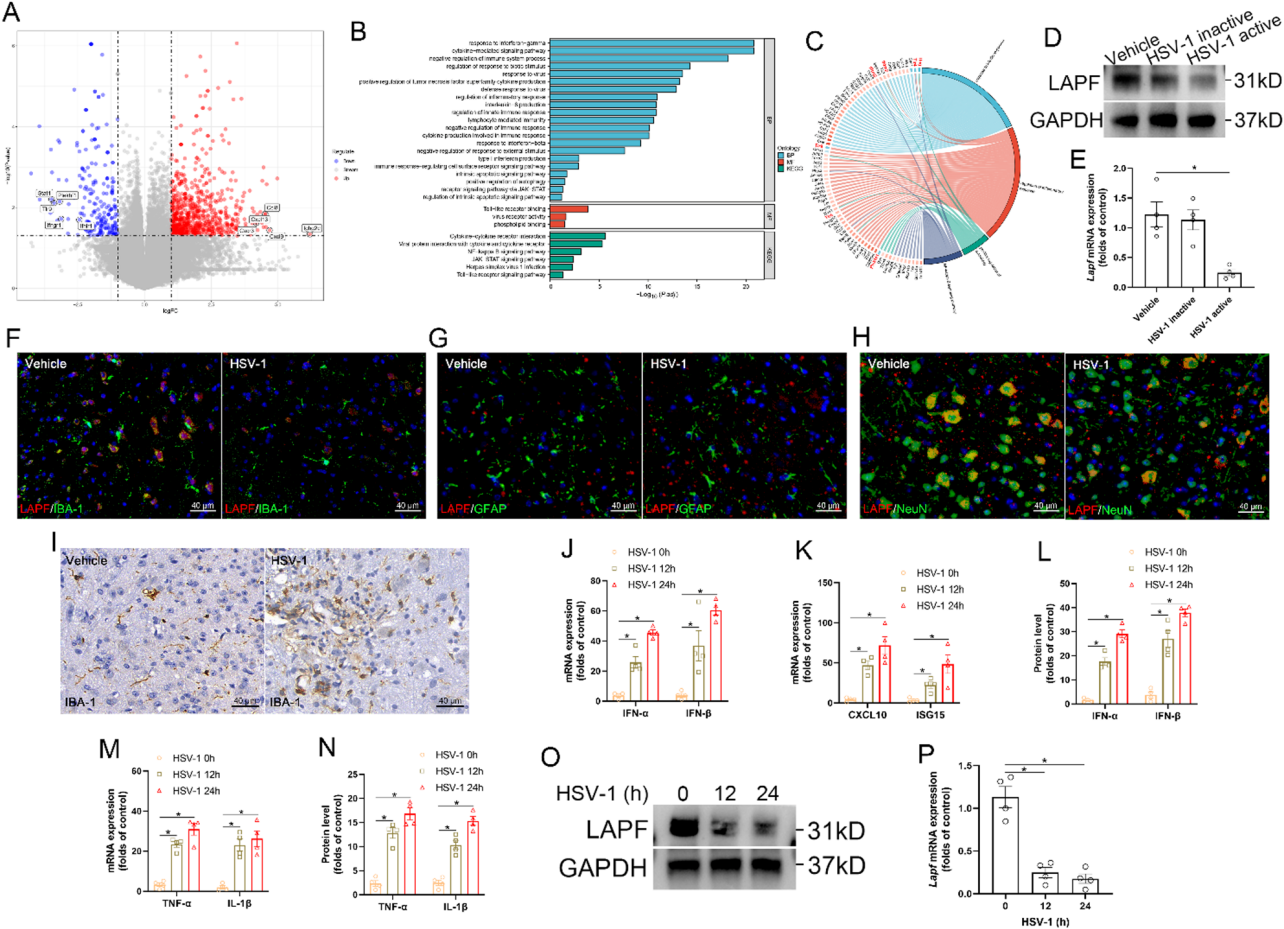
Microglial LAPF participates in the progression of HSV-1-induced neuroinflammatory pain.** A** Volcano plot of HSV-1 infected and control mice. **B** GO terms and KEGG enrichment of the DEGs. **C** The chord diagram reflecting key genes and pathways. **D**,** E** The protein production (**D**) and transcription (**E**) of LAPF in spinal dorsal horn after HSV-1 infection. **F-H** Co-expression of LAPF and IBA-1 (**F**), GFAP (**G**), and NeuN (**H**) in spinal dorsal horn after HSV-1 infection. **I** Microglial activation in spinal dorsal horn after HSV-1 infection. **J**,** K** Transcriptions of IFN-I (**J**) and ISGs (**K**) in BV-2 cells after HSV-1 infection. **L** Protein level of IFN-I in BV-2 cells after HSV-1 infection. **M** Transcriptions of inflammatory cytokines in BV-2 cells after HSV-1 infection. **N** Protein level of inflammatory cytokines in BV-2 cells after HSV-1 infection. **O**,** P** The protein production (**O**) and transcription (**P**) of LAPF in BV-2 cells after HSV-1 infection. mRNA expression. (Fold changes were calculated relative to the control sample, *n* = 4, ^*^P < 0.05)

This study extends our earlier investigations and is based on a well-established viral infection-induced neuropathic pain model, wherein all animal models used herein were stably induced by HSV‑1 infection as previously characterized [11]. To explore the regulatory mechanism of antiviral innate immunity in HSV-1-induced neuroinflammatory pain, we firstly conducted mRNA microarray analysis of spinal dorsal horns from model mice and control mice. The volcano plot revealed DEGs including upregulated and downregulated genes between model and control mice (Fig. 1A). GO and KEGG enrichment analysis demonstrated that the DEGs were primarily associated with antiviral innate immunity, inflammatory response, interferon production, Toll-like receptor signaling pathway, and response to virus (Fig. 1B). Based on enrichment analysis, we utilized the provided log-fold change (log FC) of molecules to calculate the z-score for each entry, thereby determining whether the corresponding genes and entries were positively or negatively regulated. The chord diagram identified several key pivotal genes related to these pathways, including Plekhf1 (*Lapf*), *Tlr6*, *Tlr9*, *Ifitm3*, *Ifitm2*, *Tlr4*, and *Ifng* (Fig. 1C). Furthermore, we confirmed the decreased protein and transcription level of LAPF in the spinal dorsal horn of mice following HSV-1 infection (Fig. 1D, E). Additionally, HSV-1 infection decreased the co-expression of LAPF and microglia marker IBA-1, while no changes were observed in the co-expression of LAPF and neuron marker NeuN. Co-expression of LAPF and astrocyte marker GFAP was not observed (Fig. 1F-H). Furthermore, increased microglia activation was observed in the spinal dorsal horn after HSV-1 infection (Fig. 1I), suggesting that microglia were the effector cells through which LAPF regulated antiviral innate immunity. Subsequently, we infected cultured BV-2 microglia cells with HSV-1 and confirmed the elevated transcriptions of IFN-I (Fig. 1J), ISGs (Fig. 1K), and inflammatory cytokines (Fig. 1M), accompanied with elevated protein production of IFN-I (Fig. 1L) and inflammatory cytokines (Fig. 1N). Also, protein and transcription level of LAPF in BV-2 cells were significantly decreased after HSV-1 infection (Fig. 1O, P), suggesting that microglial LAPF participated in the progression of HSV-1-induced neuroinflammatory pain.

### *Lapf* microglia-specific deficiency aggravates neuroinflammation and pain allodynia after HSV-1 infection by impairing antiviral innate immunity both *in vivo* and *in vitro*

**Fig. 2:**
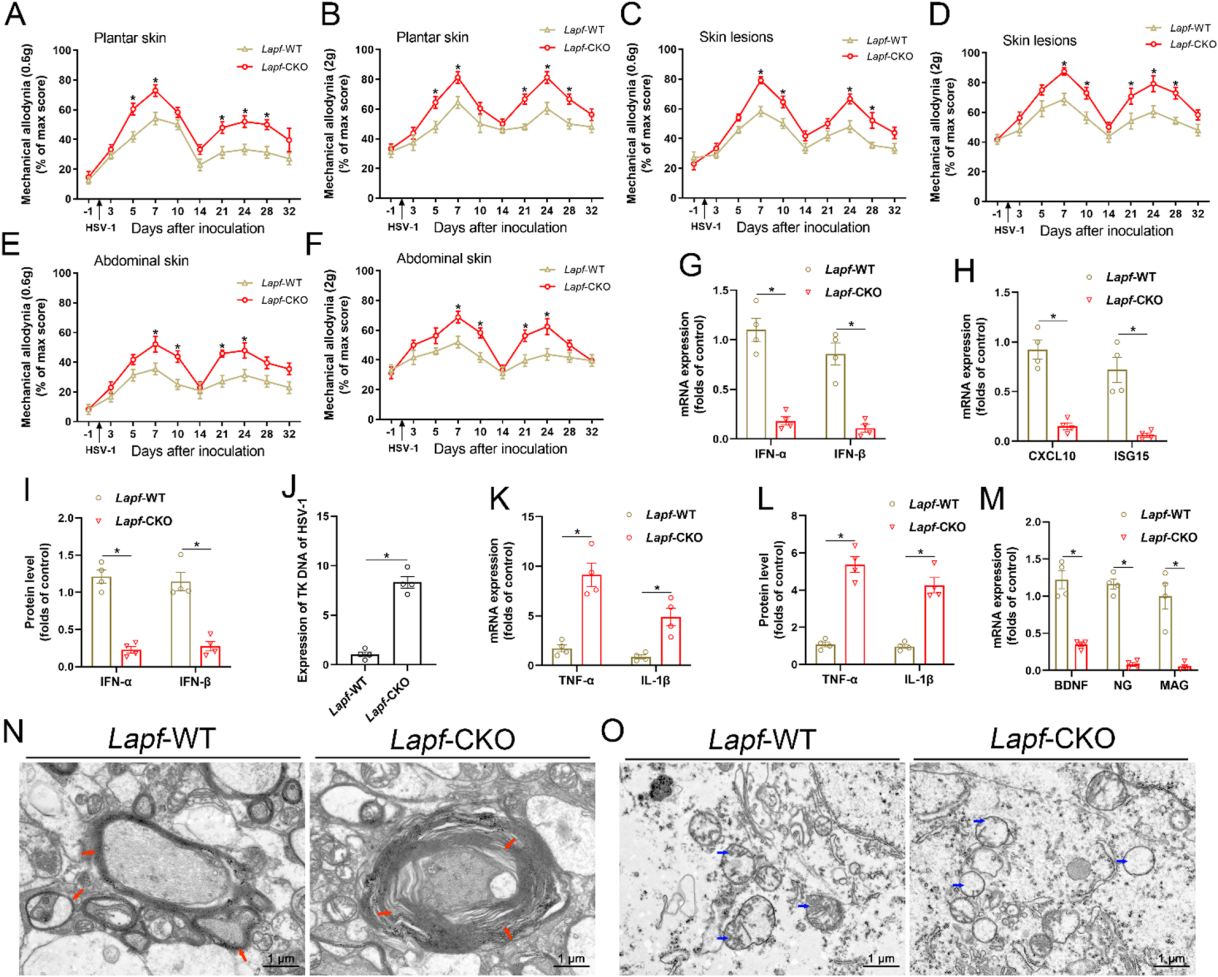
*Lapf*microglia-specific deficiency aggravates neuroinflammation and pain allodynia after HSV-1 infection by impairing antiviral innate immunity*in vivo*. **A**,** B** Mechanical pain allodynia in plantar skin. **C**,** D** Mechanical pain allodynia in skin lesions. **E**,** F** Mechanical pain allodynia in abdominal skin. **G**,** H** Transcriptions of IFN-I (**G**) and ISGs (**H**) in spinal dorsal horn of *Lapf*-CKO and *Lapf*-WT mice with HSV-1 infection. **I** Protein level of IFN-I in spinal dorsal horn of *Lapf*-CKO and *Lapf*-WT mice with HSV-1 infection. **J** Expression of TK DNA of HSV-1 in spinal dorsal horn of *Lapf*-CKO and *Lapf*-WT mice with HSV-1 infection. **K** Transcriptions of inflammatory cytokines in spinal dorsal horn of *Lapf*-CKO and *Lapf*-WT mice with HSV-1 infection. **L** Protein level of inflammatory cytokines in spinal dorsal horn of *Lapf*-CKO and *Lapf*-WT mice with HSV-1 infection. **M** Transcriptions of neuroprotective factors in spinal dorsal horn of *Lapf*-CKO and *Lapf*-WT mice with HSV-1 infection. **N**,** O** Transmission electron microscopy showing demyelination with red arrows (**N**) and mitochondrial edema with blue arrows (**O**) in spinal dorsal horn of *Lapf*-CKO and *Lapf*-WT mice with HSV-1 infection. (Fold changes were calculated relative to the control sample, *n* = 4, ^*^P < 0.05)

To investigate the role of LAPF in antiviral innate immunity and HSV-1-induced neuroinflammatory pain in vivo, we generated *Lapf* microglia-specific knockout mice (*Lapf*-CKO) and littermate control mice (*Lapf* -WT) using the Cre-LoxP system. *Lapf* microglia-specific deficiency was confirmed through DNA gel electrophoresis (Fig. S1A). *Lapf*-CKO mice with HSV-1 infection exhibited aggravated mechanical pain allodynia in the plantar skin (Fig. 2A, B), skin lesions (Fig. 2C, D) and abdominal skin (Fig. 2E, F) compared to *Lapf*-WT mice. Moreover, *Lapf* microglia-specific deficiency significantly decreased the transcriptions of IFN-I (Fig. 2G), ISGs (Fig. 2H), and the protein production of IFN-I (Fig. 2I), leading to the increased expression of TK DNA of HSV-1 (Fig. 2J), and increased transcription and protein production of inflammatory cytokines (Fig. 2K, L). Neuroprotective factors were also significantly decreased in *Lapf*-CKO mice compared to *Lapf*-WT mice (Fig. 2M). Transmission electron microscopy revealed aggravated axon demyelination (red arrows, Fig. 2N) and mitochondrial edema (blue arrows, Fig. 2O) in the spinal dorsal horns of *Lapf*-CKO mice compared to *Lapf*-WT mice. These findings collectively demonstrated that *Lapf* microglia-specific deficiency aggravated neuroinflammation and mechanical allodynia by impairing antiviral innate immunity *in vivo*.

**Fig. 3:**
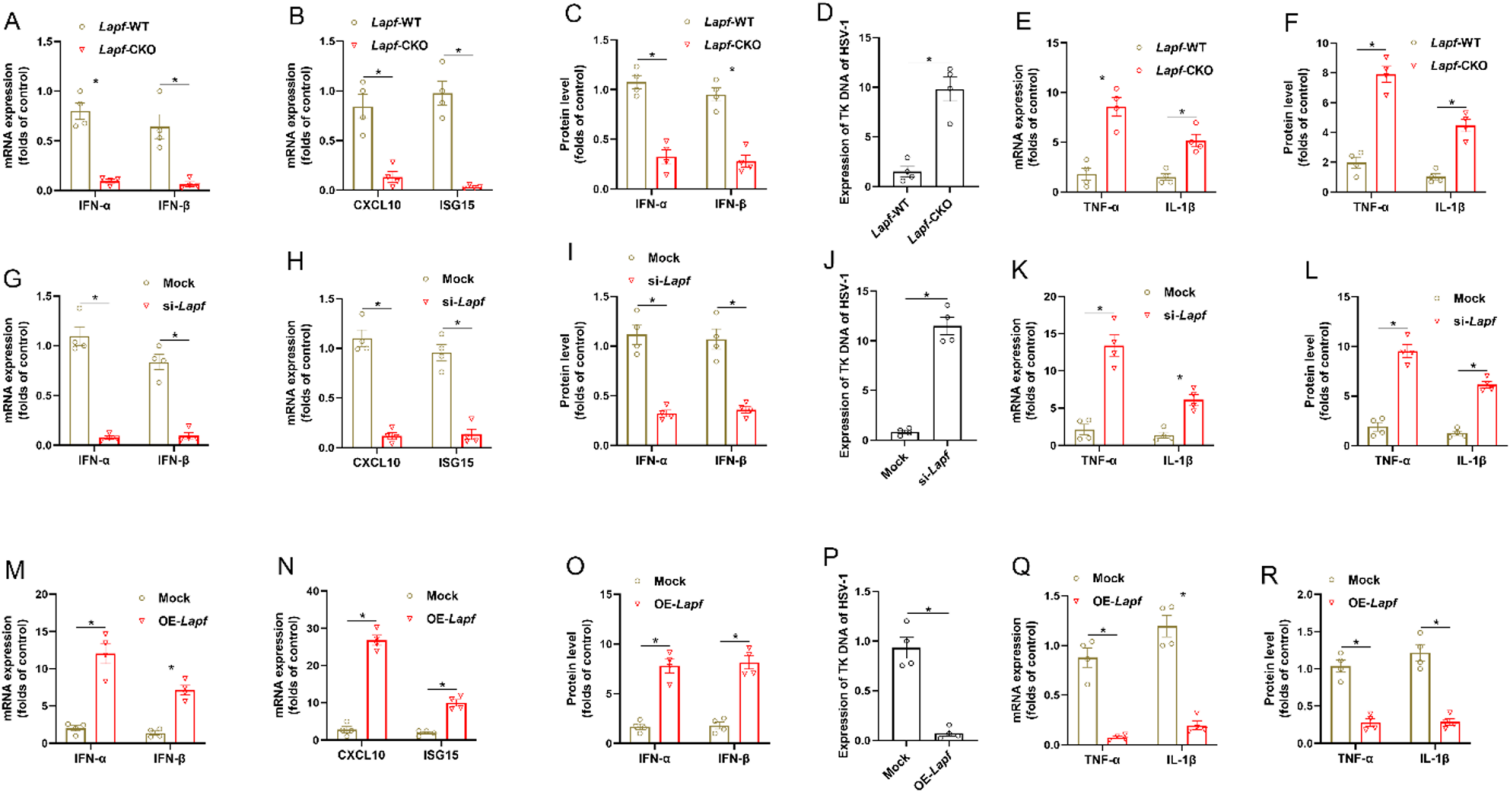
*Lapf*microglia-specific deficiency aggravates neuroinflammation by impairing antiviral innate immunity*in vitro*. **A**,** B** Transcriptions of IFN-I (**A**) and ISGs (**B**) in *Lapf* deficient primary microglia after HSV-1 infection. **C** Protein level of IFN-I in *Lapf* deficient primary microglia after HSV-1 infection. **D** Expression of TK DNA of HSV-1 in *Lapf* deficient primary microglia after HSV-1 infection. **E** Transcriptions of inflammatory cytokines in *Lapf* deficient primary microglia after HSV-1 infection. **F** Protein level of inflammatory cytokines in *Lapf* deficient primary microglia after HSV-1 infection. **G**,** H** Transcriptions of IFN-I (**G**) and ISGs (**H**) in *Lapf* knocking down BV-2 cells after HSV-1 infection. **I** Protein level of IFN-I in *Lapf* knocking down BV-2 cells after HSV-1 infection. **J** Expression of TK DNA of HSV-1 in *Lapf* knocking down BV-2 cells after HSV-1 infection. **K** Transcriptions of inflammatory cytokines in *Lapf* knocking down BV-2 cells after HSV-1 infection. **L** Protein level of inflammatory cytokines in *Lapf* knocking down BV-2 cells after HSV-1 infection. **M**,** N** Transcriptions of IFN-I (**M**) and ISGs (**N**) in *Lapf* overexpressing BV-2 cells after HSV-1 infection. **O** Protein level of IFN-I in *Lapf* overexpressing BV-2 cells after HSV-1 infection. **P** Expression of TK DNA of HSV-1 in *Lapf* overexpressing BV-2 cells after HSV-1 infection. **Q** Transcriptions of inflammatory cytokines in *Lapf* overexpressing BV-2 cells after HSV-1 infection. **R** Protein level of inflammatory cytokines in *Lapf* overexpressing BV-2 cells after HSV-1 infection. (Fold changes were calculated relative to the control sample, *n* = 4, ^*^P < 0.05)

To validate the role of microglial LAPF in antiviral innate immunity, we isolated primary microglia from *Lapf*-CKO and *Lapf*-WT mice and cultured them. The *Lapf* knockout in primary microglia was confirmed by western blotting and Q-PCR (Fig. S1B, C). Upon HSV-1 infection, *Lapf* deficiency significantly decreased the transcriptions of IFN-I (Fig. 3A), ISGs (Fig. 3B), and the protein production of IFN-I (Fig. 3C), leading to the increased expression of TK DNA of HSV-1 (Fig. 3D), and increased transcription and protein production of inflammatory cytokines (Fig. 3E, F). Furthermore, we employed siRNA to knockdown the expression of *Lapf* in BV-2 microglia cells, and the knockdown efficiency was confirmed by western blotting and Q-PCR (Fig. S1D, E). Following HSV-1 infection, *Lapf* knockdown significantly decreased the transcriptions of IFN-I (Fig. 3G), ISGs (Fig. 3H), and the protein production of IFN-I (Fig. 3I), leading to the increased expression of TK DNA of HSV-1 (Fig. 3J), and increased transcription and protein production of inflammatory cytokines (Fig. 3K, L). In addition, we overexpressed *Lapf* in BV-2 cells with plasmid, and the overexpression efficiency was confirmed by western blotting and Q-PCR (Fig. S1F, G). Upon HSV-1 infection, *Lapf* overexpression significantly increased the transcriptions of IFN-I (Fig. 3M), ISGs (Fig. 3N), and the protein production of IFN-I (Fig. 3O), leading to the decreased expression of TK DNA of HSV-1 (Fig. 3P), and decreased transcription and protein production of inflammatory cytokines (Fig. 3Q, R). These results indicated that *Lapf* microglia-specific deficiency aggravated neuroinflammation by impairing antiviral innate immunity *in vitro*.

### LAPF strengthens microglial antiviral innate immunity by increasing lysosomal acidity and lysosomal membrane stability

**Fig. 4:**
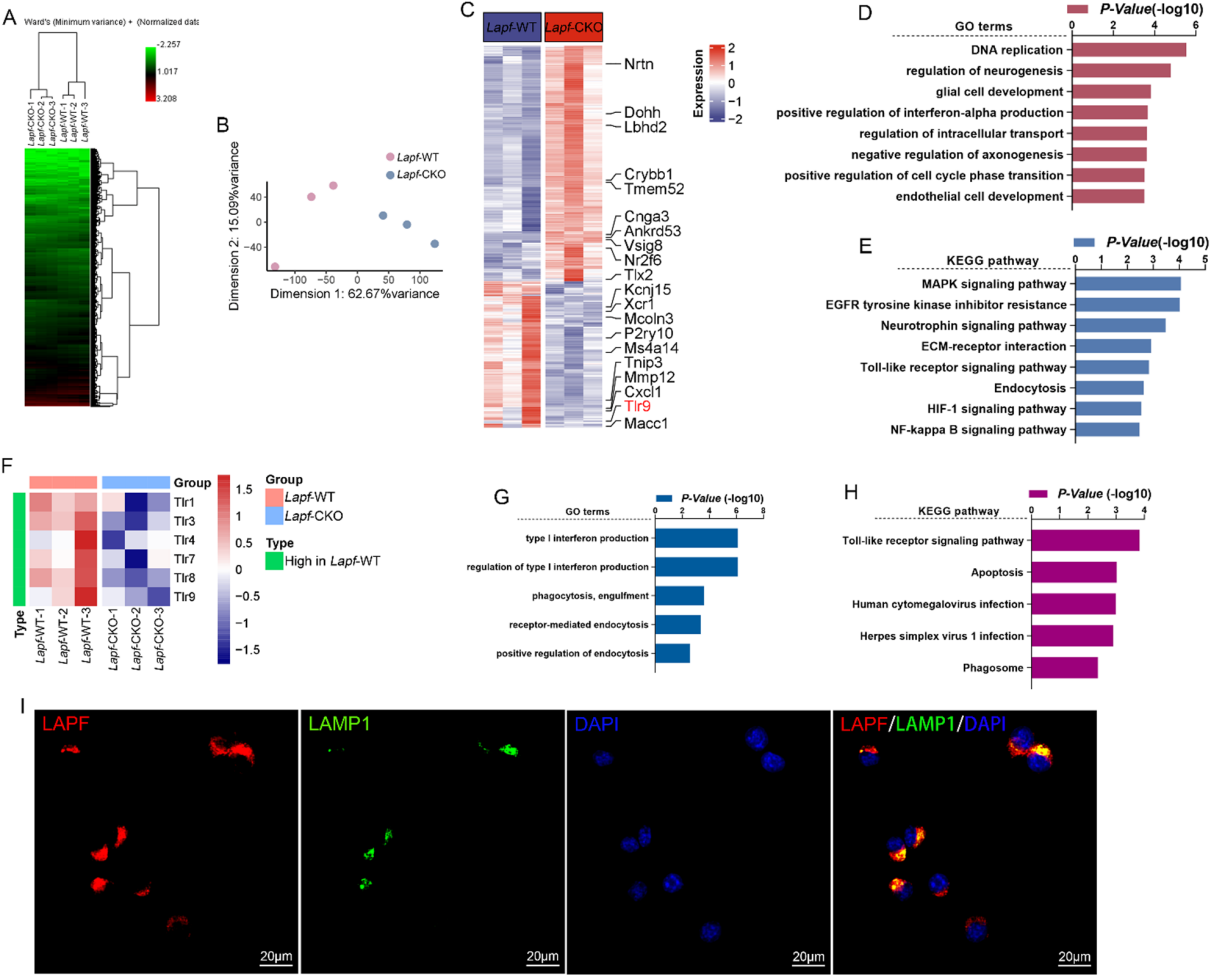
Identification of lysosome and TLR9 in LAPF strengthening microglial antiviral innate immunity.** A** HCA heatmap of DEGs in spinal dorsal horns from *Lapf*‑CKO and *Lapf*‑WT mice with HSV-1 infection (Euclidean distance, complete linkage). **B** PCA score plots showing distinct separation and high repeatability between *Lapf*‑CKO and *Lapf*‑WT groups. **C** Heatmap of several key pivotal genes between *Lapf*-CKO and *Lapf*-WT mice with HSV-1 infection. **D** GO enrichment analysis of all DEGs. **E** KEGG enrichment of all DEGs. **F** Heatmap of TLR family in DEGs. **G** GO enrichment of immune-related DEGs. **H** KEGG enrichment of immune-related DEGs. **I** Confocal revealing the Co-localization of LAPF and lysosomal marker LAMP1

**Fig. 5:**
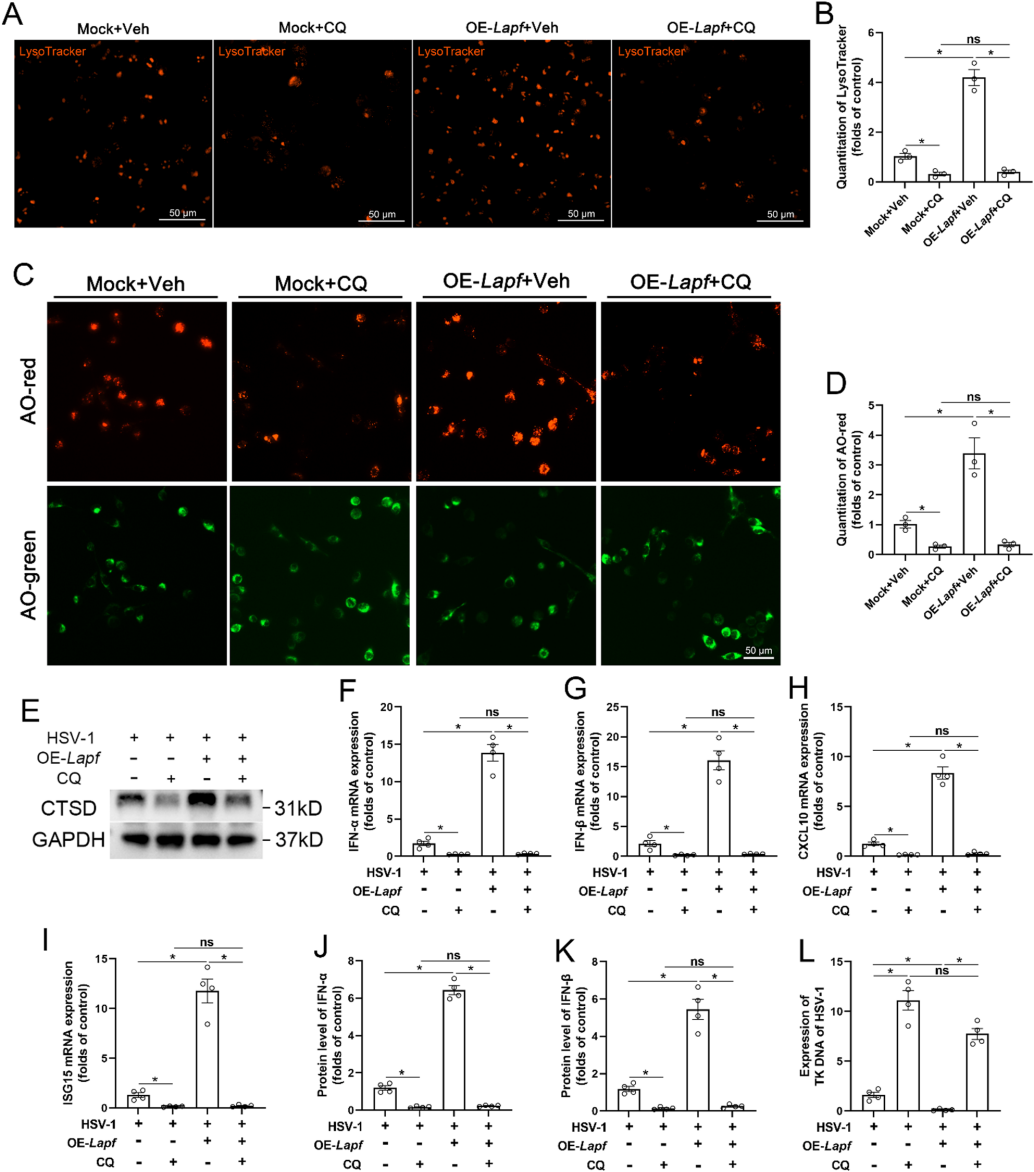
LAPF strengthens microglial antiviral innate immunity by increasing lysosomal acidity.** A** Confocal revealing LysoTracker of BV-2 cells with CQ treatment. Three independent biological replicates were performed; three random fields were captured per sample, and representative images are shown. **B** Quantification of fluorescence intensity in LysoTracker. **C** Confocal revealing AO staining of BV-2 cells with CQ treatment. Three independent biological replicates were performed; three random fields were captured per sample, and representative images are shown. **D** Quantification of fluorescence intensity in AO staining. **E** The expression of CTSD of BV-2 cells with CQ treatment. **F-I** Transcriptions of IFN-α (**F**), IFN-*β* (**G**), CXCL10 (**H**), and ISG15 (**I**) in BV-2 cells with CQ treatment. **J**,** K** Protein level of IFN-*α* (**J**) and IFN-*β* (**K**) in BV-2 cells with CQ treatment. **L** Expression of TK DNA of HSV-1 in BV-2 cells with CQ treatment. (Fold changes were calculated relative to the control sample, *n* = 4, ^*^P < 0.05)

To further explore the mechanism by which LAPF regulates antiviral innate immunity, we conducted mRNA microarray analysis of spinal dorsal horns from *Lapf*-CKO and *Lapf*-WT mice with HSV-1 infection. HCA (Fig. 4A) and PCA (Fig. 4B) confirmed that samples within each group were highly reproducible and clearly separated between *Lapf*‑CKO and *Lapf*‑WT groups. PCA was performed using normalized transcript counts of the whole transcriptome without selection of highly variable transcripts. Heatmap revealed several key pivotal genes between *Lapf*-CKO and *Lapf*-WT mice (Fig. 4C). GO and KEGG enrichment analyses using all DEGs demonstrated significant enrichment in pathways including DNA replication, glial cell development, toll-like receptor signaling pathway, and endocytosis (Fig. 4D, E). Considering the crucial role of the TLR family in antiviral innate immunity and inflammatory responses, we analyzed TLR molecules among the DEGs separately. Heatmap revealed that *Lapf* microglia-specific deficiency significantly reduced the expression of TLR1, TLR3, TLR4, TLR7, TLR8, and TLR9 (Fig. 4F). Given that TLR9 is primarily located within lysosomes, it can specifically recognize viral DNA that has been engulfed and degraded by lysosomes, initiating the synthesis and secretion of IFN-I to enhance antiviral innate immunity. We then performed focused GO and KEGG enrichment analyses using only immune‑related DEGs (selected for antiviral immunity, IFN‑I production, and inflammation), which highlighted key pathways including IFN-I production, phagocytosis and engulfment, endocytosis, TLR signaling pathway, and apoptosis (Fig. 4G, H). These results all suggested that lysosome and TLR9-mediated antiviral innate immunity participated in LAPF regulating the progression of HSV-1-induced neuroinflammatory pain. Confocal revealed that LAPF co-localized with the lysosomal marker LAMP1, indicating that LAPF has a functional localization in lysosomes (Fig. 4I). Since the activation of IFN-I synthesis by TLR9 recognizing viral DNA in lysosomes is highly dependent on lysosomal acidity, we further explored whether LAPF influences TLR9-mediated antiviral innate immunity by regulating lysosomal acidity. LysoTracker Red revealed that overexpressing *Lapf* in BV-2 cells increased lysosomal acidity, which was abolished by lysosomal acidification inhibitor CQ treatment (Fig. 5A), which was further confirmed by quantification of fluorescence intensity (Fig. 5B). Under normal conditions, the lysosome is acidic. The weakly basic fluorescent dye AO can penetrate the lysosomal membrane and accumulate in lysosomes, where it binds to the aqueous hydrolases and emits orange-red fluorescence. When the integrity of the lysosomal membrane is compromised, the capacity to accumulate AO is also disrupted, resulting in a reduction or absence of the orange-red fluorescence. Simultaneously, the maturation level of the CTSD reflects lysosomal functional integrity, as higher levels of mature CTSD indicate better lysosomal maturation and greater capacity to hydrolyze viral DNA. Since lysosomal membrane stability can affect the release of viral genomes from lysosomes into the cytoplasm, we further explored the impact of LAPF on lysosomal membrane stability. AO staining and western blotting both revealed that overexpressing *Lapf* in BV-2 cells increased AO-red and the expression of CTSD, which was abolished by CQ treatment (Fig. 5C-E), suggesting that LAPF strengthened lysosomal membrane stability. Furthermore, overexpressing *Lapf* in BV-2 cells significantly increased the transcriptions of IFN-I (Fig. 5F, G), ISGs (Fig. 5H, I), and the protein production of IFN-I (Fig. 5J, K), leading to the decreased expression of TK DNA of HSV-1 (Fig. 5L). These changes were all reversed by CQ treatment (Fig. 5F-L). These results indicated that LAPF strengthened microglial antiviral innate immunity by increasing lysosomal acidity and lysosomal membrane stability.

### LAPF promotes lysosomal acidification-dependent antiviral immunity via TLR9 activation and the cGAS-STING pathway

**Fig. 6:**
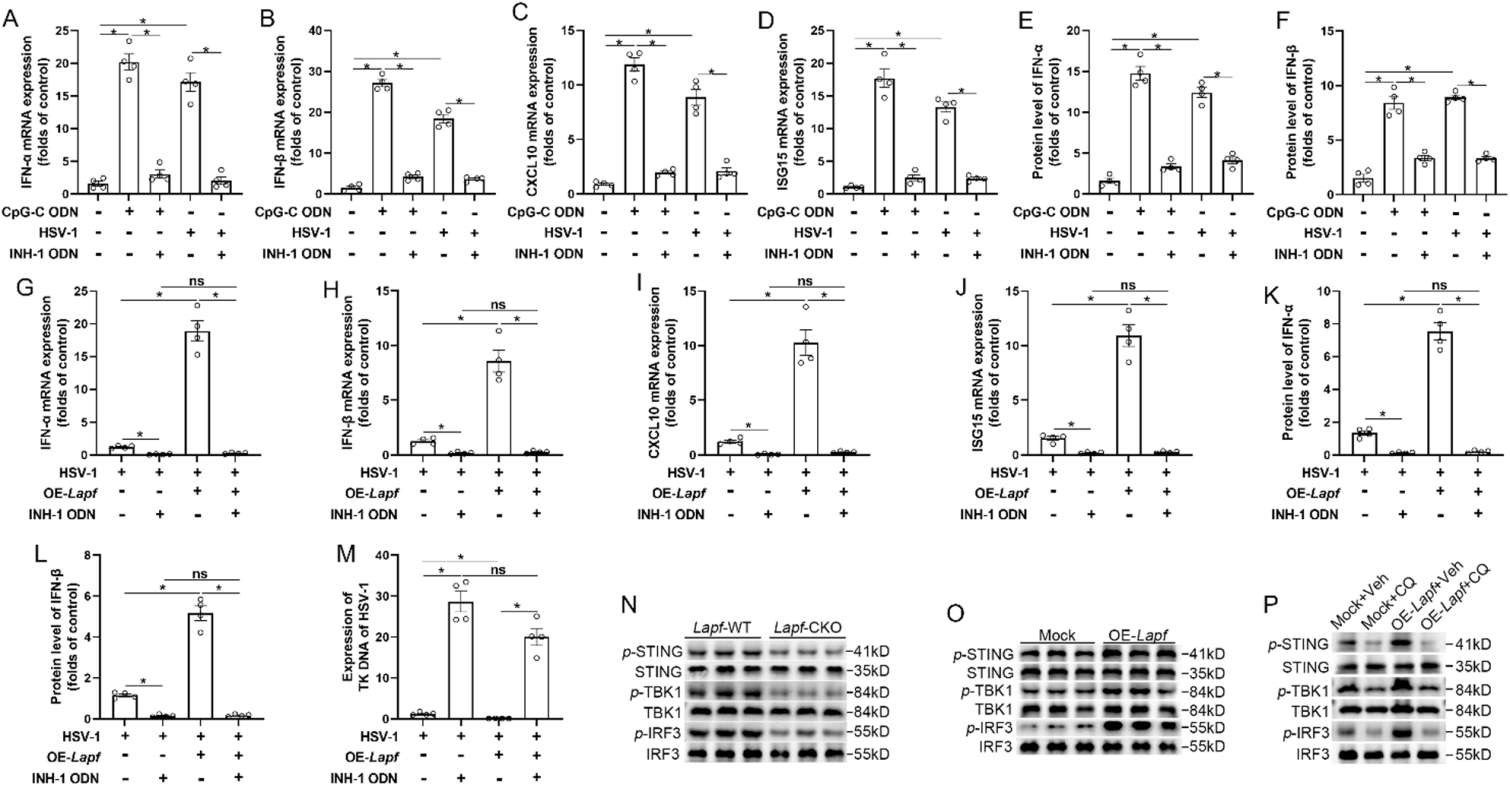
LAPF promotes lysosomal acidification-dependent antiviral immunity via TLR9 activation and the cGAS-STING pathway.** A-D** Transcriptions of IFN-*α* (**A**), IFN-*β* (**B**), CXCL10 (**C**), and ISG15 (**D**) in BV-2 cells with CpG-C ODN or INH-1 ODN treatment. **E**,** F** Protein level of IFN-*α* (**E**) and IFN-*β* (**F**) in BV-2 cells with CpG-C ODN or INH-1 ODN treatment. **G-J** Transcriptions of IFN-*α* (**G**), IFN-*β* (**H**), CXCL10 (**I**), and ISG15 (J) in BV-2 cells with *Lapf* overexpression or INH-1 ODN treatment. **K**,** L** Protein level of IFN-*α* (**K**) and IFN-*β* (**L**) in BV-2 cells with *Lapf* overexpression or INH-1 ODN treatment. **M** Expression of TK DNA of HSV-1 in BV-2 cells with *Lapf* overexpression or INH-1 ODN treatment. **N** Phosphorylation of STING, TBK1, and IRF3 in spinal dorsal horn of *Lapf*-CKO and *Lapf*-WT mice with HSV-1 infection. **O** Phosphorylation of STING, TBK1, and IRF3 in *Lapf* overexpressing BV-2 cells after HSV-1 infection. **P** Phosphorylation of STING, TBK1, and IRF3 in BV-2 cells with CQ treatment. (Fold changes were calculated relative to the control sample, *n* = 4, ^*^P < 0.05)

Lysosomes are capable of encapsulating and breaking down viruses into their individual components, a process that is highly dependent on the acidity of the lysosomes. In lysosomes, TLR9 undergo proteolytic processing by cathepsins and asparagine endopeptidase to become functionally competent receptors. After that, activated TLR9 can recognize, respectively, ssRNA or dsDNA and initiate IFN-I production, which can inhibit viral duplication and strengthen antiviral innate immunity. To further investigate whether TLR9 is involved in the LAPF-regulated antiviral innate immunity, we utilized TLR9 agonists and antagonists. Since TLR9 recognizes unmethylated CpG motifs of bacterial and viral ssDNA, we used CpG-C ODN, a synthetic TLR9 ligand containing unmethylated CpG sequences, as TLR9 agonist. INH-1 ODN, a TLR9 synthetic inhibitory oligonucleotide, was employed to inhibit TLR9 pathway. Both HSV-1 and CpG-C ODN treatment in BV-2 cells significantly increased the transcriptions of IFN-I (Fig. 6A, B), ISGs (Fig. 6C, D), and the protein production of IFN-I (Fig. 6E, F), which were all reversed by INH-1 treatment (Fig. 6A-F). To further explore the role of TLR9 activation in LAPF strengthening lysosomal acidity-mediated antiviral innate immunity, we overexpressed *Lapf* in BV-2 cells. Overexpressing *Lapf* significantly increased the transcriptions of IFN-I (Fig. 6G, H), ISGs (Fig. 6I, J), and the protein production of IFN-I (Fig. 6K, L), leading to the decreased expression of TK DNA of HSV-1 (Fig. 6M). These changes were all reversed by INH-1 treatment (Fig. 6G-M). In the presence of INH-1 treatment, the impact of *Lapf* overexpression on IFN-I and ISGs were abolished (Fig. 6G-M).

Given that the cGAS-STING-mediated IFN-I production represents a canonical antiviral pathway, we next examined whether LAPF influences the activation of the cGAS-STING pathway after HSV-1 infection. In *Lapf* microglia-specific deficient mice with HSV-1 infection, the phosphorylation levels of STING, TBK1, and IRF3 in the spinal dorsal horn were markedly reduced (Fig. 6N), accompanied by a significant increase in HSV-1 TK DNA expression (Fig. 2J). Conversely, overexpression of *Lapf* in BV-2 cells enhanced the phosphorylation of STING, TBK1, and IRF3 (Fig. 6O) and reduced HSV-1 TK DNA levels (Fig. 3P), indicating that LAPF potentiates cGAS-STING-dependent antiviral innate immunity. To determine whether this regulatory effect requires lysosomal acidification, we treated BV-2 cells with the lysosomal acidification inhibitor CQ. CQ markedly suppressed the phosphorylation of STING, TBK1, and IRF3 in control cells (Fig. 6P) and increased HSV-1 TK DNA abundance (Fig. 5L). Notably, under CQ treatment, *Lapf* overexpression no longer enhanced the phosphorylation of STING, TBK1, and IRF3 (Fig. 6P), nor did it reduce HSV-1 TK DNA levels (Fig. 5L), demonstrating that LAPF activates the cGAS-STING pathway in a lysosome-acidification-dependent manner. Together, these findings further support that LAPF promotes lysosomal acidification-dependent antiviral immunity via TLR9 activation and the cGAS-STING pathway.

### Dephosphorylation enzyme inhibitor SHP099 alleviates neuroinflammation and pain allodynia in HSV-1 infected mice

**Fig. 7:**
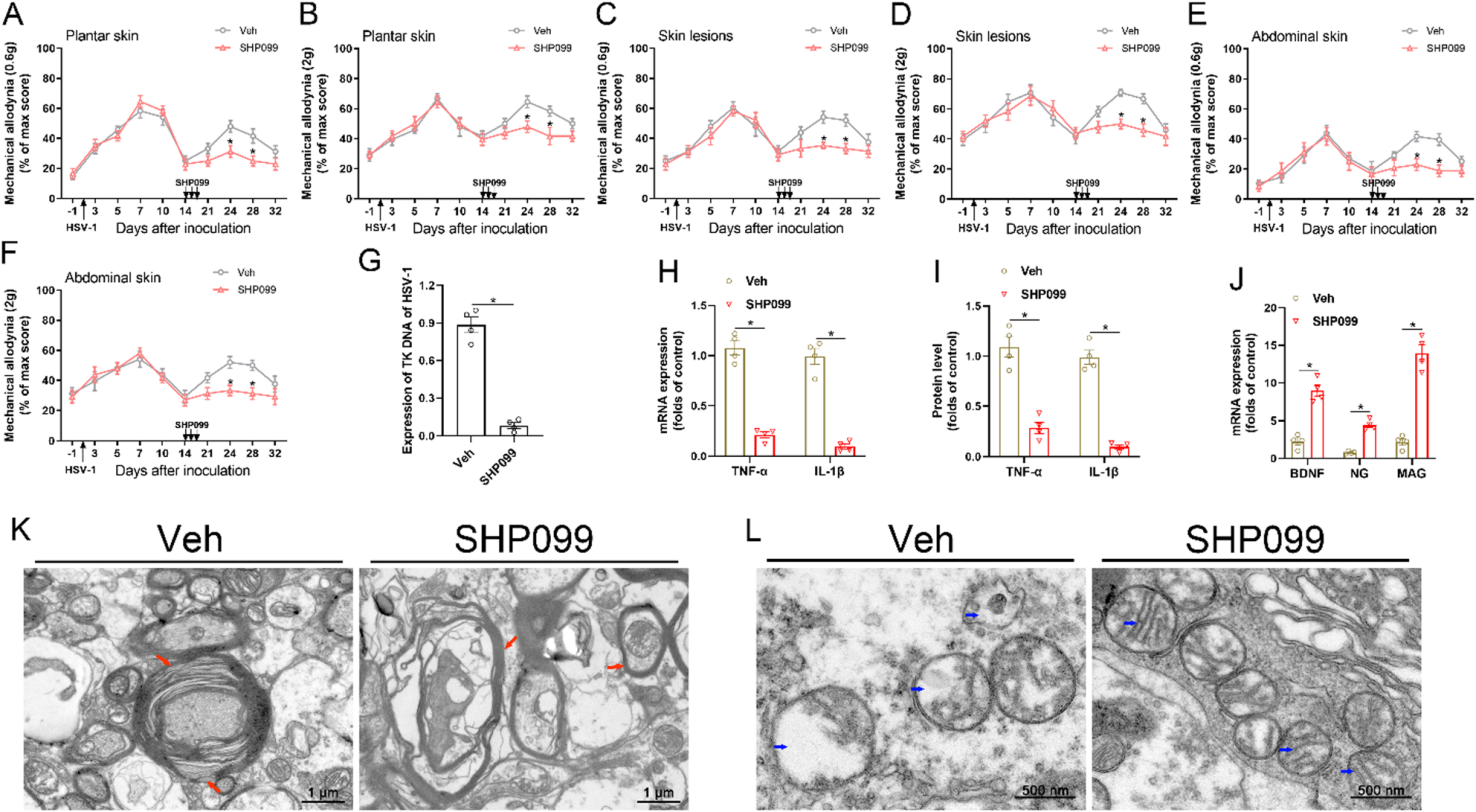
Dephosphorylation enzyme inhibitor SHP099 alleviates neuroinflammation and pain allodynia in HSV-1 infected mice.** A**,** B** Mechanical pain allodynia in plantar skin. **C**,** D** Mechanical pain allodynia in skin lesions. **E**,** F** Mechanical pain allodynia in abdominal skin. **G** Expression of TK DNA of HSV-1 in spinal dorsal horn of HSV-1 infected mice with SHP099 or vehicle intrathecally. **H** Transcriptions of inflammatory cytokines in spinal dorsal horn of HSV-1 infected mice with SHP099 or vehicle intrathecally. **I** Protein level of inflammatory cytokines in spinal dorsal horn of HSV-1 infected mice with SHP099 or vehicle intrathecally. **J** Transcriptions of neuroprotective factors in spinal dorsal horn of HSV-1 infected mice with SHP099 or vehicle intrathecally. **K**,** L** Transmission electron microscopy showing demyelination with red arrows (**K**) and mitochondrial edema with blue arrows (**L**) in spinal dorsal horn of HSV-1 infected mice with SHP099 or vehicle intrathecally. (Fold changes were calculated relative to the control sample, *n* = 4, ^*^P < 0.05)

The inhibitor of the tyrosine phosphatase SHP2, SHP099, can increase the phosphorylation of LAPF following virus infection, enhancing the functionality of LAPF [25]. To investigate the potential therapeutic application of dephosphorylation enzyme inhibitor SHP099, by targeting the increase in LAPF phosphorylation in HSV-1 infected mice, we administrated SHP099 intrathecally for three consecutive days on Day 14 after HSV-1 infection. We found that SHP099 intrathecally significantly alleviated mechanical pain allodynia in the plantar skin (Fig. 7A, B), skin lesions (Fig. 7C, D) and abdominal skin (Fig. 7E, F) on Day 24 and 28. By obtaining the spinal dorsal horn samples on Day 24, we also found that SHP099 intrathecally significantly decreased the expression of TK DNA of HSV-1 (Fig. 7G), leading to the decreased transcription and protein production of inflammatory cytokines (Fig. 7H, I). Moreover, neuroprotective factors were also significantly increased in SHP099 intrathecally mice compared to vehicle intrathecally mice (Fig. 7J). Transmission electron microscopy revealed that SHP099 intrathecally also alleviated axon demyelination (red arrows, Fig. 7K) and mitochondrial edema (blue arrows, Fig. 7L) in the spinal dorsal horns of HSV-1 infected mice. These findings collectively demonstrated that SHP099, by targeting to increase LAPF phosphorylation, alleviated neuroinflammation and pain allodynia in HSV-1 infected mice.

## Discussion

Microglia, as innate immune cells in the nervous system, can eliminate viruses through lysosomal phagocytosis and induction of antiviral innate immune responses [26]. However, the role of microglial LAPF in the pathogenesis of virus-induced neuroinflammatory pain, particularly its mechanism in modulating lysosome-mediated antiviral innate immunity, remains elusive. By using an HSV-1-induced neuroinflammatory pain model, we found that HSV-1 infection significantly downregulates LAPF expression in spinal microglia, resulting in impaired lysosomal acidification and reduced membrane stability. This lysosomal dysfunction attenuates TLR9 activation and IFN-I production while promoting leakage of viral DNA into the cytoplasm. Importantly, LAPF deficiency also suppresses cGAS-STING signaling, as evidenced by reduced phosphorylation of STING, TBK1, and IRF3, indicating that both endo-lysosomal and cytosolic antiviral pathways depend on intact LAPF-mediated lysosomal homeostasis. Collectively, these defects enhance viral replication, neuroinflammation, and neuronal injury, ultimately contributing to HSV-1-induced neuroinflammatory pain. Notably, pharmacological activation of LAPF using the dephosphorylation inhibitor SHP099 alleviated neuroinflammation and mechanical allodynia, supporting its therapeutic potential. Together, our findings identify LAPF as a key regulator that coordinates lysosomal and cytosolic antiviral immunity in microglia and provides mechanistic insight into virus-induced neuroinflammatory pain.

HSV-1-associated neuroinflammatory pain is a debilitating condition arising from viral infection, characterized by neural damage, neuroinflammation, and alterations in synaptic plasticity [27]. The pathogenesis of virus-induced neuropathic pain is multifactorial, involving alterations in ion channel activity, immune evasion, metabolic reprogramming, and inflammatory responses. Both neurons and immune cells exhibit abnormal sensitivity to external stimuli following HSV-1 infection, further complicating disease progression [28]. Animal models, particularly those based on HSV-1 infection, have been instrumental in advancing our understanding of virus-induced neuroinflammatory pain, as HSV-1 can induce persistent neuroinflammation and pain-like behaviours [22, 29]. Interestingly, HSV-1 infection can still lead to sustained neuroinflammatory responses despite activation of antiviral innate immunity in the spinal dorsal horn, suggesting the presence of additional immune regulatory or escape mechanisms. Importantly, HSV-1 serves here as a robust experimental model for investigating virus-induced neuroinflammatory pain; however, extrapolation of these findings to other neurotropic viruses will require further investigation.

We also noted that mechanical allodynia was partially ameliorated around day 14 post HSV‑1 infection in both genotypes. This transient improvement coincides with the resolution of acute cutaneous lesions and partial viral containment in the early phase of infection. As skin inflammation and peripheral nociceptor sensitization subside by day 14, host innate immunity temporarily restricts viral replication and reduces acute neuroinflammation, leading to modest alleviation of pain hypersensitivity. Nevertheless, the pain does not fully resolve because HSV‑1 establishes latent infection in sensory ganglia and spinal dorsal horn, accompanied by sustained microglial activation and central sensitization. Notably, microglial LAPF deficiency blunts this partial recovery and maintains more severe allodynia, indicating that LAPF‑dependent lysosomal antiviral immunity is required for the transient attenuation of pain at this stage. This biphasic pain profile, acute severe allodynia followed by partial relief around day 14 and then persistent chronic pain, is consistent with the natural progression of HSV‑1‑induced PHN‑like pain in preclinical models, reflecting the transition from acute viral infection to latent infection‑driven chronic neuroinflammation.

Through transcriptomic sequencing of model mice and control mice, we identified LAPF as a potential regulator of antiviral innate immunity during disease progression. The relationship between LAPF and IFN-I-mediated antiviral innate immunity has garnered significant attention [18, 19]. LAPF has been found to impact the functionality of intracellular lysosomes, including their acidification and enzymatic activity. One of its key roles is facilitating the efficient engulfment and degradation of viral particles within lysosomes. This process enhances the cell’s ability to clear viruses effectively [16]. Furthermore, LAPF has been implicated in promoting the production of IFN-I. IFN-I represents a potent class of antiviral interferons capable of inhibiting viral replication and dissemination. Studies suggest that LAPF indirectly regulates the production of IFN-I by influencing lysosomal function, thereby strengthening the cell’s immune response against viruses [30, 31]. Our findings highlighted the pivotal role of LAPF in modulating antiviral innate immunity within the central nervous system during neuroinflammatory pain progression. Immunohistochemical analyses revealed that LAPF primarily governs these antiviral innate immune responses in microglia, aligning with their well-established role as immune cells within central nervous system. To further validate these findings, we employed conditional knockout mice of *Lapf* in microglia, and confirmed that *Lapf* deficiency weakened microglial antiviral innate immunity and exacerbated neuroinflammation, thereby promoting the progression of pian. Under normal immune conditions, microglia are proficient in generating sufficient levels of IFN-I to effectively inhibit HSV-1 replication [32]. However, the downregulation of LAPF induced by HSV-1 diminishes the antiviral immune capacity of microglia, resulting in reduced IFN-I production. This decrease in IFN-I levels proves insufficient to suppress HSV-1 replication, ultimately leading to the recurrence of pain. However, the precise mechanisms by which LAPF positively regulates antiviral innate immunity remain unidentified.

LAPF has a close functional relationship with lysosomes, especially in the context of pathogen defense [19]. Lysosomes are specialized cellular organelles with a pivotal role in the process of pathogen engulfment and degradation. In immune defense, lysosomes play a crucial role in the clearance of invading pathogens [33]. Upon pathogen recognition and engulfment, immune cells such as macrophages and neutrophils target the pathogen-containing phagosomes to fuse with lysosomes, forming a phagolysosome. Within the phagolysosome, lysosomal enzymes and acidic conditions facilitate the degradation of the pathogen, ensuring its effective elimination. This process is integral to the host’s innate immune response, enabling the removal of pathogens and the initiation of downstream immune signaling pathways [34]. In recent years, significant progress has been made in understanding the role of lysosomes in antiviral innate immunity, particularly within microglia in pathogen-mediated neuropathic pain [35]. One key advancement has been the recognition that lysosomal acidification plays a pivotal role in modulating the innate immune response against viral infections. Upon viral entry into microglia, lysosomes can efficiently engulf and degrade viral particles through a process known as lysosomal phagocytosis. This process not only aids in the physical clearance of viral components but also sets the stage for a cascade of immune responses. Upon phagocytosis, lysosomal acidification increases, which is essential for the activation of TLR9. TLR9 is a critical pattern recognition receptor that specifically recognizes viral DNA sequences within the lysosomal compartment. Upon activation, TLR9 initiates signaling pathways that culminate in the production of IFN-I, pivotal cytokines in the defense against viral infections. Notably, IFN-I can directly impede viral replication and assembly while also promoting the degradation of viral components [36, 37]. Our findings demonstrated that overexpressing *Lapf* in microglia led to an increase in lysosomal acidification, subsequently resulting in elevated IFN-I production. This process was highly dependent on the lysosomal pattern recognition receptor TLR9, as increased lysosomal acidification promotes TLR9 activation and enhances its ability to detect viral DNA within lysosomes, ultimately activating downstream IFN-I synthesis. Moreover, we also confirmed that LAPF could strengthen lysosomal membrane stability. The regulation of lysosomal membrane stability by LAPF emerges as a pivotal factor in antiviral immunity, underscoring a novel aspect of host defense mechanisms. These findings revealed that LAPF’s regulation on TLR9-mediated antiviral innate immunity was highly dependent on lysosomal acidification and lysosomal membrane stability, representing an innovative discovery of the regulatory mechanism of TLR9 on HSV-1-induced neuroinflammatory pain. The intrathecal application of SHP099, by targeting the increase in LAPF phosphorylation, also demonstrated significant efficacy in alleviating neuroinflammation and pain allodynia, underscoring the clinical translational value of targeting LAPF in the treatment of virus-induced neuroinflammatory pain.

We also confirmed that LAPF’s influence on antiviral immunity extends beyond endo-lysosomal TLR9 sensing to include modulation of the cytosolic cGAS-STING pathway. Specifically, microglia-specific *Lapf* deficiency reduced STING, TBK1 and IRF3 phosphorylation in the spinal dorsal horn, whereas LAPF overexpression enhanced phosphorylation of these effectors, correlating inversely with HSV-1 TK DNA abundance. Critically, inhibition of lysosomal acidification with CQ abrogated LAPF-dependent increases in STING-TBK1-IRF3 activation and reversed the antiviral benefit of *Lapf* overexpression. These findings support a model in which LAPF preserves lysosomal homeostasis, thereby enabling efficient TLR9-mediated nucleic acid sensing within lysosomes while preventing excessive cytosolic leakage or misrouting of viral DNA that could otherwise disrupt downstream cytosolic sensing and signaling pathways [38]. Mechanistically, preserved lysosomal acidity and membrane integrity may facilitate appropriate processing and clearance of viral debris and thereby govern the quantity, subcellular localization and trafficking of DNA that engage cGAS-STING [39]. Conversely, lysosomal dysfunction in *Lapf* deficiency could both blunt endosomal TLR9 activation and alter the cytosolic DNA landscape required for optimal STING signaling. Together, these data position LAPF as a nodal regulator that coordinates lysosomal homeostasis with parallel DNA-sensing pathways to mount a balanced, effective IFN-I response against neurotropic herpesvirus infection.

Although our study clearly demonstrates that HSV-1 infection downregulates LAPF expression in microglia, the precise molecular mechanism remains to be elucidated. We speculate that HSV-1 may suppress LAPF expression through multiple potential strategies, including virus-induced transcriptional repression or mRNA degradation, enhanced ubiquitin-proteasome or lysosome-dependent degradation, microRNA-mediated translational inhibition, or negative feedback loops triggered by excessive antiviral signaling. Future studies are warranted to dissect the detailed molecular events by which HSV-1 counteracts LAPF expression to facilitate immune evasion and pain progression.

In summary, HSV-1 infection reduces LAPF expression in microglia of the spinal dorsal horn, which compromises lysosomal acidification and attenuates TLR9 activation, thereby limiting the lysosomal sensing of viral DNA and decreasing IFN-I production. *Lapf* deficiency also destabilizes lysosomal membranes, allowing viral DNA to escape into the cytoplasm, where it amplifies and gains access to cytosolic sensors. Consistent with this, loss of LAPF markedly suppresses phosphorylation of STING, TBK1, and IRF3, demonstrating that cGAS-STING-mediated antiviral signaling is also impaired. The combined failure of TLR9 and cGAS-STING dependent pathways weakens microglial innate immunity, permitting uncontrolled HSV-1 replication that drives neuroinflammation, neuronal injury, and exacerbation of HSV-1-induced neuroinflammatory pain. Conversely, pharmacological enhancement of LAPF activity with the dephosphorylation inhibitor SHP099 alleviated neuroinflammation and mechanical allodynia, underscoring the therapeutic potential of targeting LAPF to restore lysosome-dependent antiviral immunity. These findings establish LAPF as a positive regulator of microglial antiviral responses by sustaining lysosomal function and coordinating both endo-lysosomal and cytosolic antiviral pathways, offering new mechanistic insight and a promising intervention strategy for virus-induced neuroinflammatory pain. We acknowledge that, while the current study provides mechanistic insight into HSV-1-induced neuroinflammatory pain, validation in other systems, including human cells or additional neurotropic viruses, represents an important future direction.

## Supplementary Information

Supplementary Material 1.

Supplementary Material 2.

Supplementary Material 3: Supplementary Fig. 1. (A) DNA gel electrophoresis to identify Lapf-WT and Lapf-CKO mice. (B, C) Efficiency of Lapf knockout in primary microglia by western blotting (B) and Q-PCR (C). (D, E) Efficiency of Lapf knockdown by western blotting (D) and Q-PCR (E). (F, G) Efficiency of Lapf overexpression by western blotting (F) and Q-PCR (G).

## Data Availability

The data that support this study are available from the corresponding author upon reasonable request.
