# Supplementary material for: LAPF enhances lysosomal acidification to promote TLR9 and cGAS-STING-mediated antiviral immunity and attenuate HSV-1-induced neuroinflammatory pain
Source: J Neuroinflammation. 2026 May 8;23:207. doi: 10.1186/s12974-026-03856-6 (PMC13285062; doi:10.1186/s12974-026-03856-6)

Fig. 1D

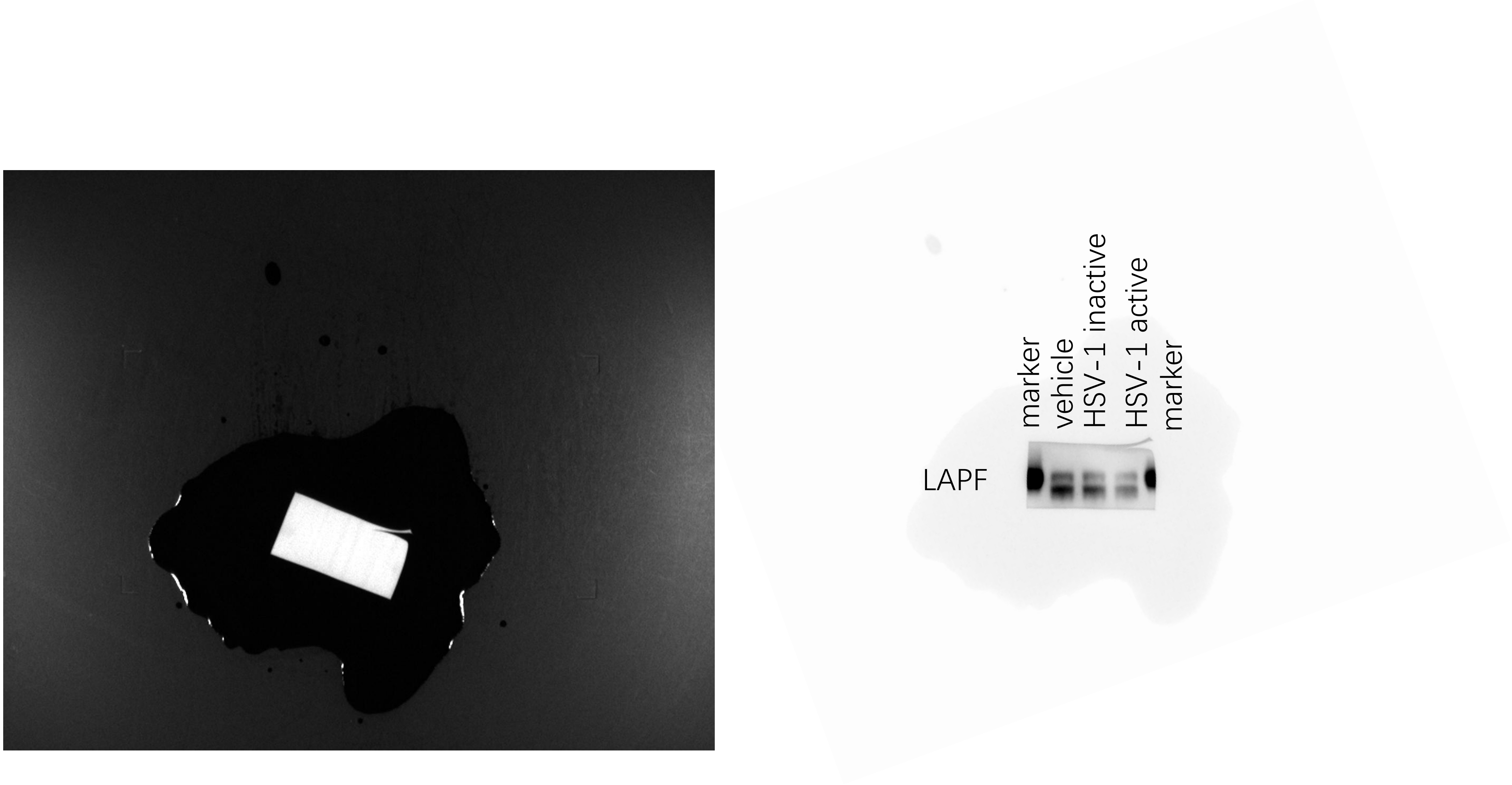

Fig. 1D

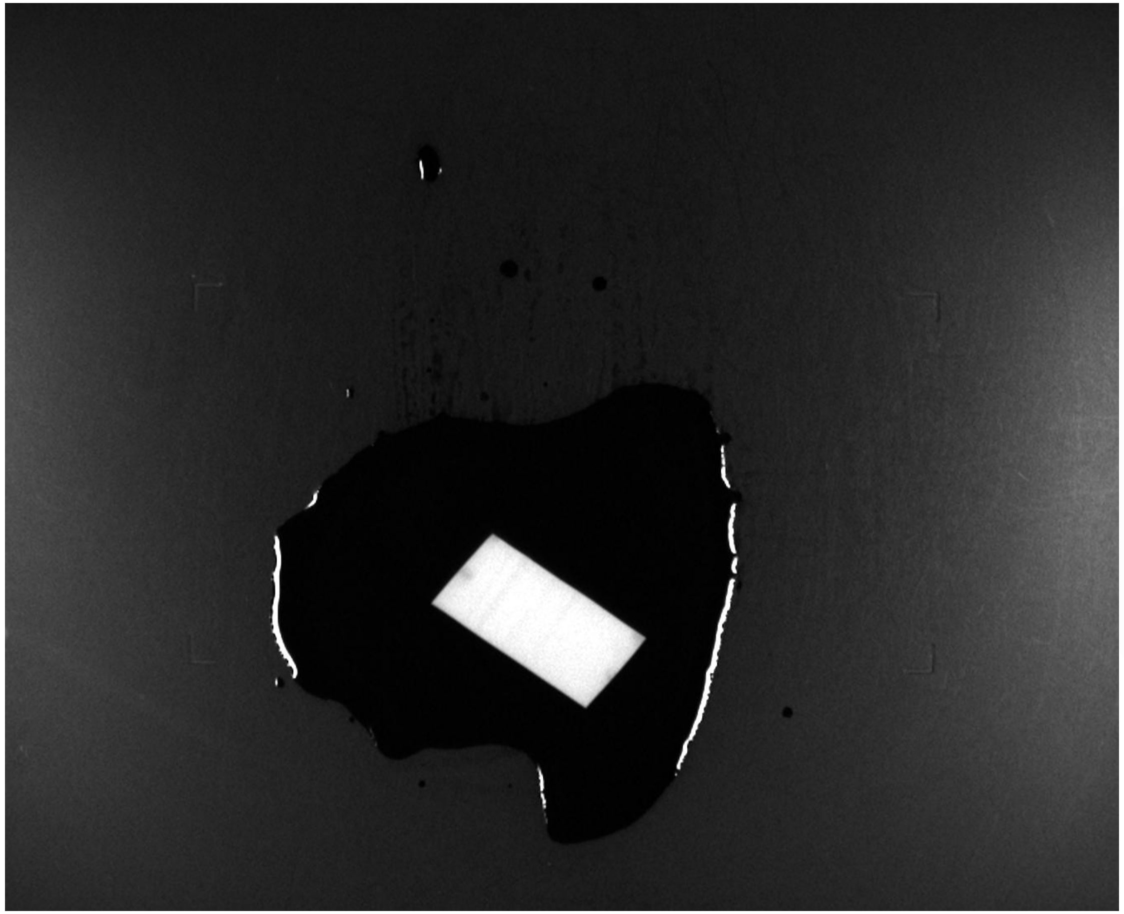

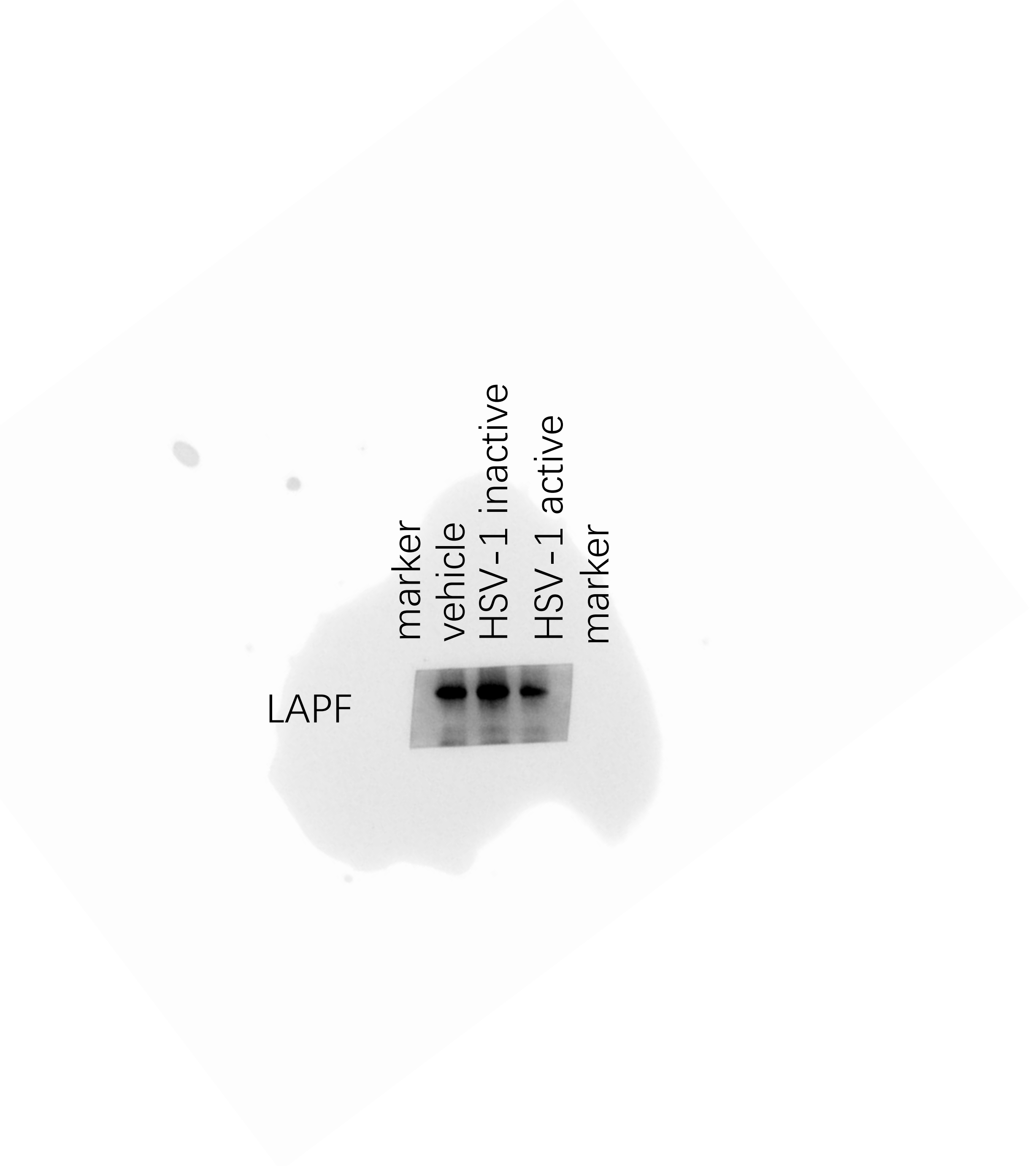

Fig. 1D

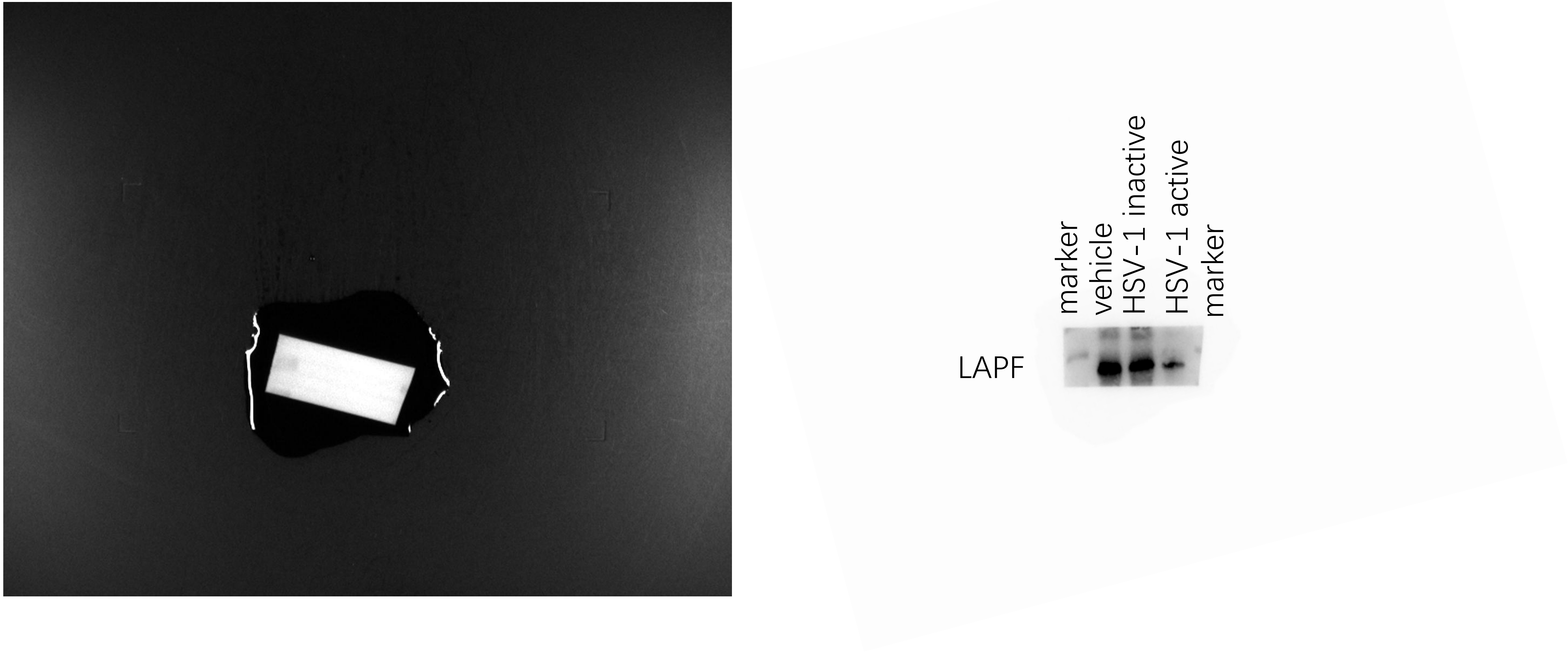

Fig. 1D

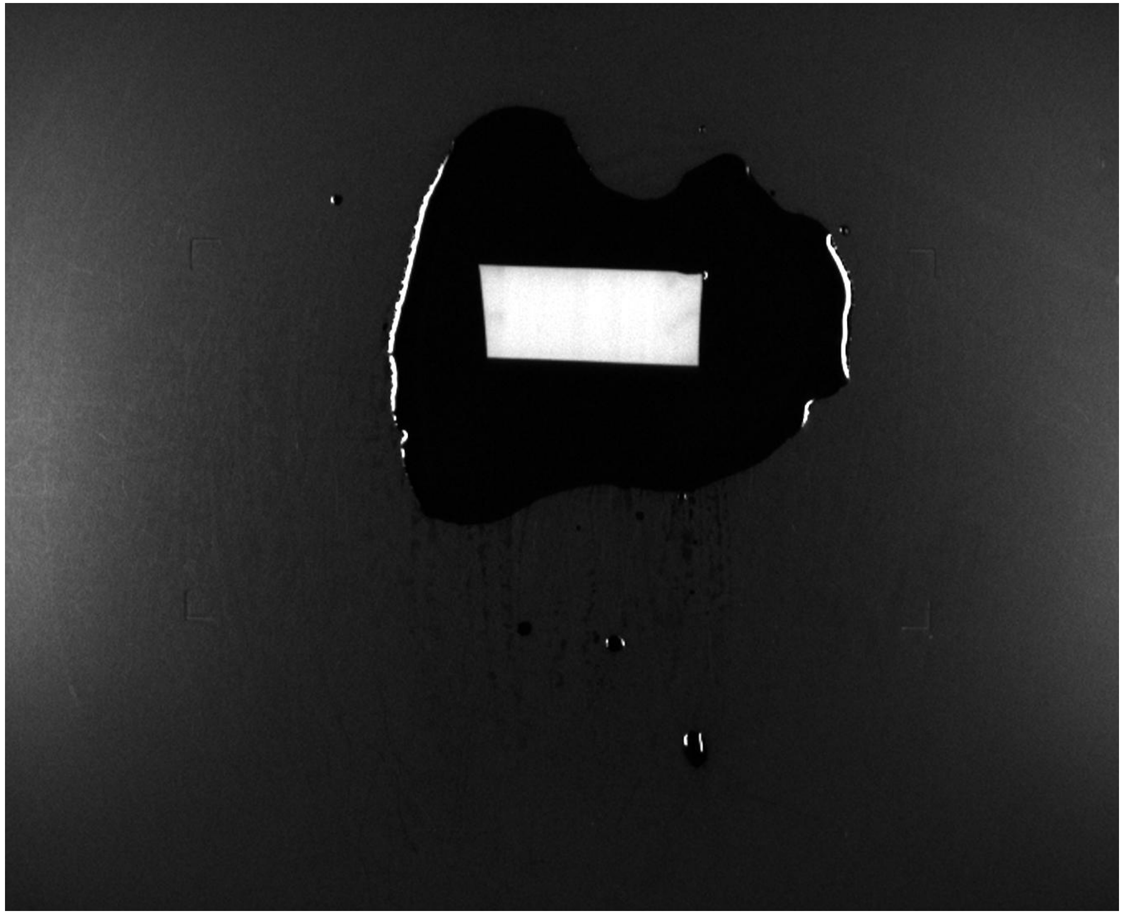

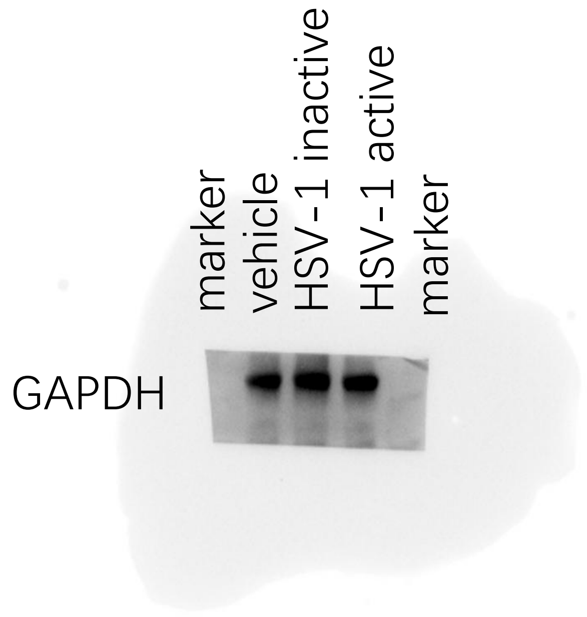

Fig. 1D

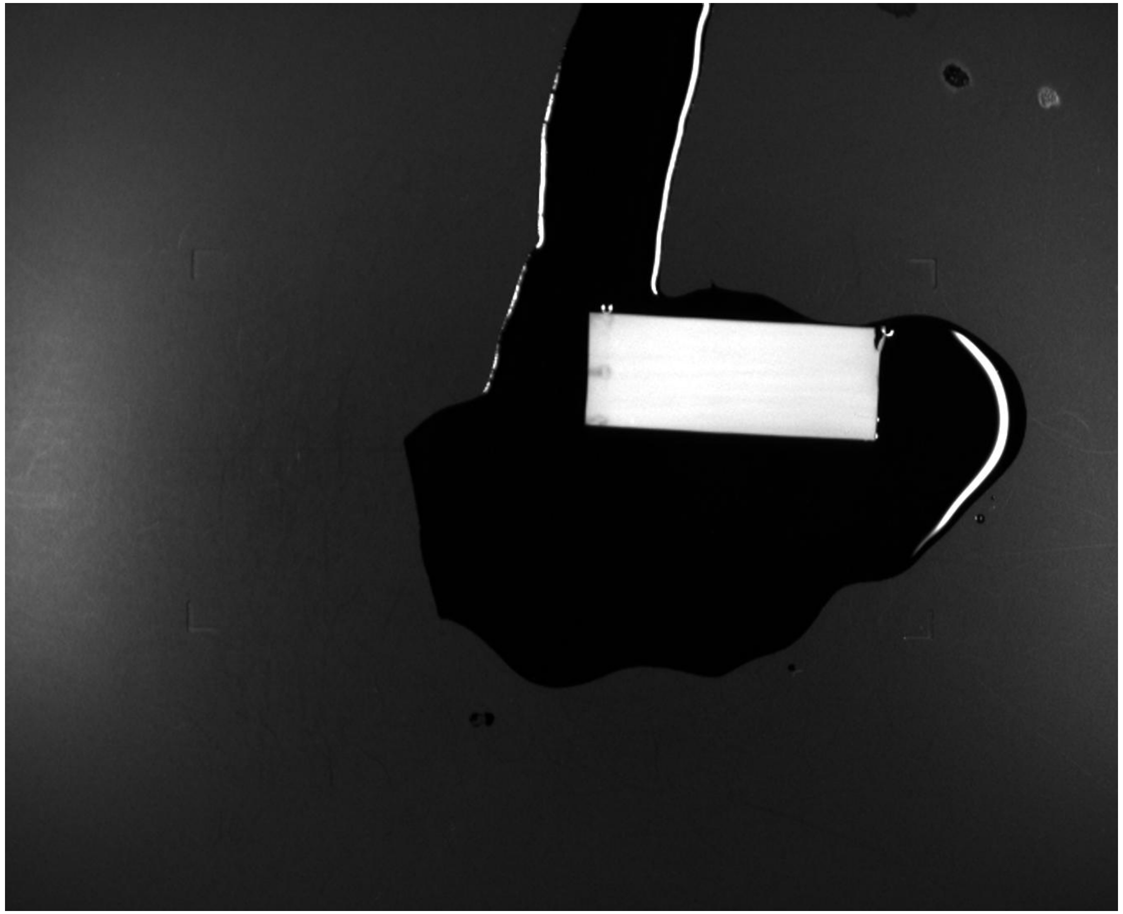

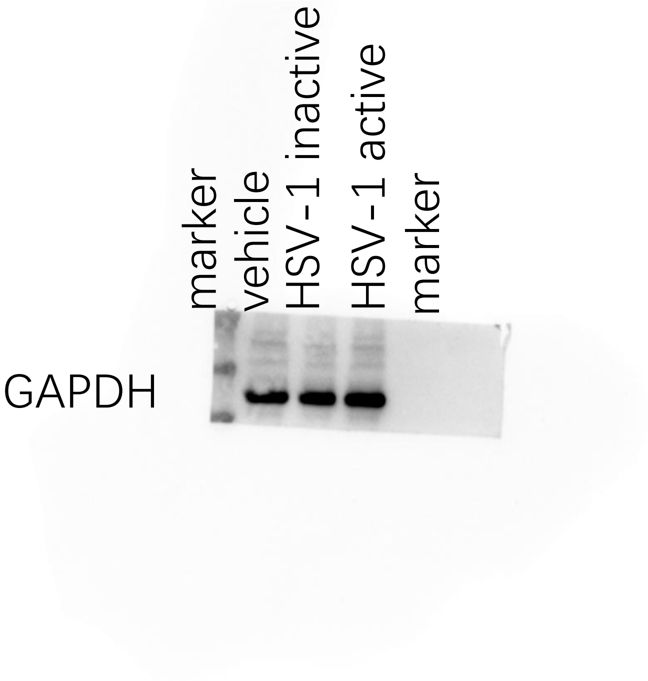

Fig. 1D

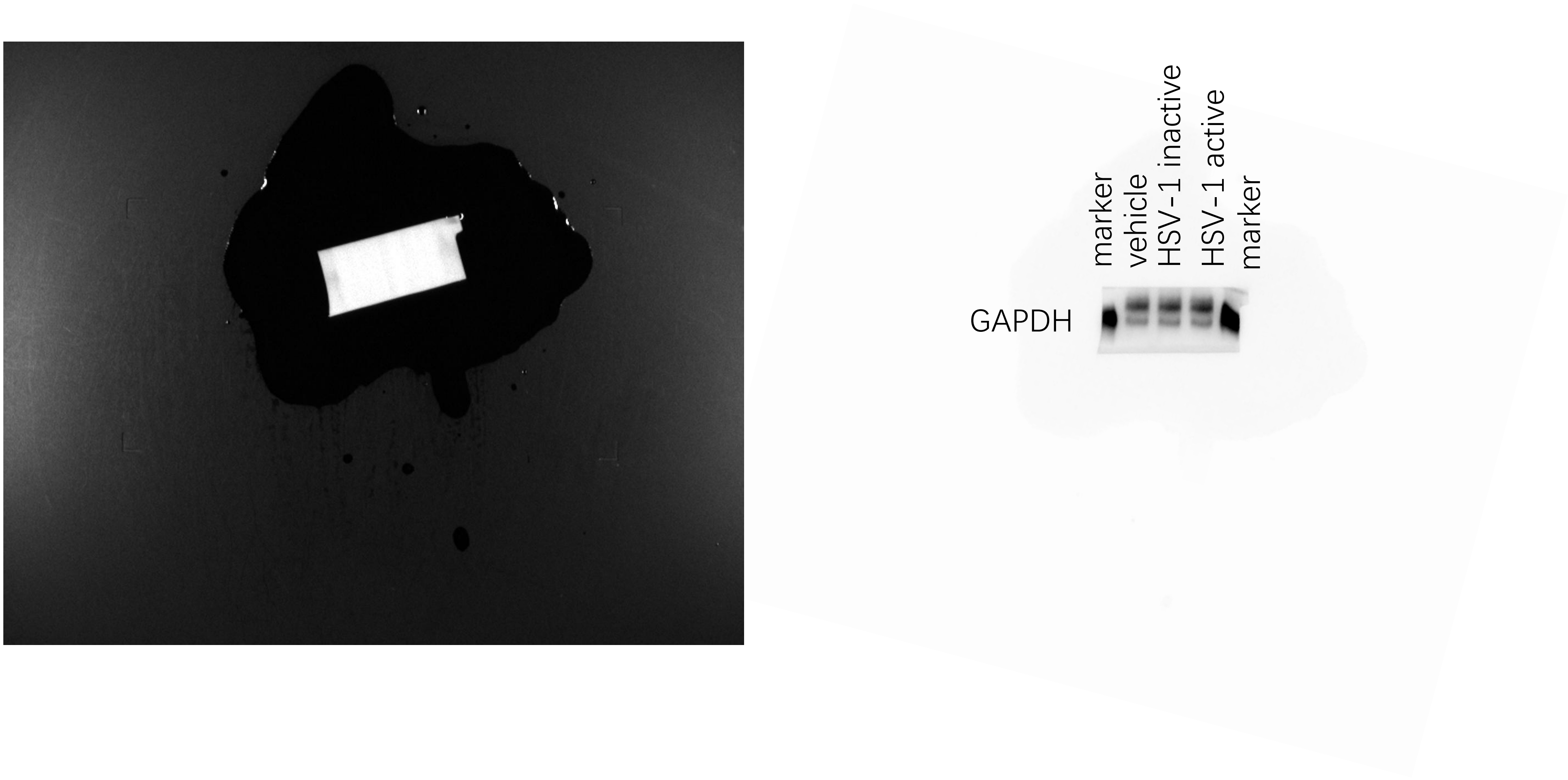

Fig. 1O

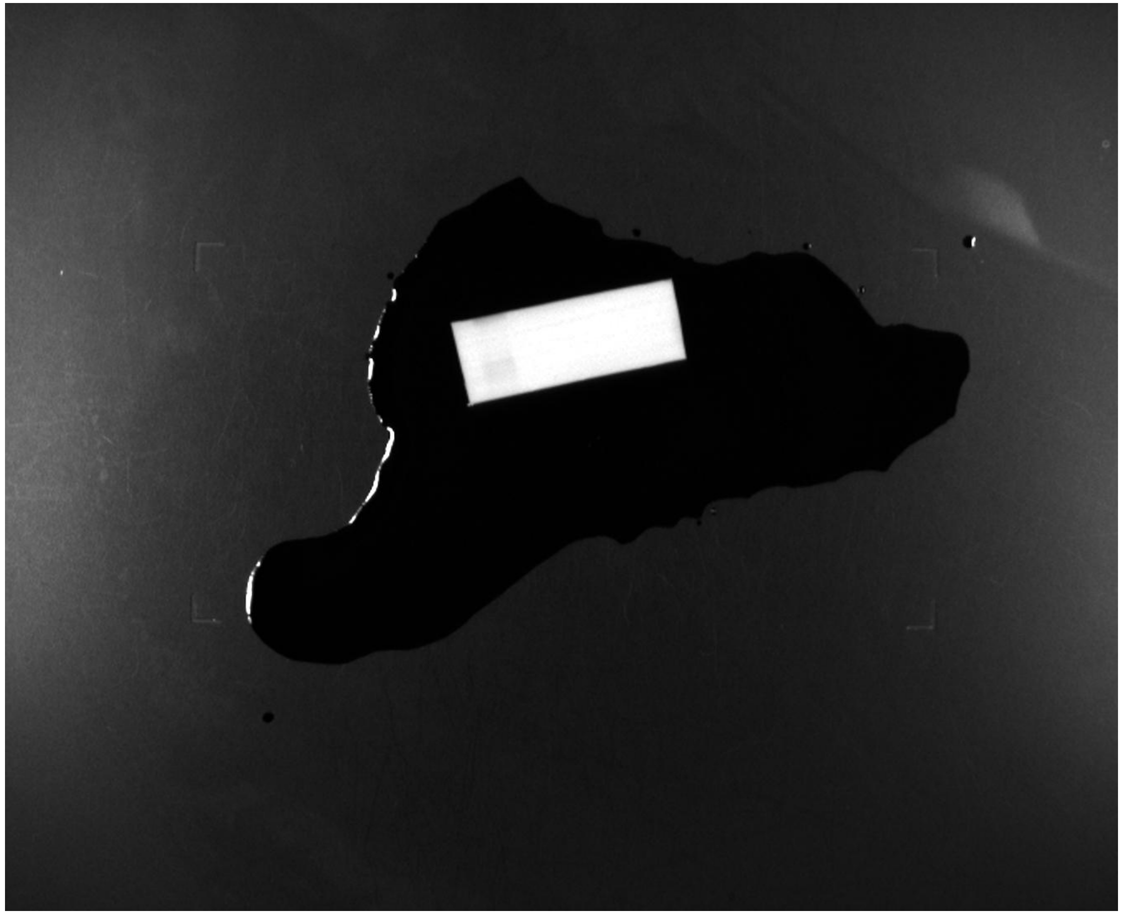

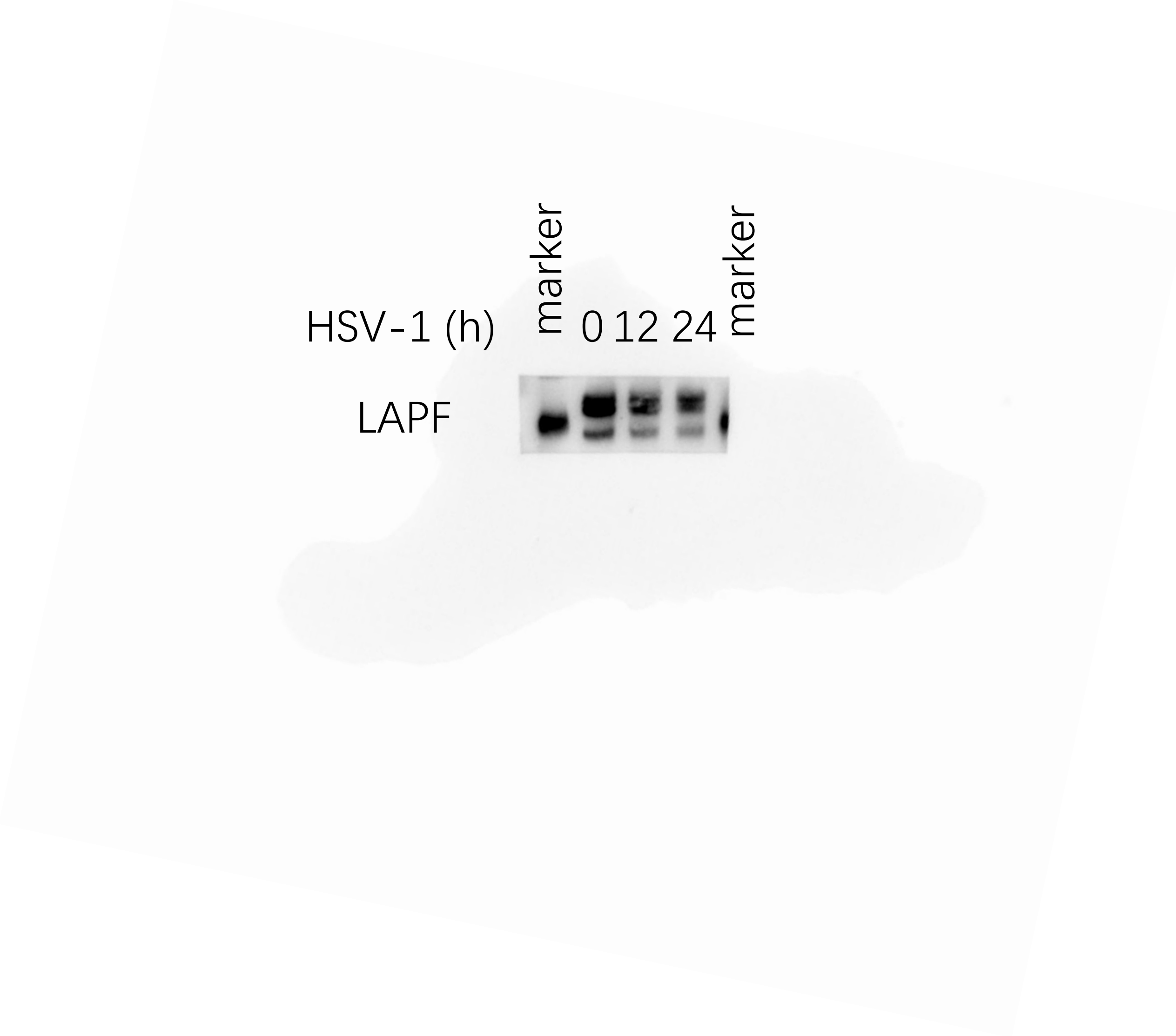

Fig. 1O

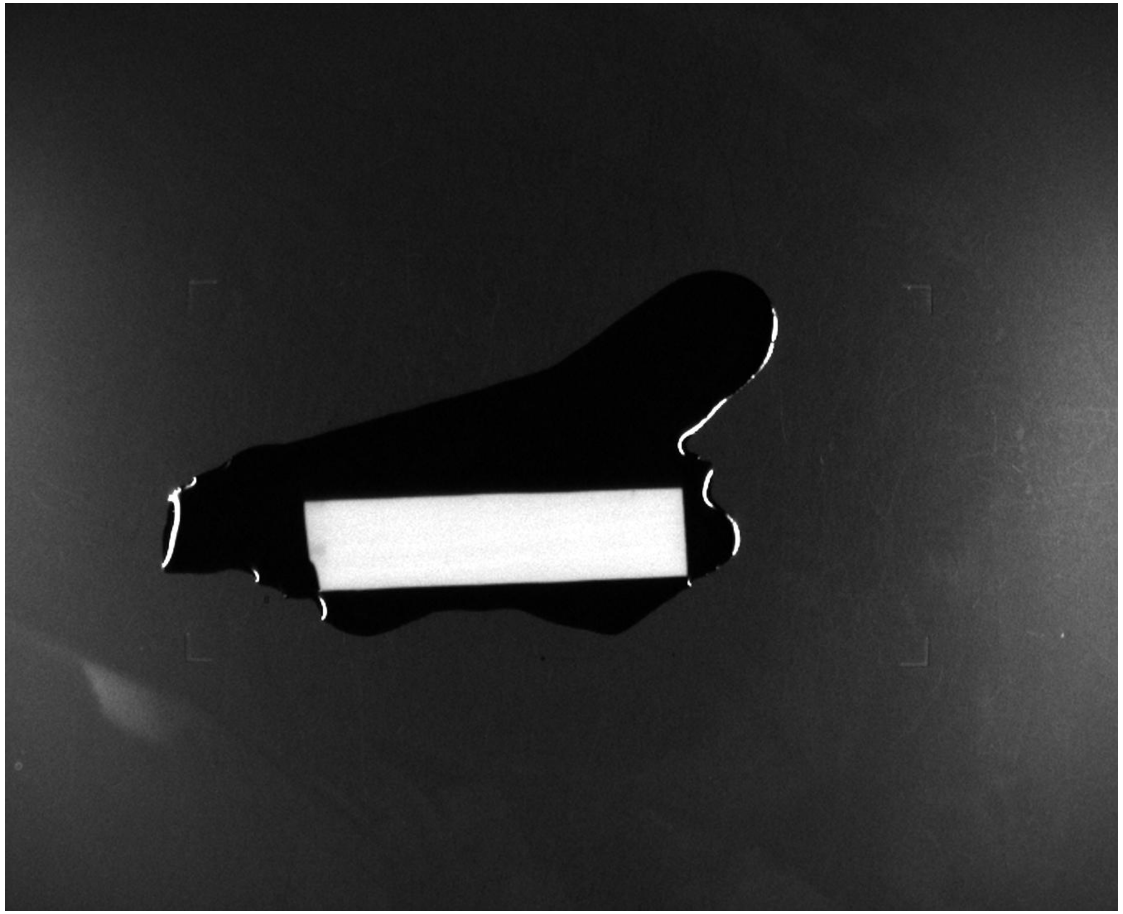

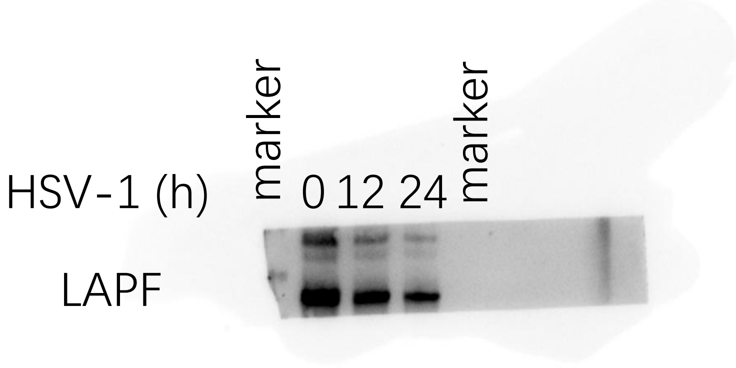

Fig. 1O

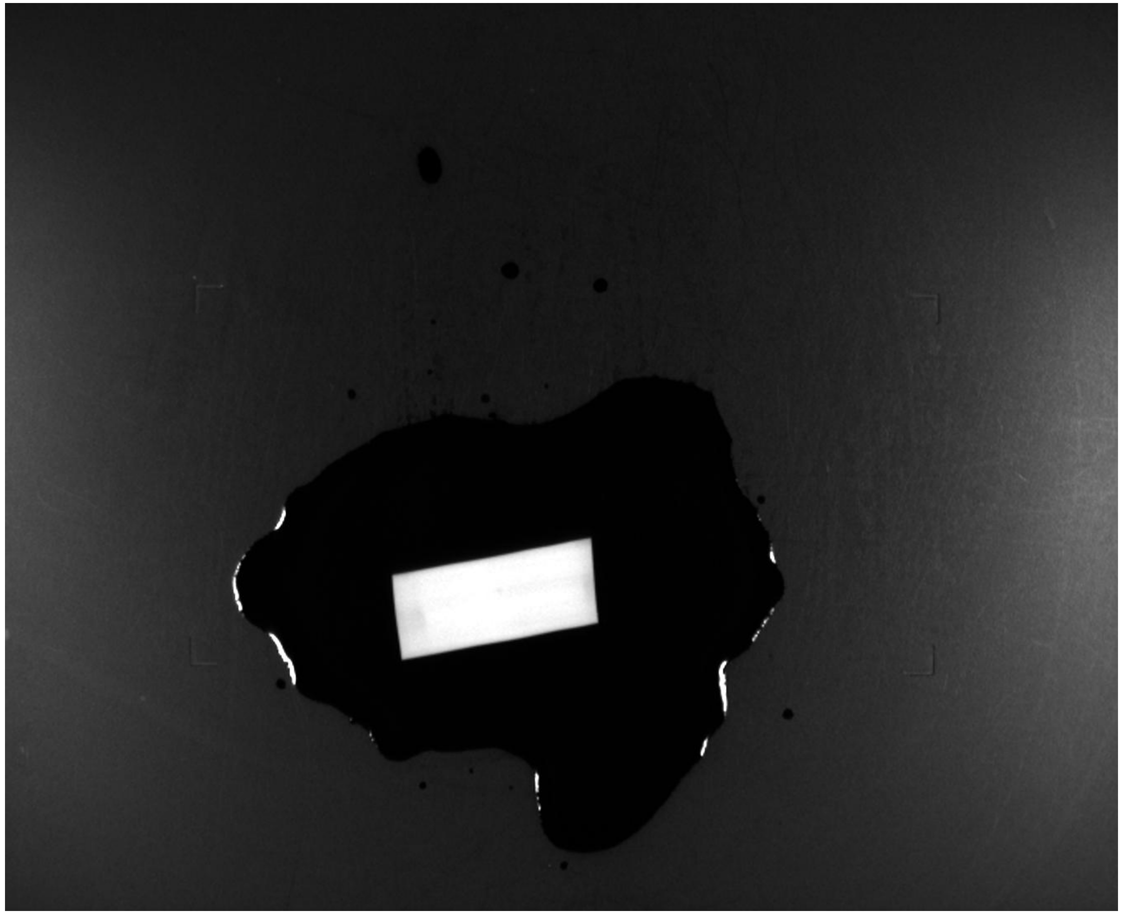

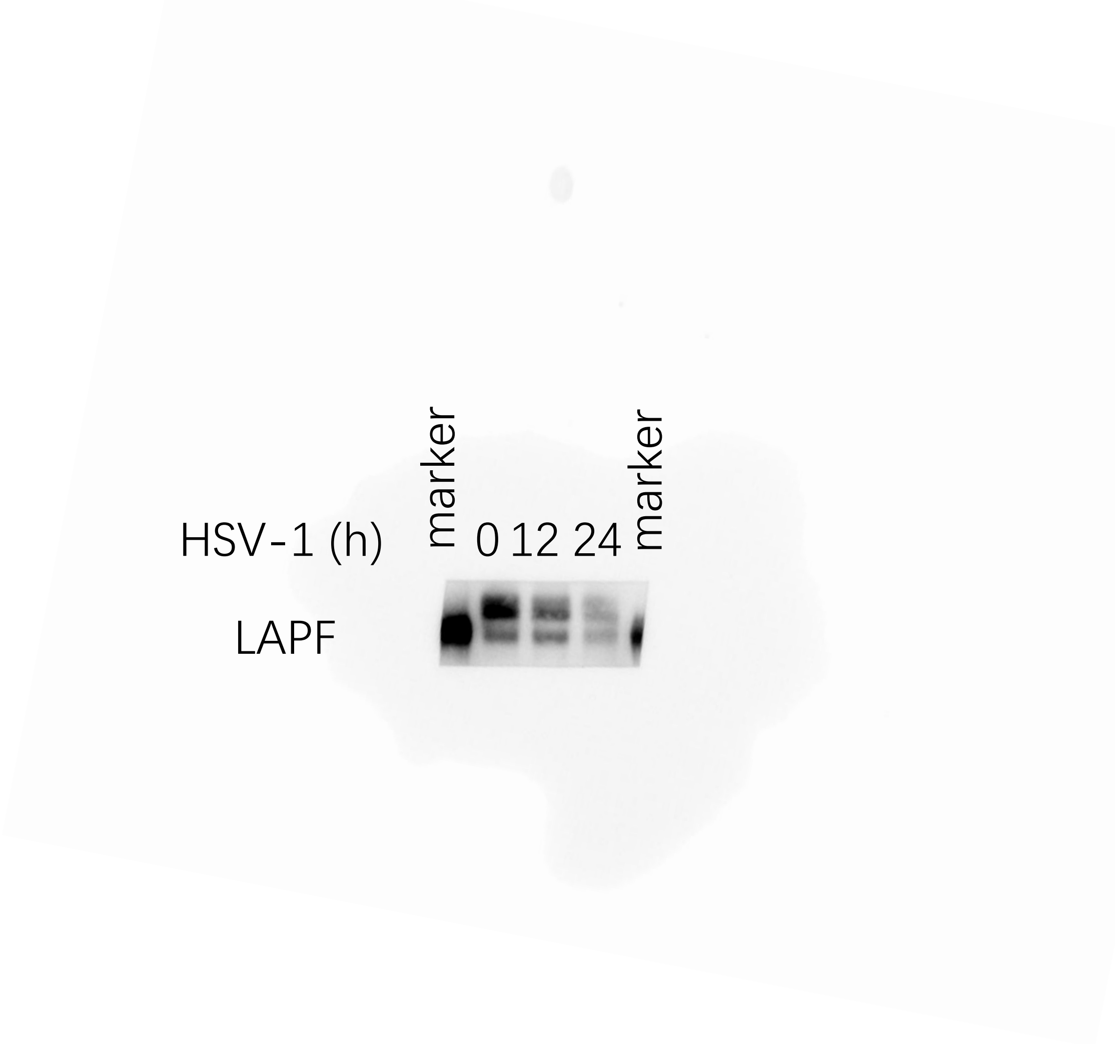

Fig. 1O

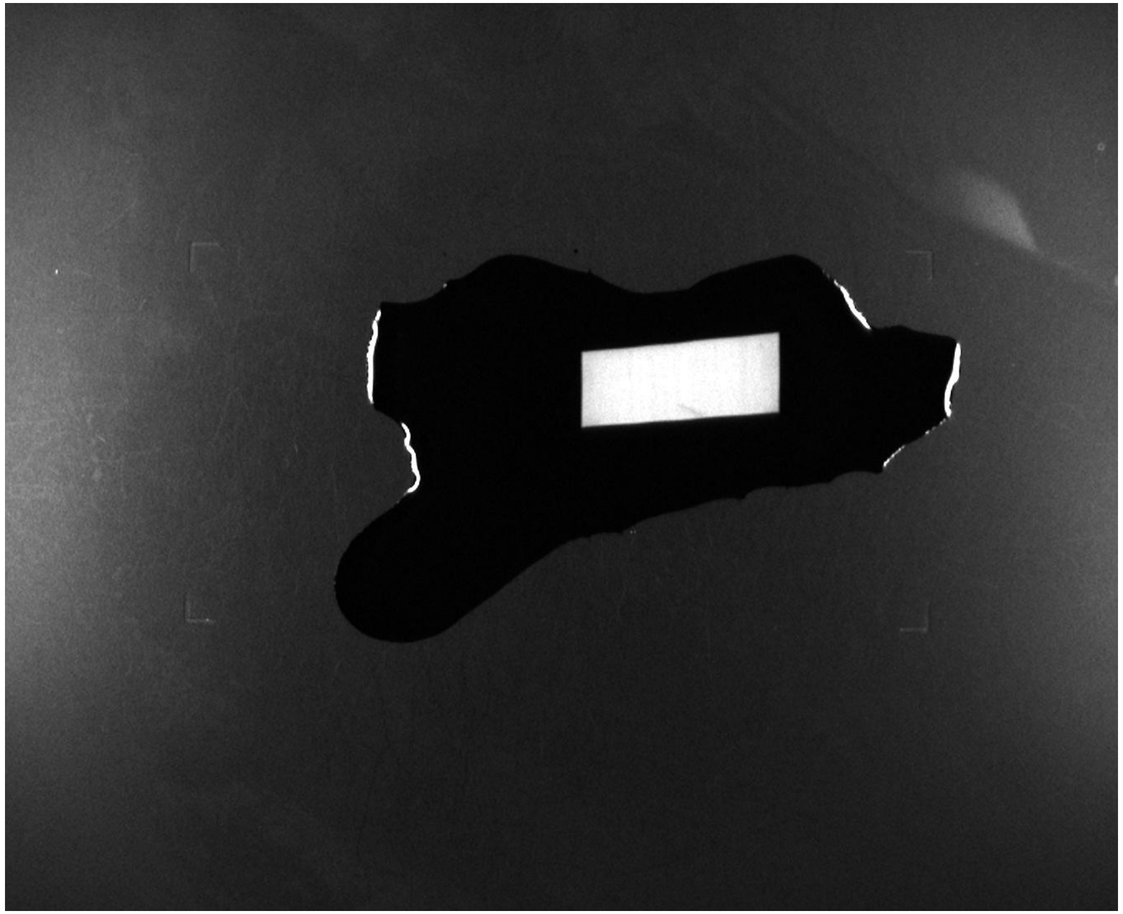

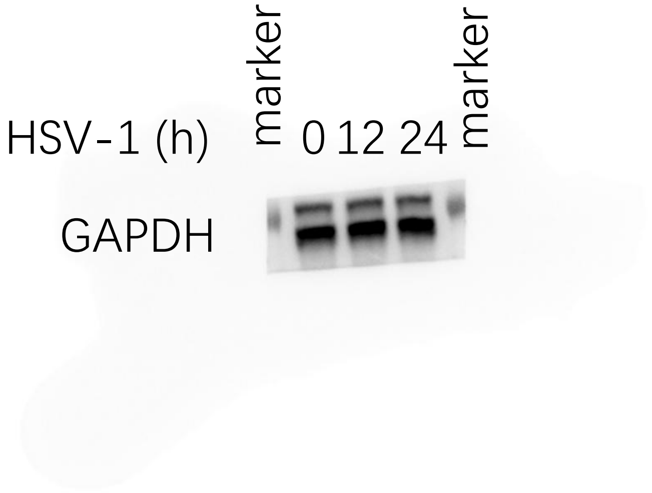

Fig. 1O

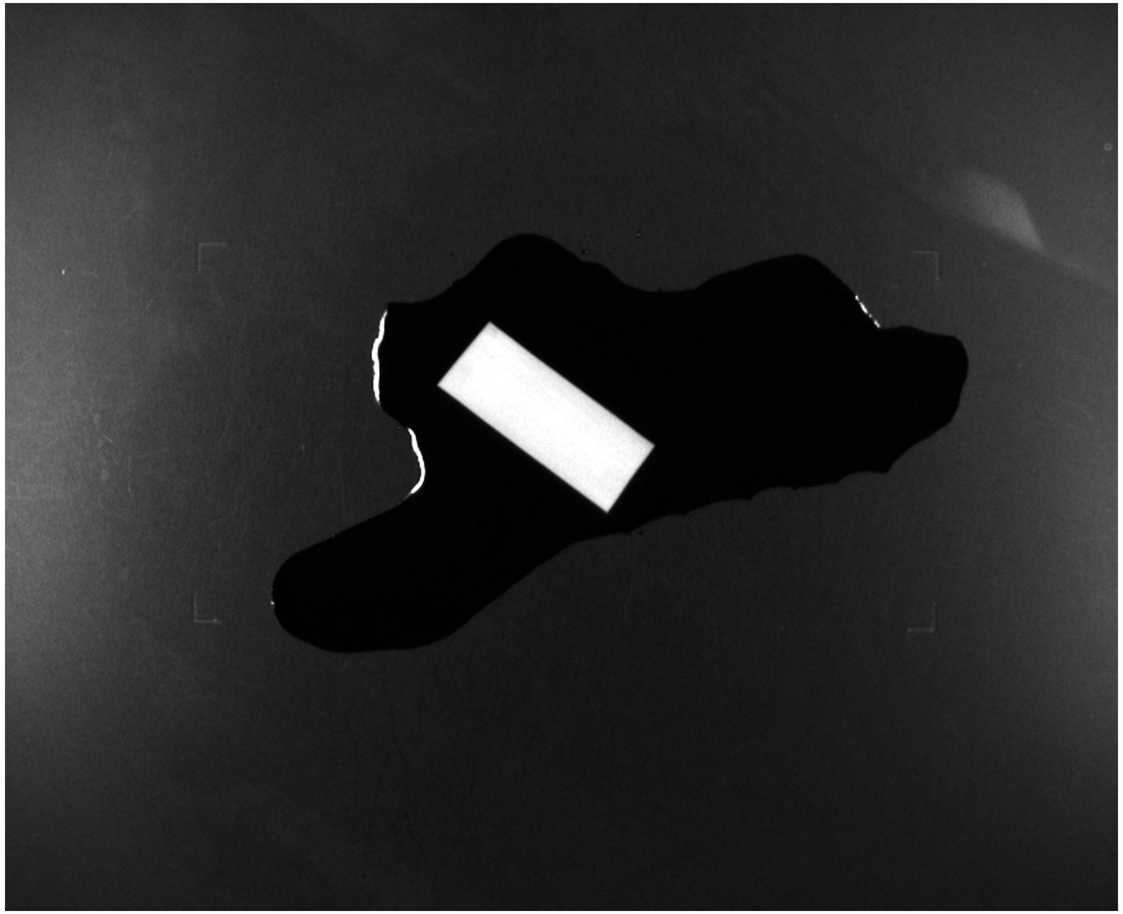

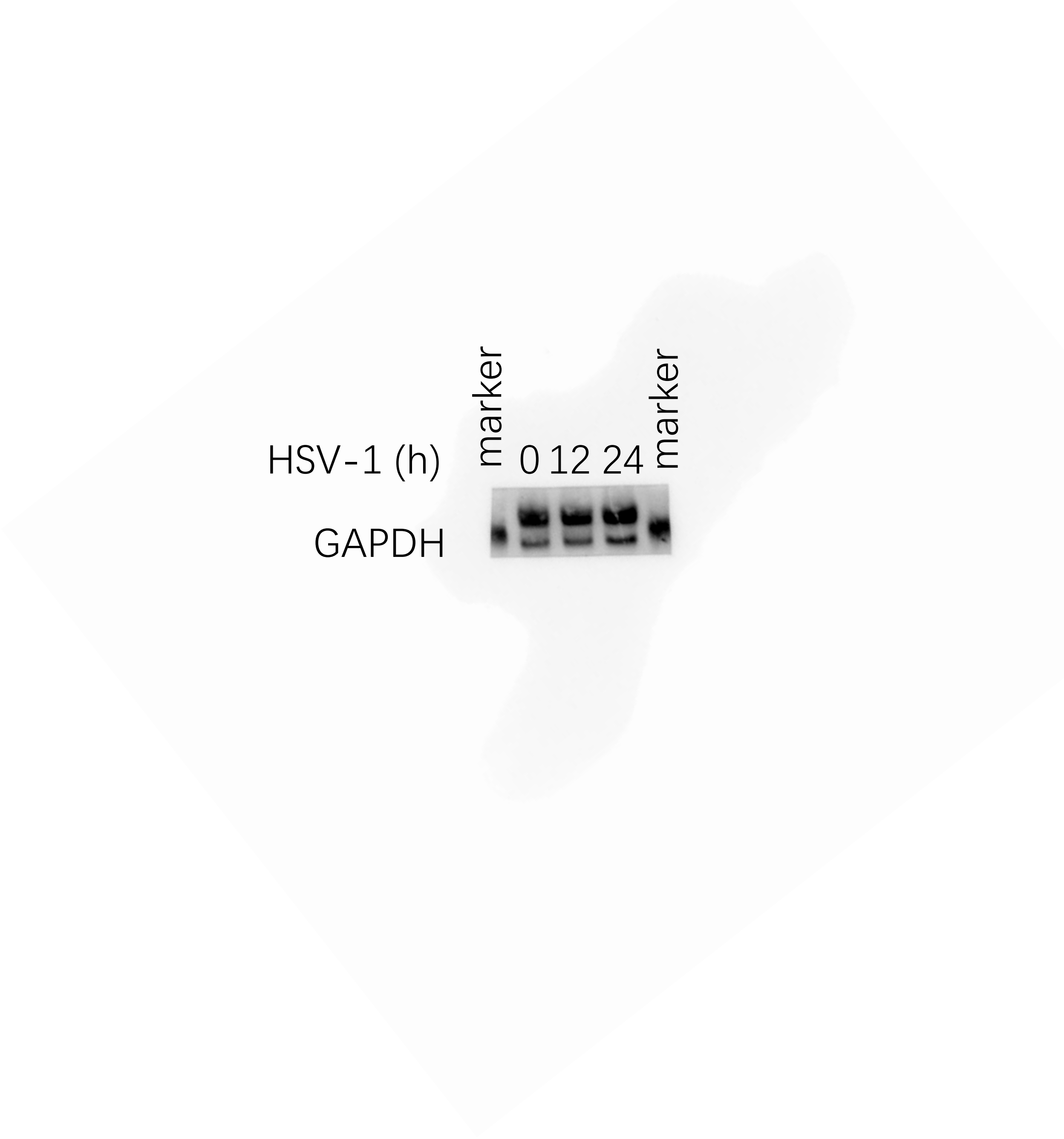

Fig. 1O

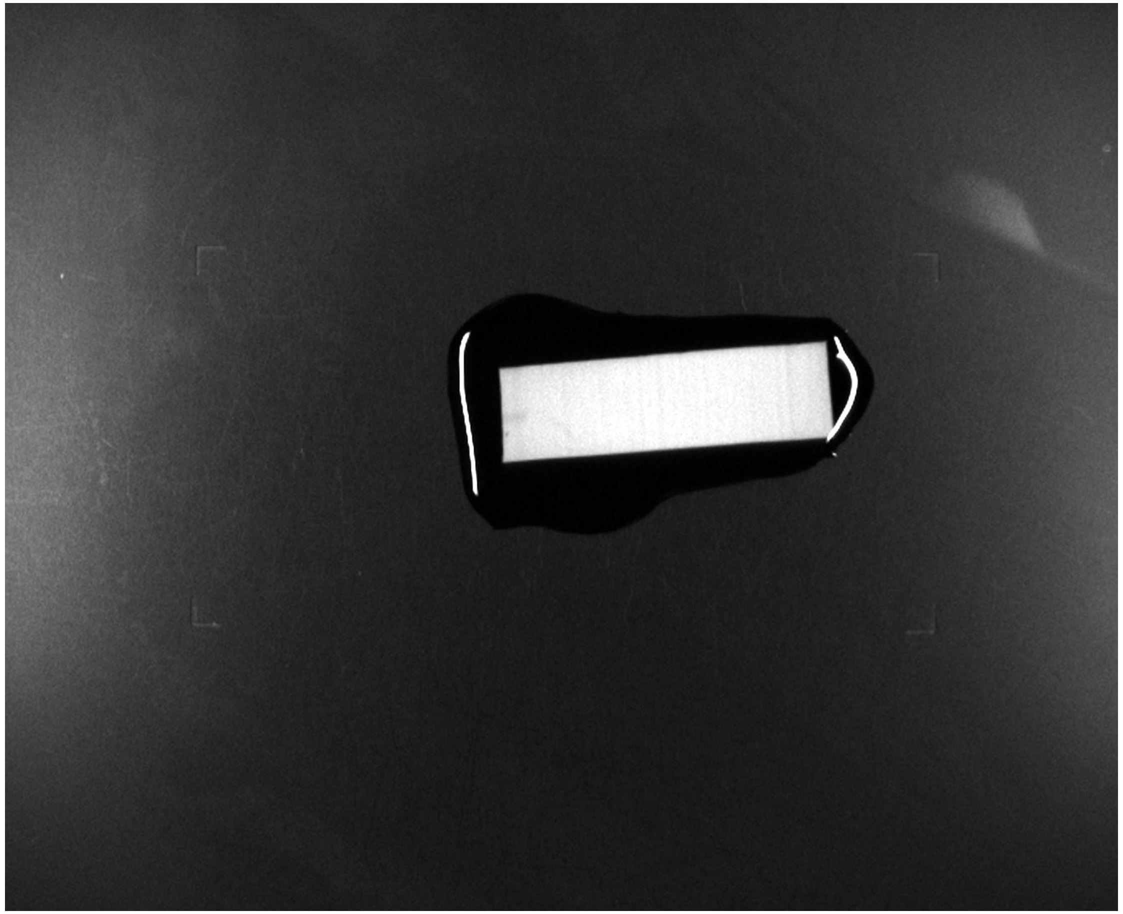

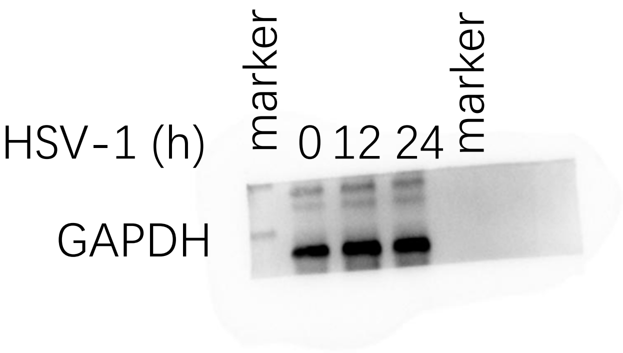

Fig. 5C

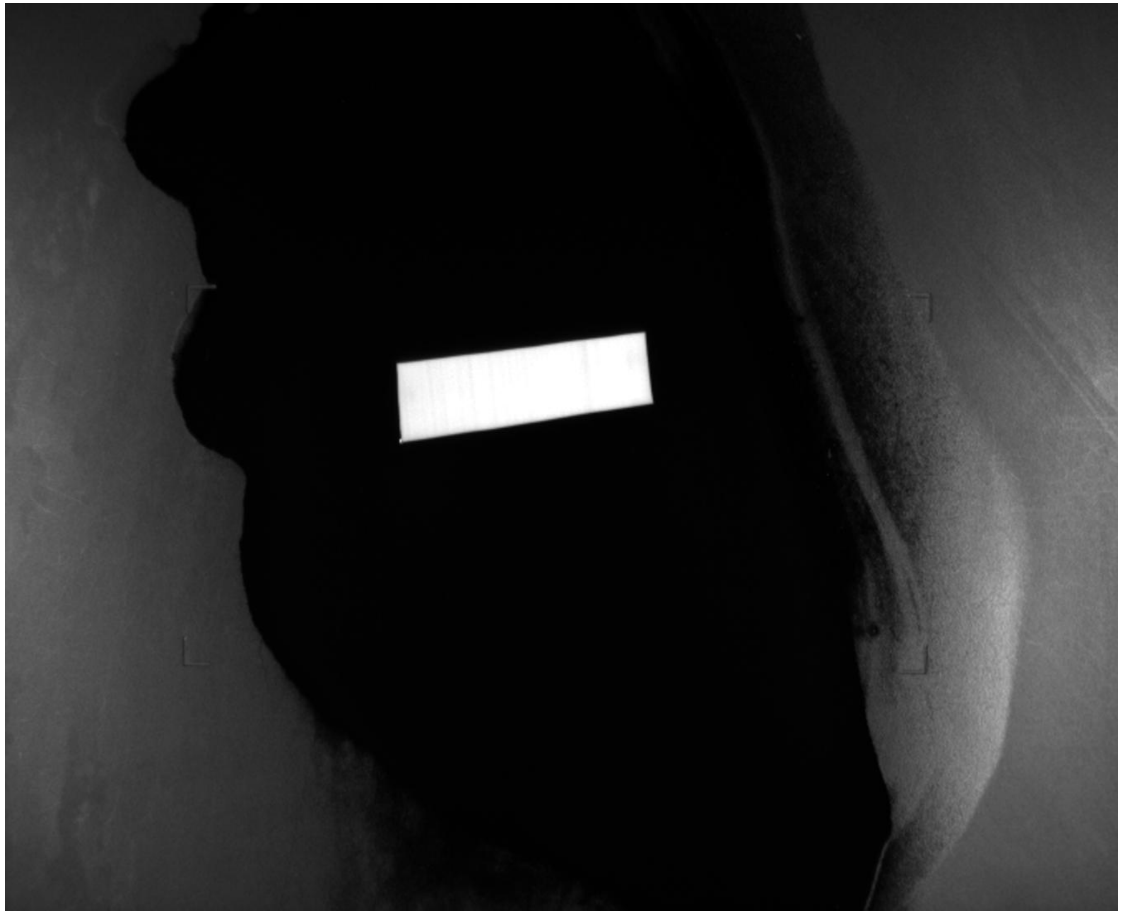

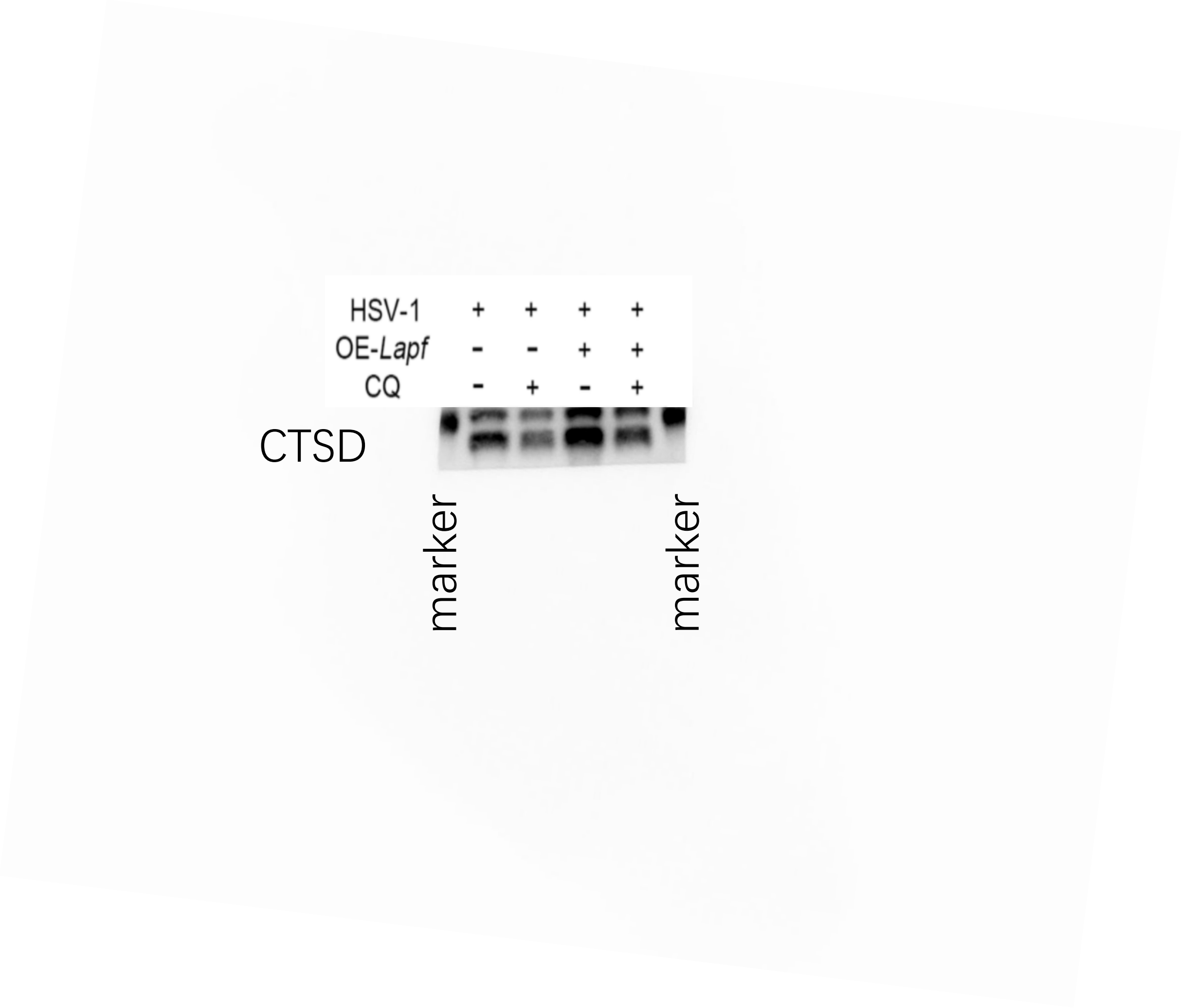

Fig. 5C

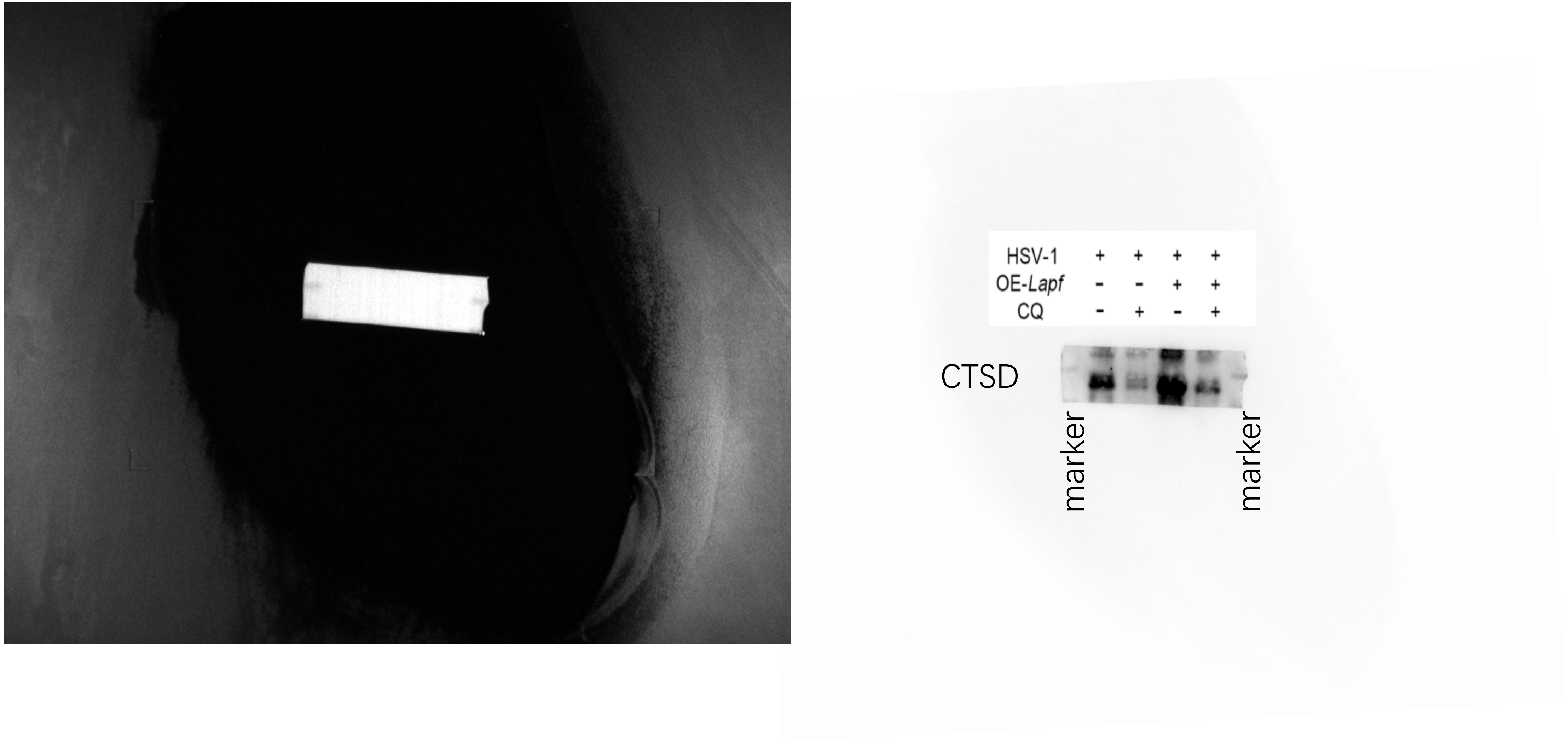

Fig. 5C

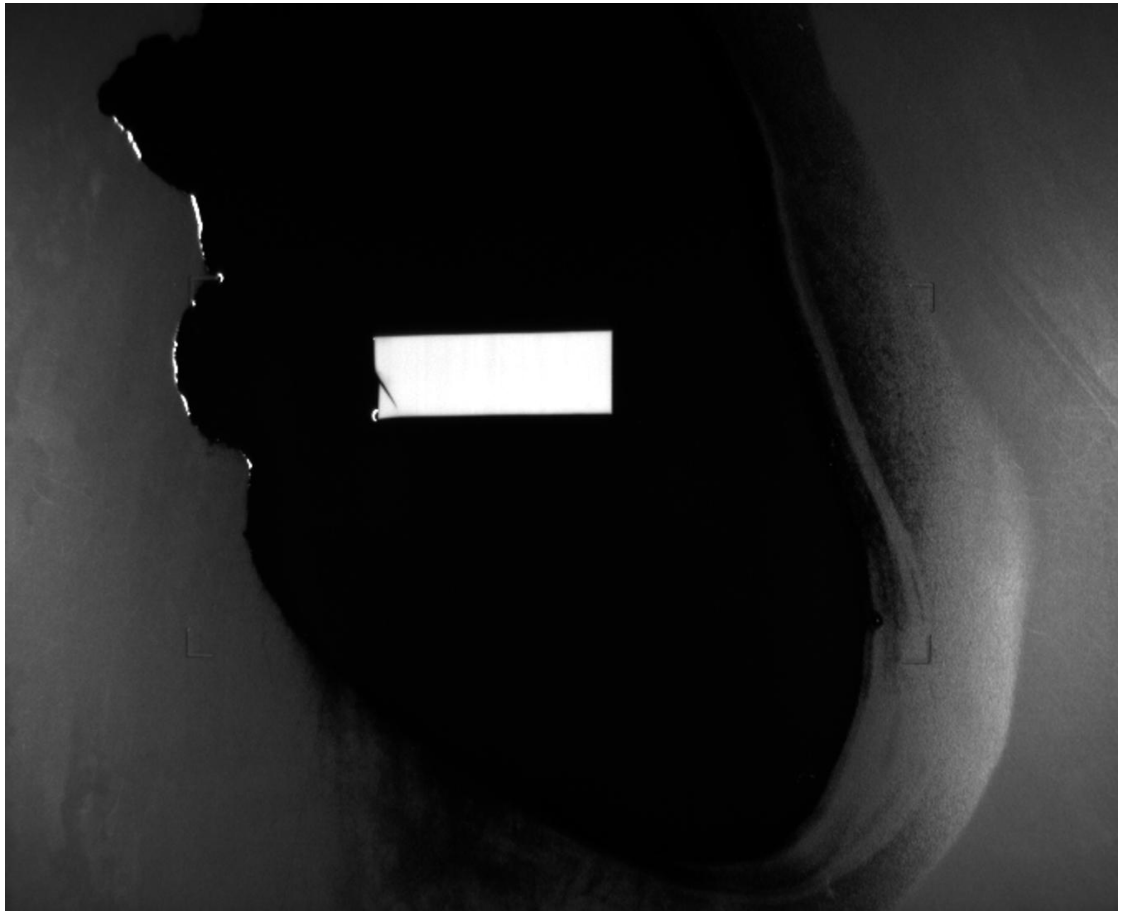

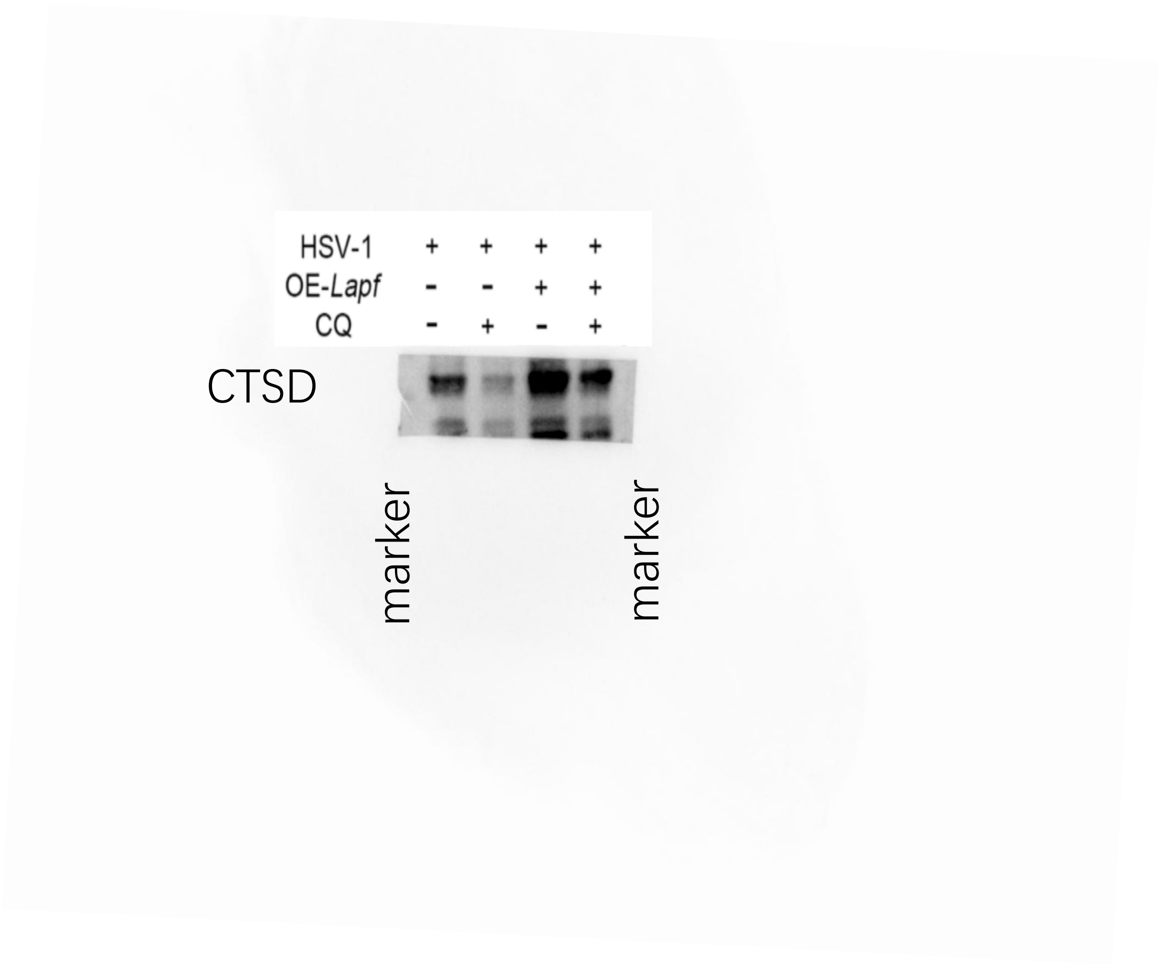

Fig. 5C

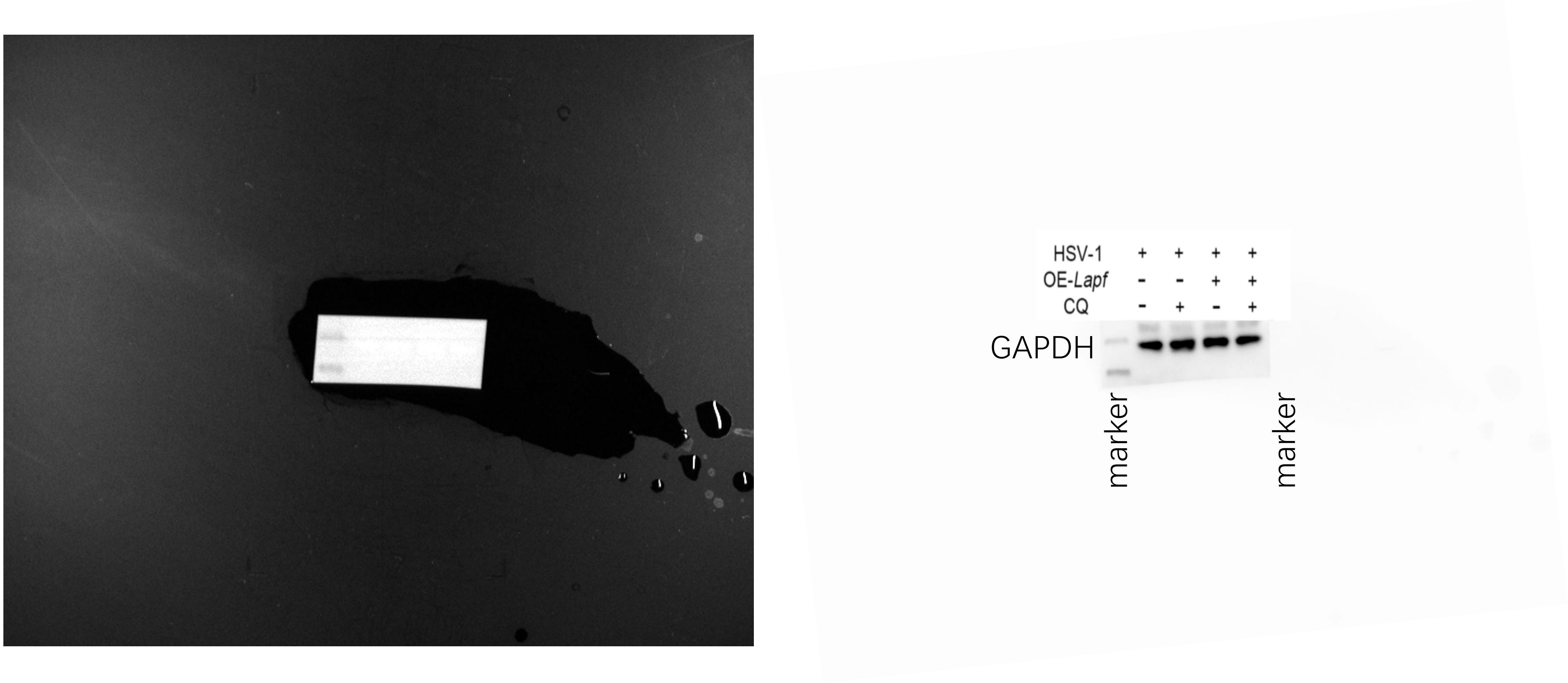

Fig. 5C

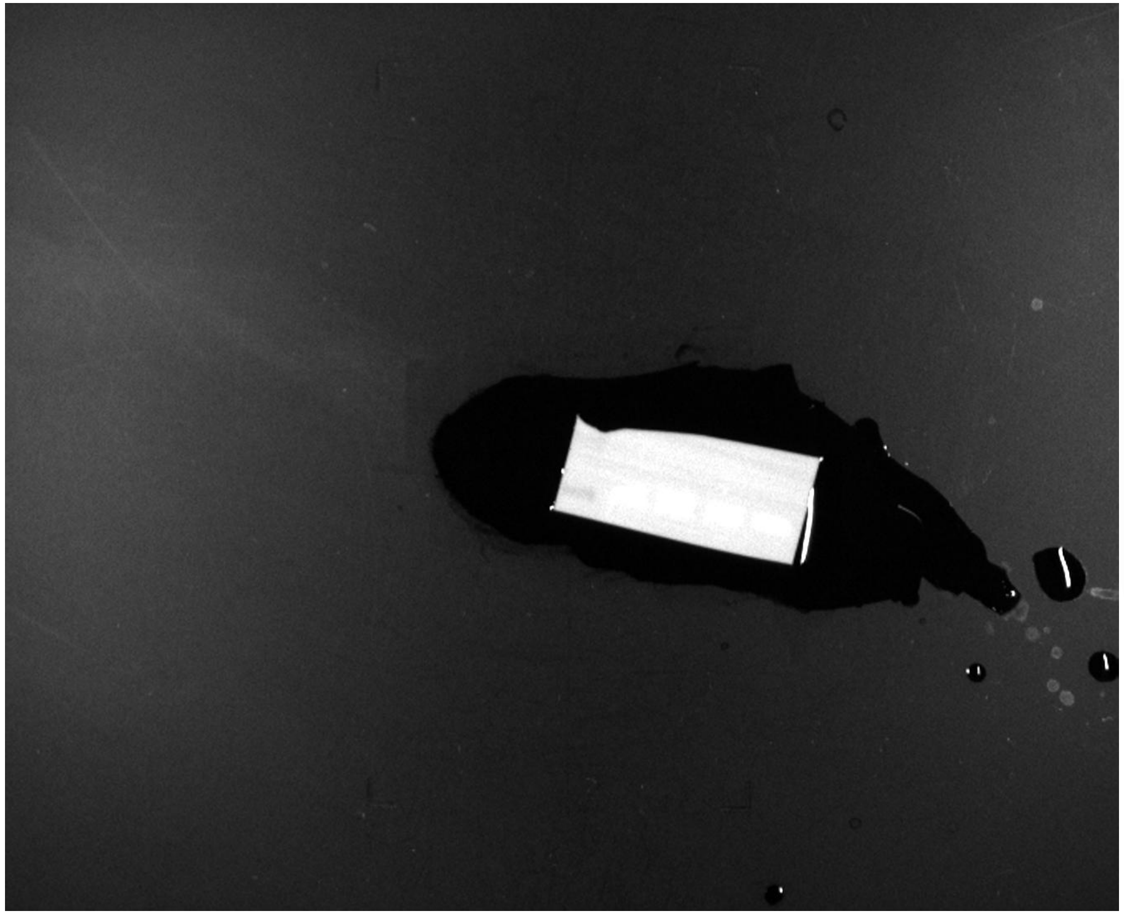

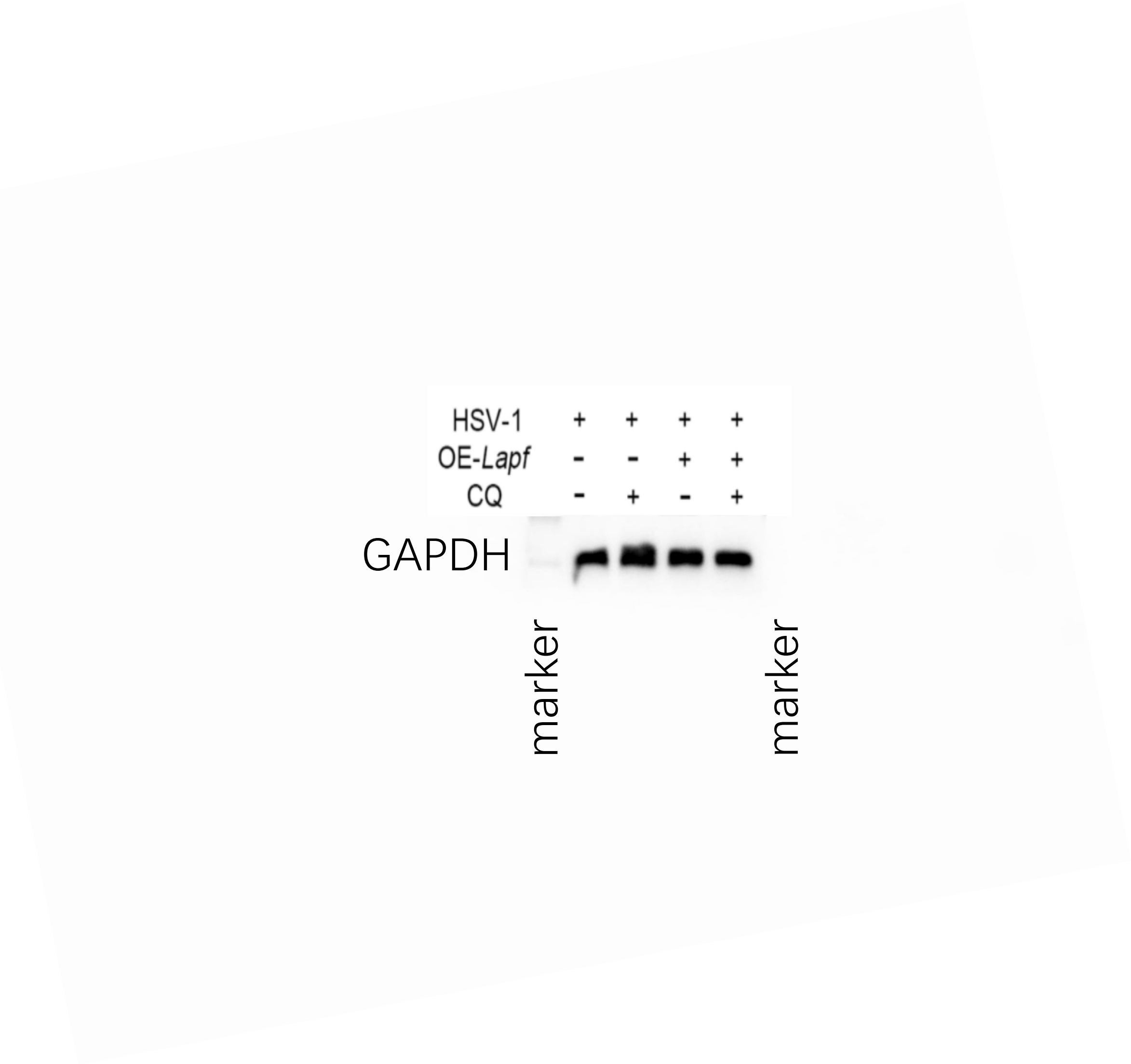

Fig. 5C

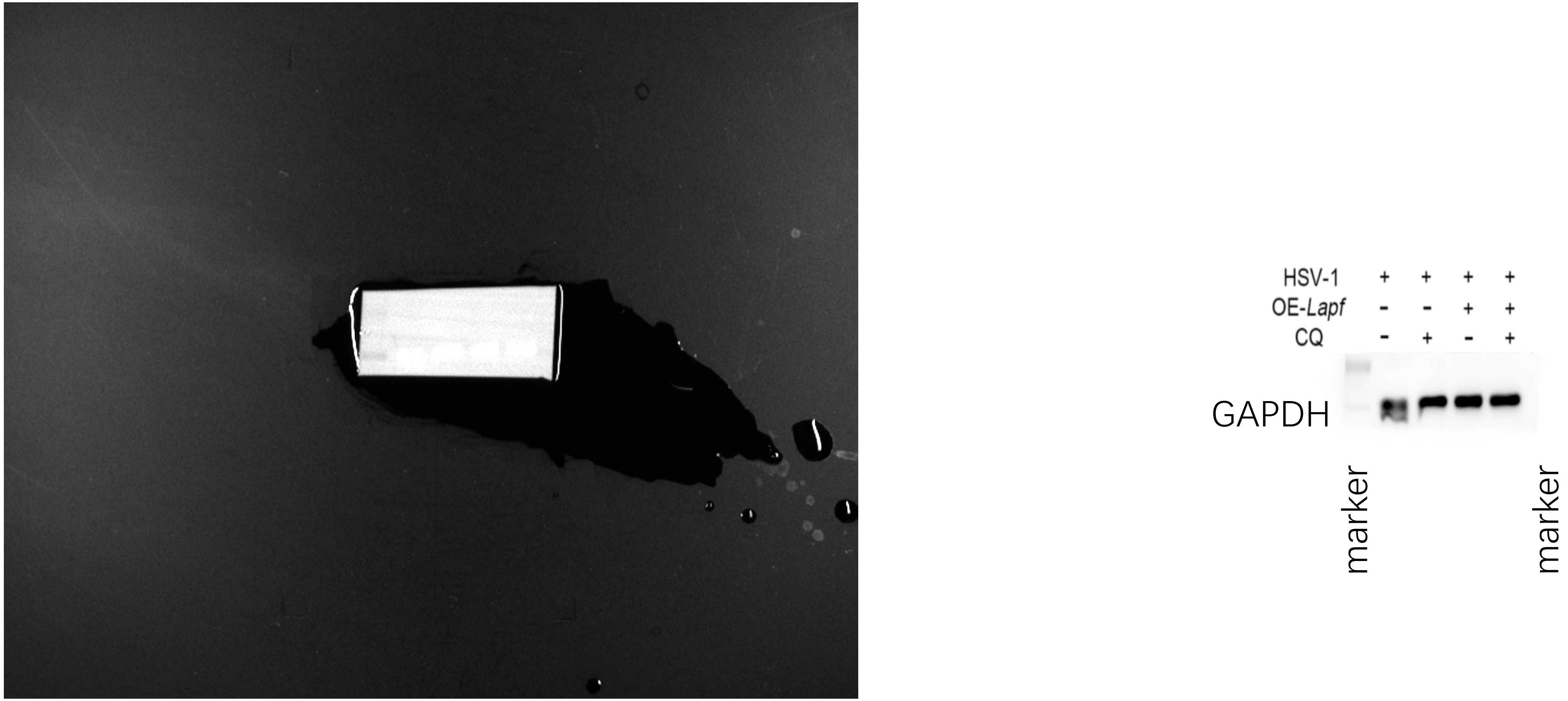

Ssp Fig. 1B

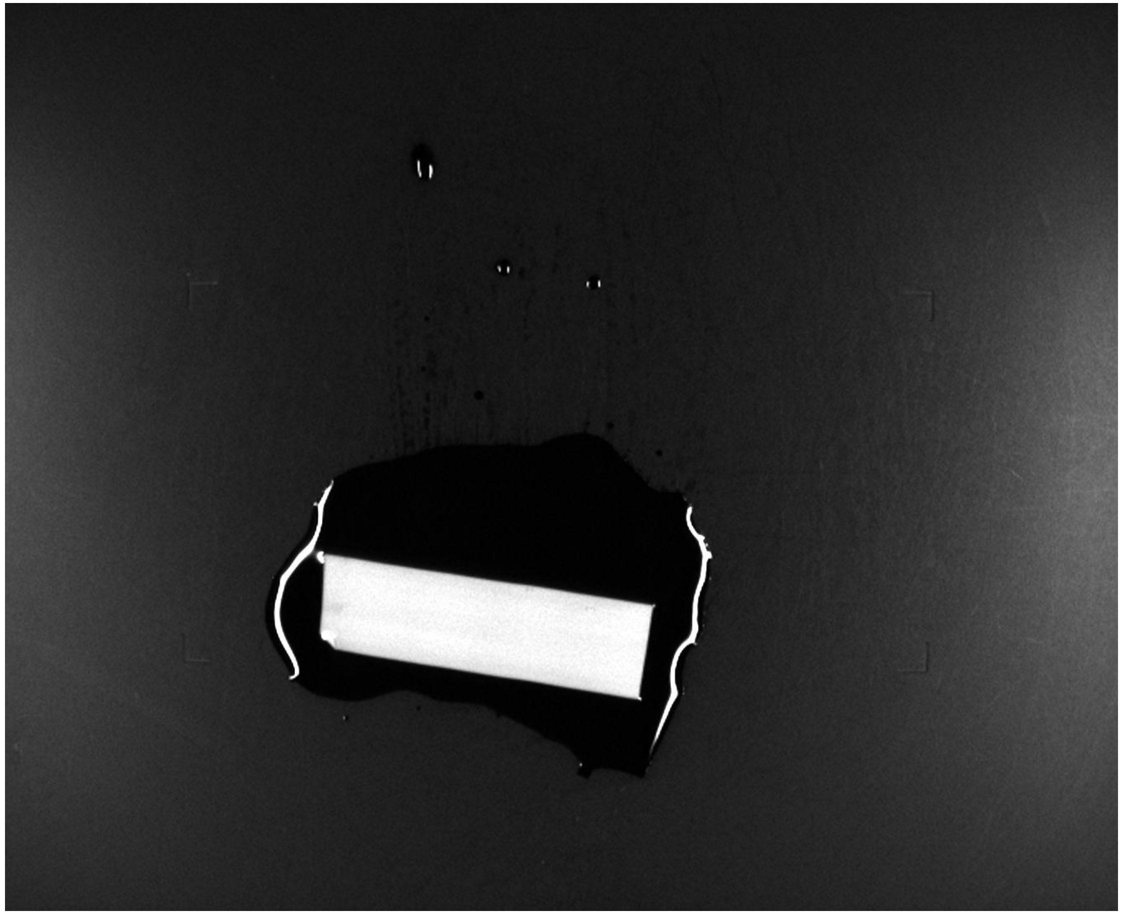

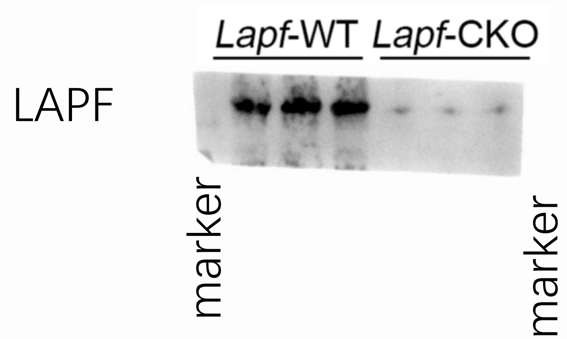

Ssp Fig. 1B

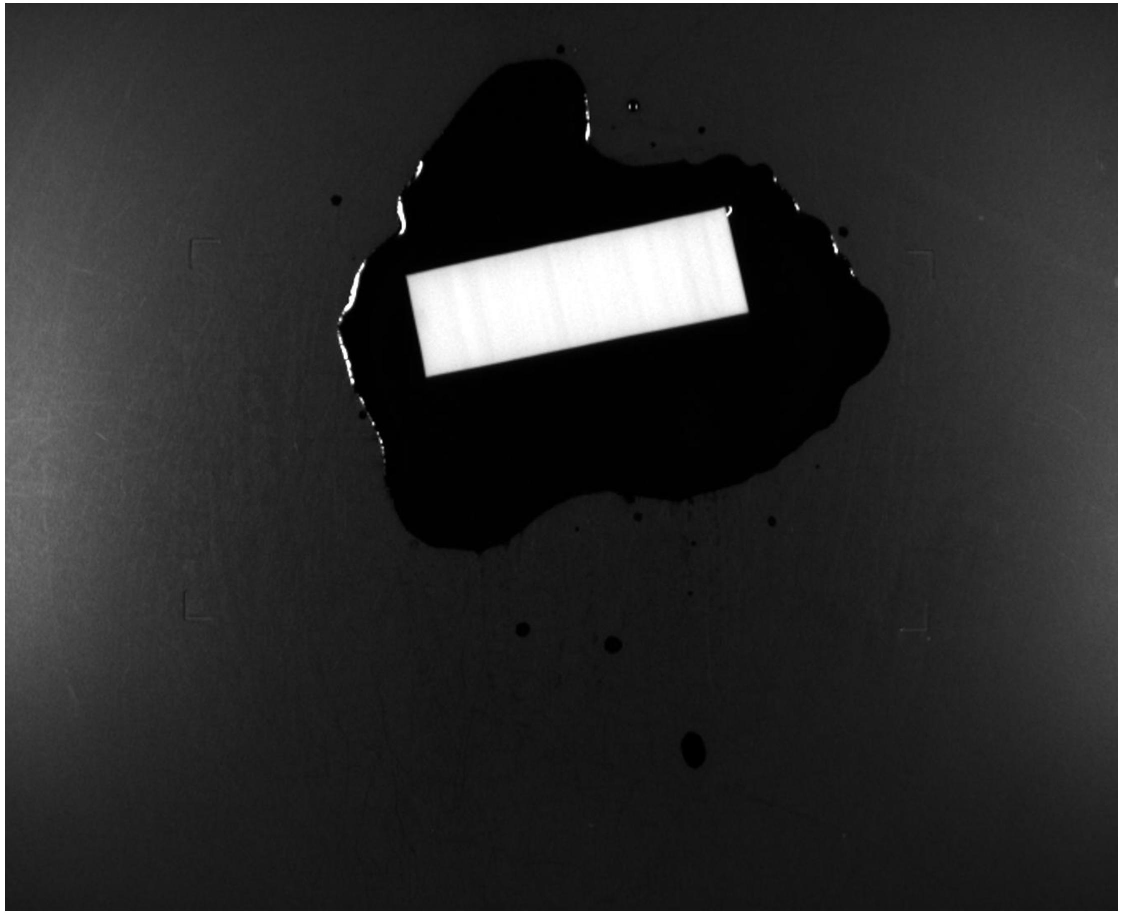

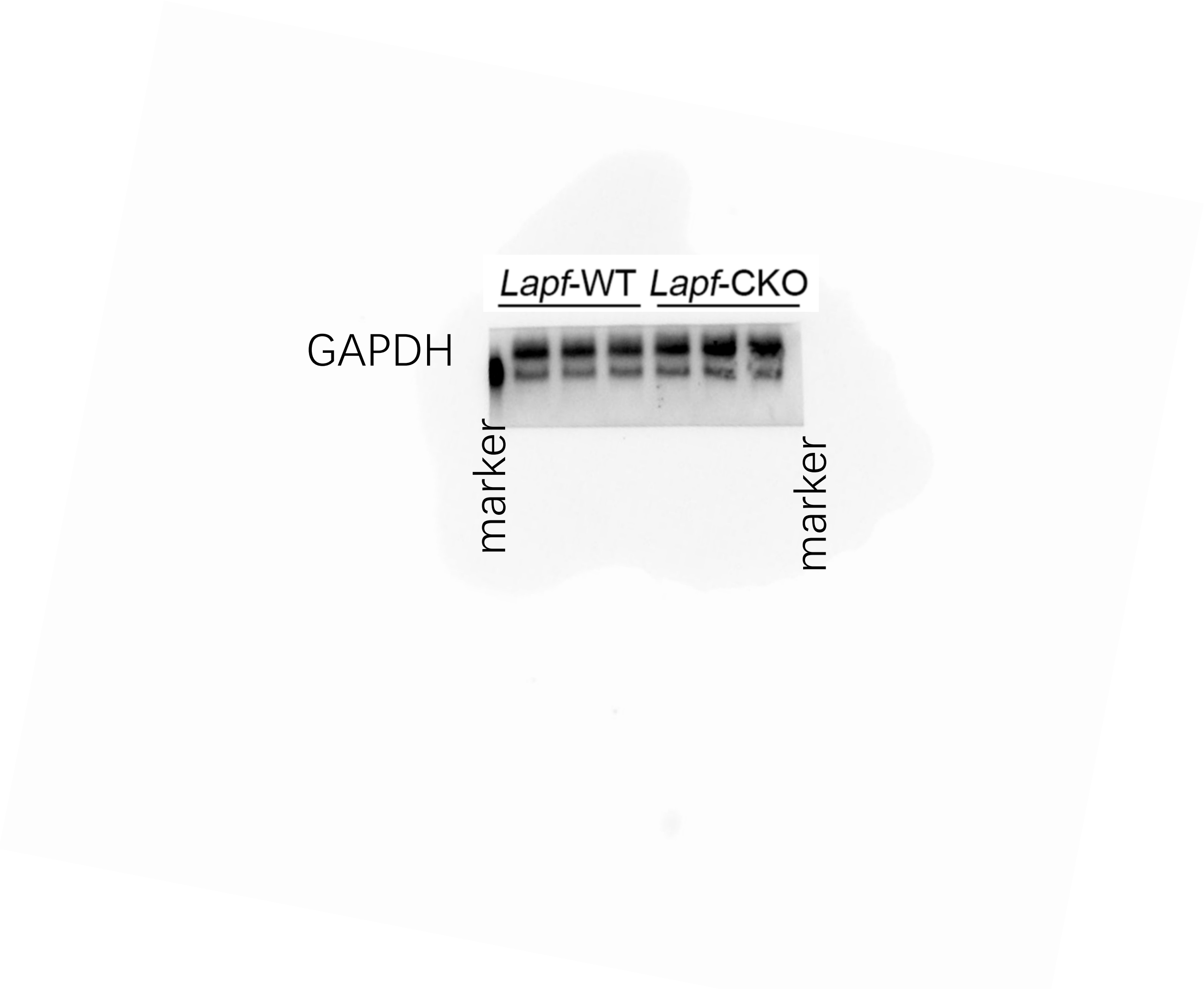

Ssp Fig. 1D

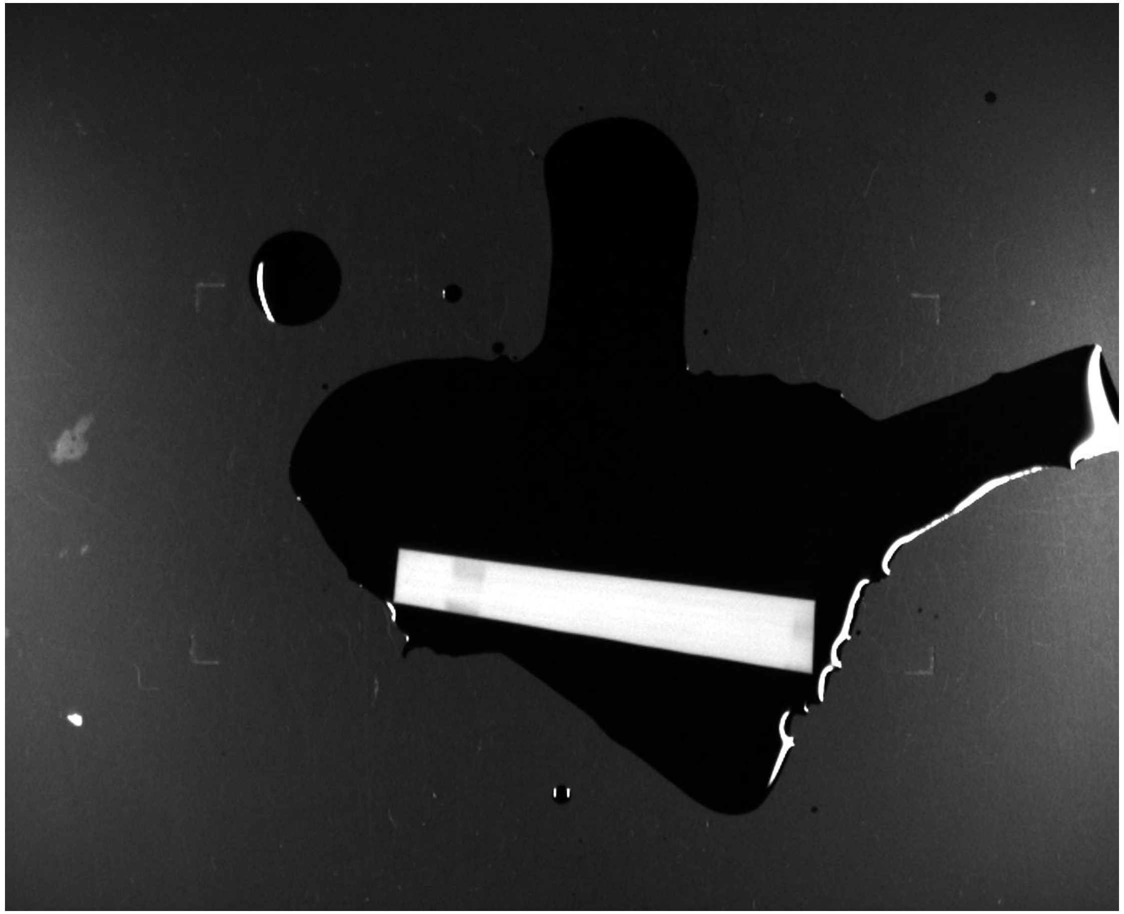

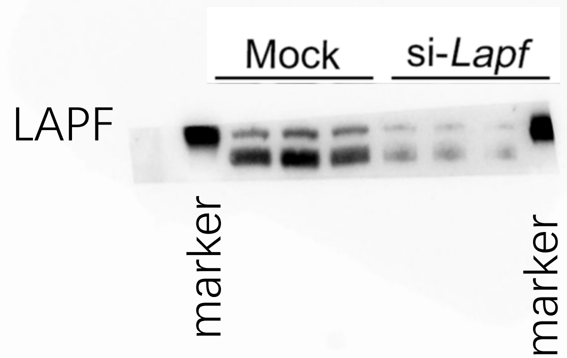

Ssp Fig. 1D

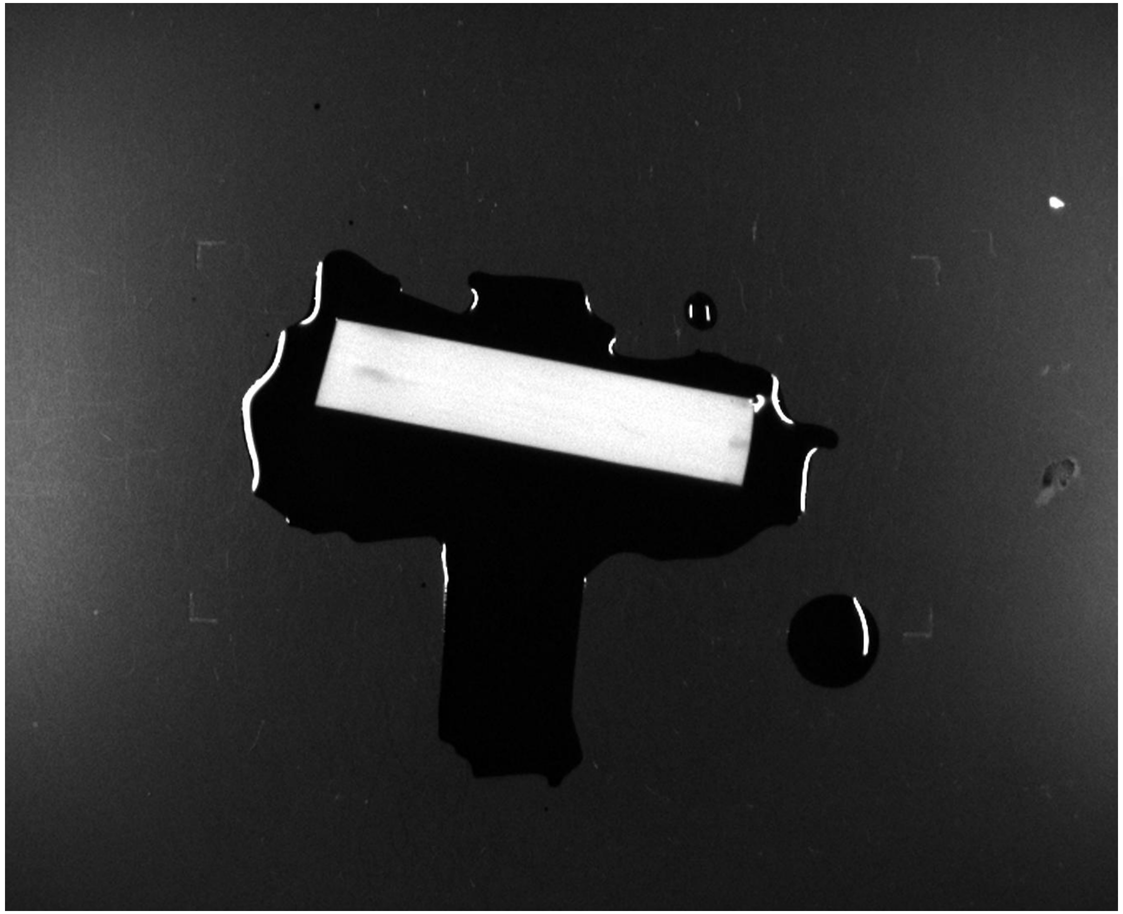

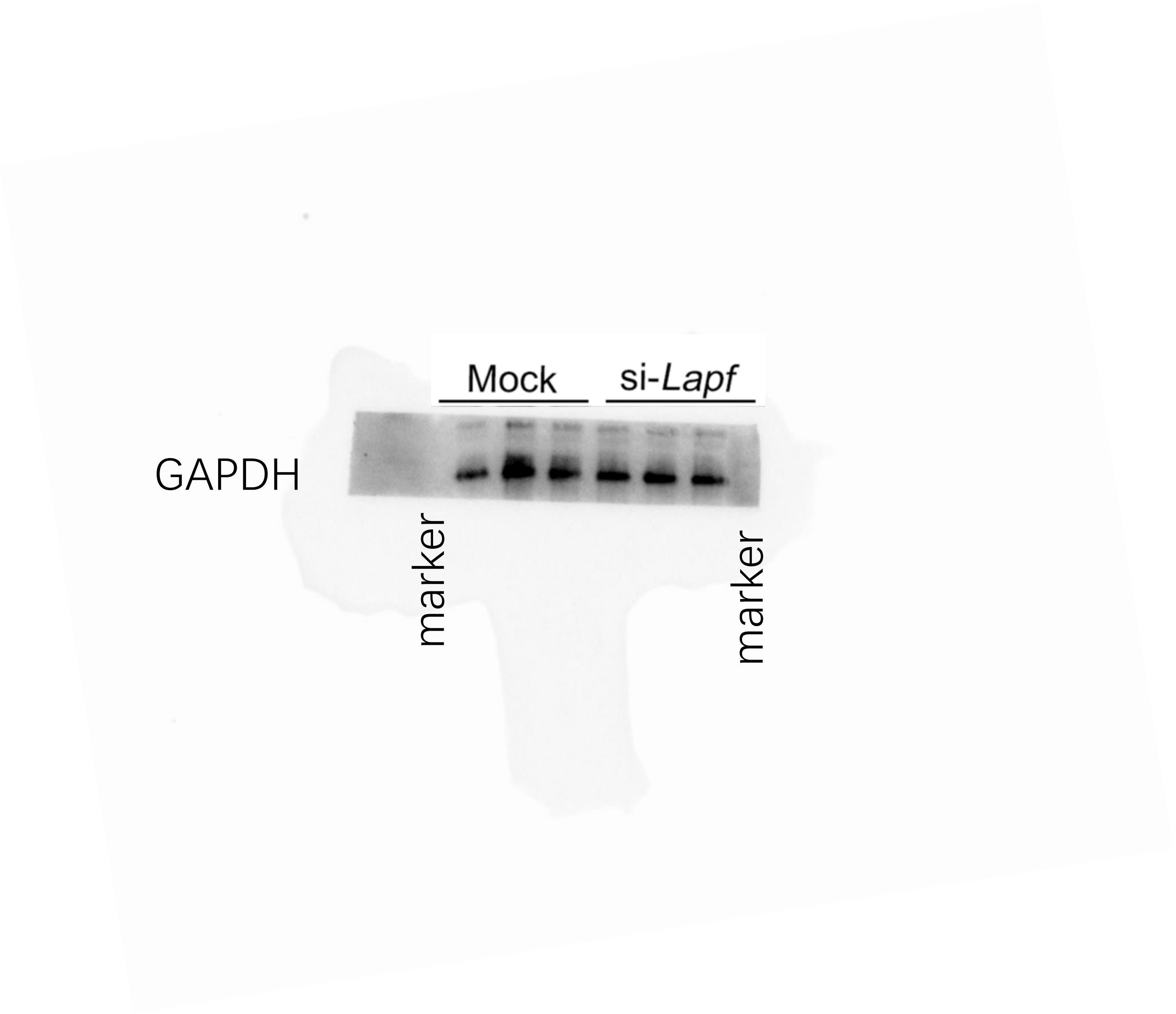

Ssp Fig. 1F

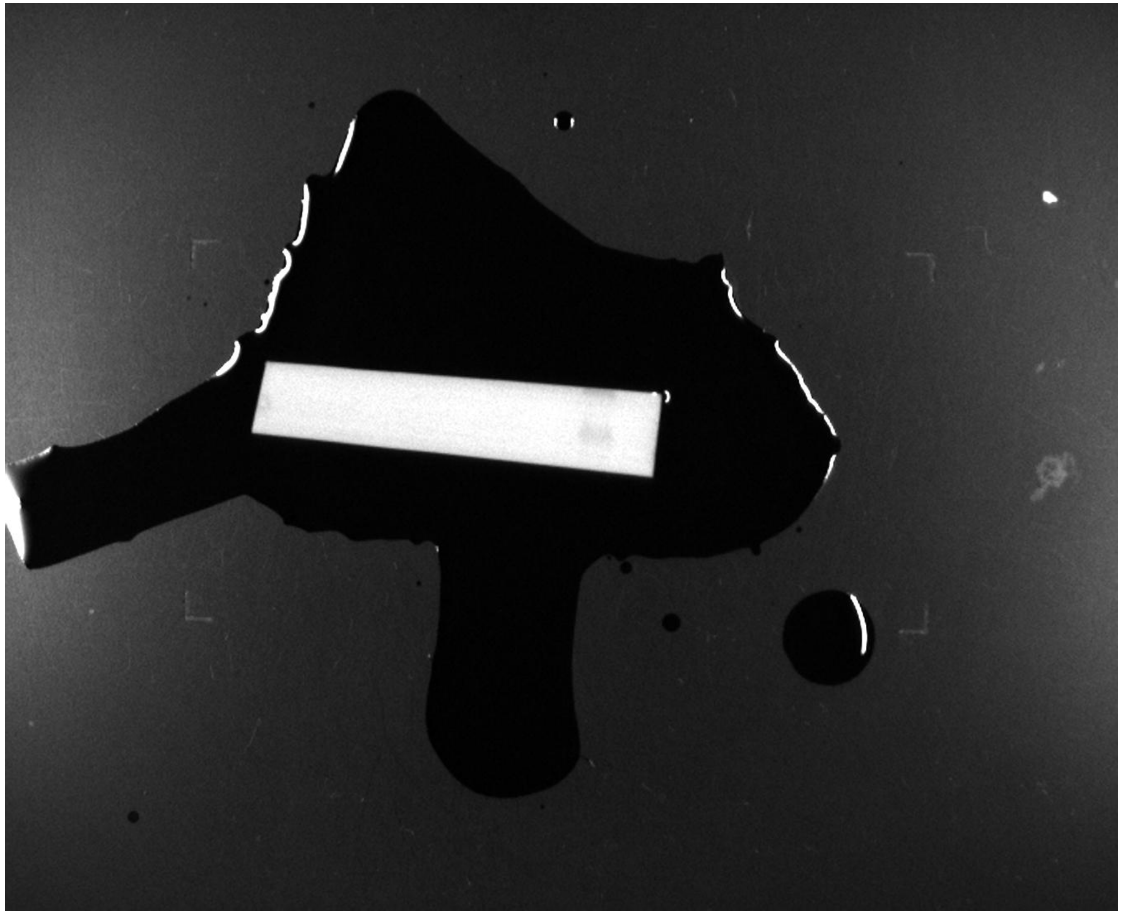

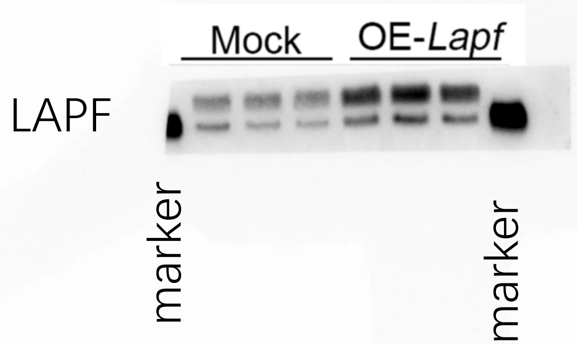

Ssp Fig. 1F

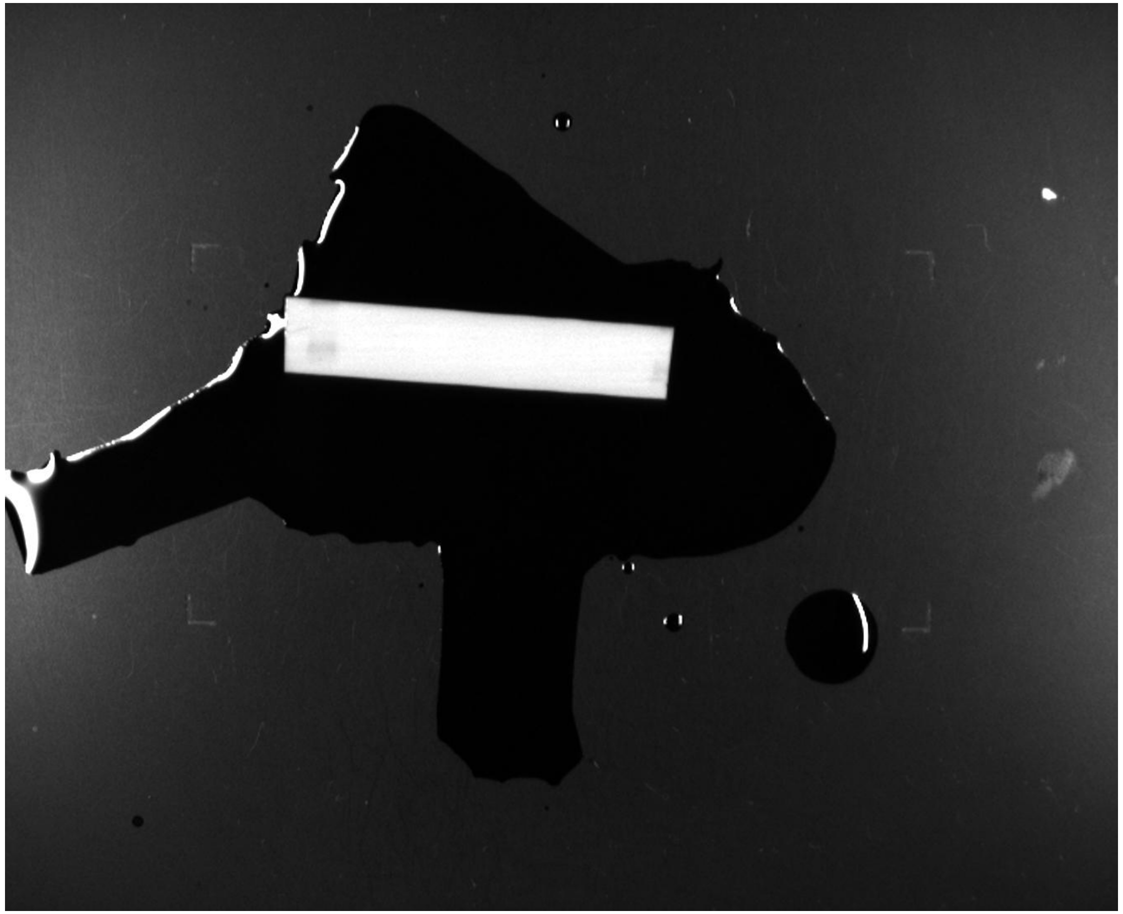

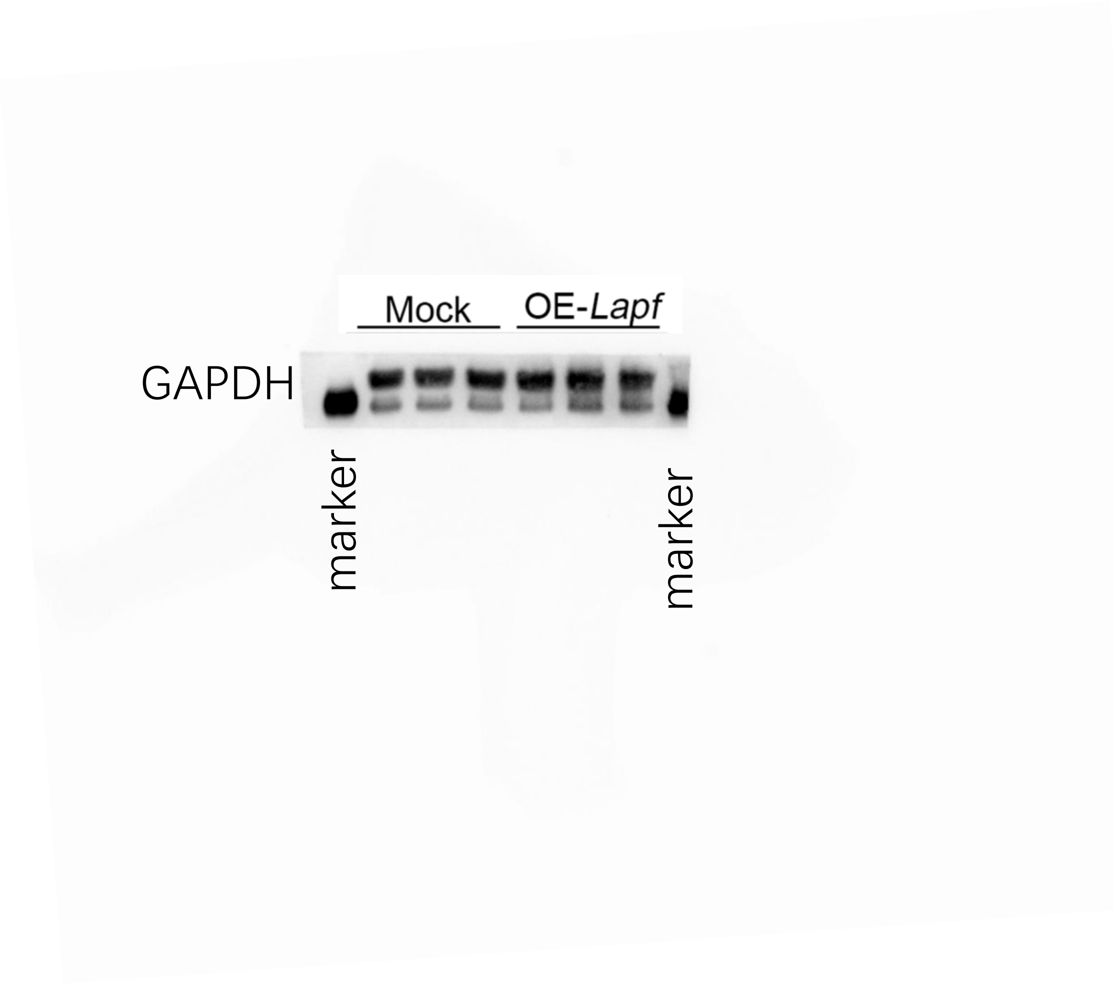

Supplement: Supplementary file 1 — Supplementary Material 1. [file 12974_2026_3856_MOESM1_ESM.pdf]
