# Supplementary material for: LAPF enhances lysosomal acidification to promote TLR9 and cGAS-STING-mediated antiviral immunity and attenuate HSV-1-induced neuroinflammatory pain
Source: J Neuroinflammation. 2026 May 8;23:207. doi: 10.1186/s12974-026-03856-6 (PMC13285062; doi:10.1186/s12974-026-03856-6)
Supplement: Supplementary file 2 — Supplementary Material 2. [file 12974_2026_3856_MOESM2_ESM.docx]

**Supplementary Table 1 Primer sequences**

| **Gene** | **Forward** | **Reverse** |
| --- | --- | --- |
| Mouse LAPF | 5’-TGGAGAGCAGTGGGTTGAAG-3’ | 5’-CGGGTCAGGAGCCTGAATAA-3’ |
| Mouse IFN-α | 5’-TACTCAGCAGACCTTGAACCT-3’ | 5’-CAGTCTTGGCAGCAAGTTGAC-3’ |
| Mouse IFN-β | 5’-ATGAGTGGTGGTTGCAGGC-3’ | 5’-TGACCTTTCAAATGCAGTAGATTCA-3’ |
| Mouse CXCL10 | 5’-TCTGAGTGGGACTCAAGGGAT-3’ | 5’-GAGGCTCTCTGCTGTCCATC-3’ |
| Mouse ISG15 | 5’-TCTGACTGTGAGAGCAAGCAG-3’ | 5’-ACCTTTAGGTCCCAGGCCATT-3’ |
| TK gene of HSV-1 | 5’-ATACCGACGATATGCGACCT-3’ | 5’-TTATTGCCGTCATAGCGCGG-3’ |
| Mouse TNF-α | 5’-GTAGCCCACGTCGTAGCAAA-3’ | 5’-ACAAGGTACAACCCATCGGC-3’ |
| Mouse IL-1β | 5’-AGAGCCCATCCTCTGTGACT-3’ | 5’-GCTCATATGGGTCCGACAGC-3’ |
| Mouse BDNF | 5’-CCCGGTGTCGCCCTTAAAAA-3’ | 5’-CTCACCTGGTGGAACTTCTTTG-3’ |
| Mouse NG | 5’-AGCATCGTACAAACCCACCC-3’ | 5’-AAAACGTTCAGCTCTGGCCTA-3’ |
| Mouse MAG | 5’-GCGTTCCTCAGCTCCTCATT-3’ | 5’-CCCCCTCGAGAAGCTGAAAT-3’ |
| Mouse β-actin | 5’-AGAGGGAAATCGTGCGTGAC-3’ | 5’-CAATAGTGATGACCTGGCCGT-3’ |
