# Supplementary material for: LAPF enhances lysosomal acidification to promote TLR9 and cGAS-STING-mediated antiviral immunity and attenuate HSV-1-induced neuroinflammatory pain
Source: J Neuroinflammation. 2026 May 8;23:207. doi: 10.1186/s12974-026-03856-6 (PMC13285062; doi:10.1186/s12974-026-03856-6)
Supplement: Supplementary file 3 — Supplementary Material 3: Supplementary Fig. 1. (A) DNA gel electrophoresis to identify Lapf-WT and Lapf-CKO mice. (B, C) Efficiency of Lapf knockout in primary microglia by western blotting (B) and Q-PCR (C). (D, E) Efficiency of Lapf knockdown by western blotting (D) and Q-PCR (E). (F, G) Efficiency of Lapf overexpression by western blotting (F) and Q-PCR (G). [file 12974_2026_3856_MOESM3_ESM.docx]

**Supplementary Figure 1**


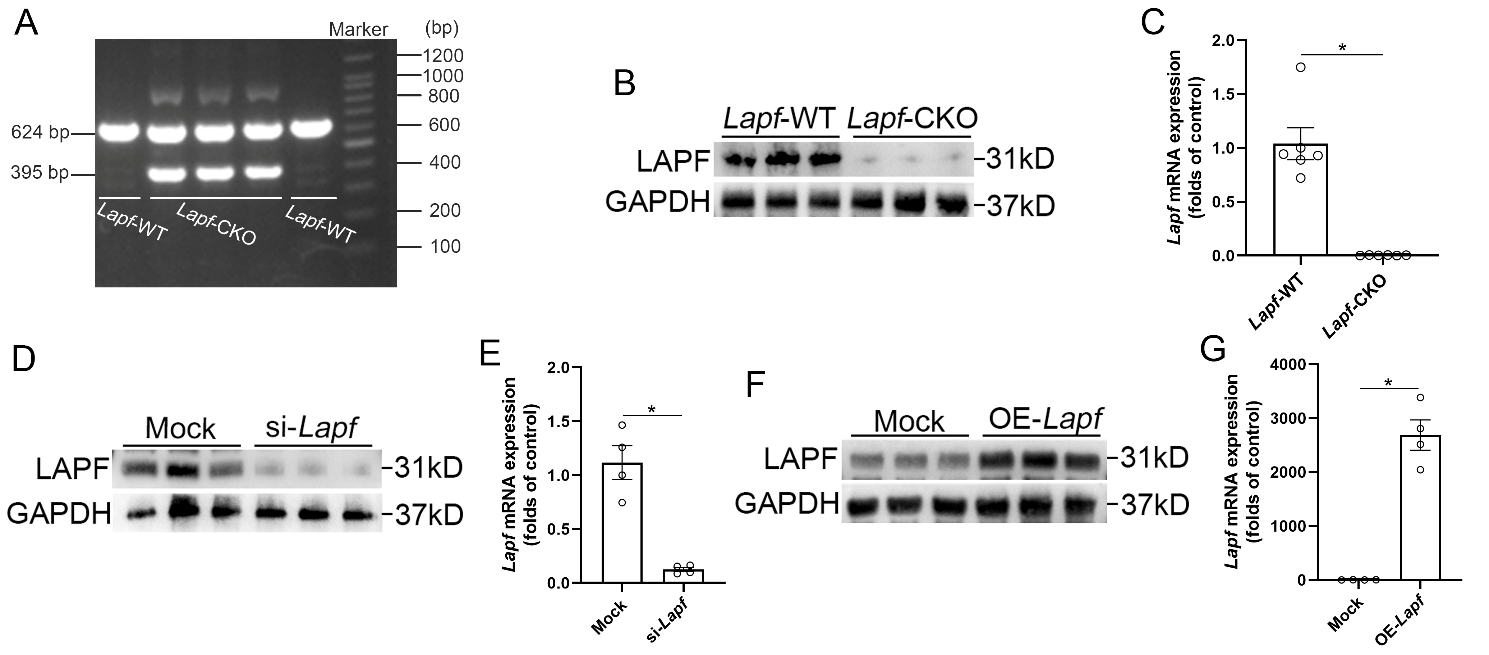


**Supplementary figure legend**

**Supplementary Figure 1 (A)** DNA gel electrophoresis to identify *Lapf*-WT and *Lapf*-CKO mice. **(B, C)** Efficiency of *Lapf* knockout in primary microglia by western blotting (B) and Q-PCR (C). **(D, E)** Efficiency of *Lapf* knockdown by western blotting (D) and Q-PCR (E). **(F, G)** Efficiency of *Lapf* overexpression by western blotting (F) and Q-PCR (G).
